# Flexible Graphene Field-Effect Transistors and Their Application in Flexible Biomedical Sensing

**DOI:** 10.1007/s40820-024-01534-x

**Published:** 2024-10-07

**Authors:** Mingyuan Sun, Shuai Wang, Yanbo Liang, Chao Wang, Yunhong Zhang, Hong Liu, Yu Zhang, Lin Han

**Affiliations:** 1 Institute of Marine Science and Technology, Shandong University, Qingdao, 266237 Shandong People’s Republic of China; 2 State Key Laboratory of Crystal Materials, Shandong University, Jinan, 250100 Shandong People’s Republic of China; 3 School of Integrated Circuits, Shandong University, Jinan, 250100 Shandong People’s Republic of China; 4 Shandong Engineering Research Center of Biomarker and Artificial Intelligence Application, Jinan, 250100, Shandong People’s Republic of China

**Keywords:** Flexible, Graphene, Field-effect transistor, Wearable, Implantable, Biosensor

## Abstract

Flexible electronics are transforming our lives by making daily activities more convenient. Central to this innovation are field-effect transistors (FETs), valued for their efficient signal processing, nanoscale fabrication, low-power consumption, fast response times, and versatility. Graphene, known for its exceptional mechanical properties, high electron mobility, and biocompatibility, is an ideal material for FET channels and sensors. The combination of graphene and FETs has given rise to flexible graphene field-effect transistors (FGFETs), driving significant advances in flexible electronics and sparked a strong interest in flexible biomedical sensors. Here, we first provide a brief overview of the basic structure, operating mechanism, and evaluation parameters of FGFETs, and delve into their material selection and patterning techniques. The ability of FGFETs to sense strains and biomolecular charges opens up diverse application possibilities. We specifically analyze the latest strategies for integrating FGFETs into wearable and implantable flexible biomedical sensors, focusing on the key aspects of constructing high-quality flexible biomedical sensors. Finally, we discuss the current challenges and prospects of FGFETs and their applications in biomedical sensors. This review will provide valuable insights and inspiration for ongoing research to improve the quality of FGFETs and broaden their application prospects in flexible biomedical sensing.

## Highlights

- The review provides a brief overview of the basic structure, operating mechanism, and key performance indicators of flexible graphene field-effect transistors.
- The review details the preparation strategy of flexible graphene field-effect transistors focusing on material selection and patterning techniques.
- The review analyzes the latest strategies for developing wearable and implantable flexible biomedical sensors based on flexible graphene field-effect transistors.

## Graphical abstract

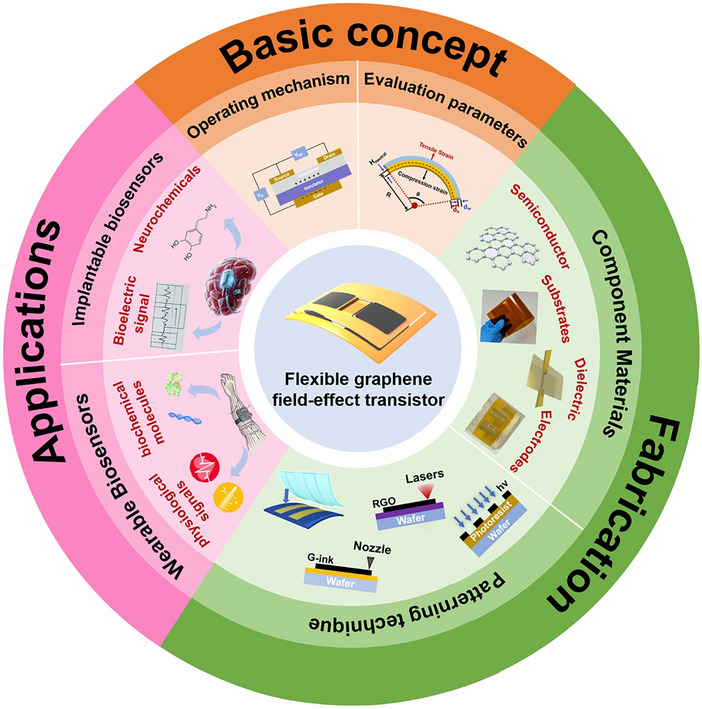

## Introduction

As the internet and IoT technologies evolve and become more prevalent, the demand for functional devices with capabilities such as physiological monitoring, voice control, eye tracking, gesture recognition, and motion sensing has grown, requiring greater portability, usability, and comfort [1–4]. Concurrently, there is an increasing consumer demand for health and fitness monitoring, driving the market towards high-performance, reliable, and user-friendly wearable and implantable devices [5–10]. In this context, flexible electronic devices, as an emerging class of electronics, are garnering significant attention due to their unique functional capabilities. These devices can operate effectively under irregular deformation conditions such as bending, rolling, folding, compression, and stretching [11–14]. Moreover, they offer advantages in terms of being lightweight, miniaturized, and comfortable [2, 15, 16]. Such attributes not only meet the growing market demands but also drive progress in the field of electronics and foster industry development, making flexible electronics a critical direction for modern scientific and technological advancement.

Field-effect transistors (FETs) are a core component of the modern electronics industry, recognized for their nano-scale fabrication, quick response times, signal amplification, and multi-parameter access, making them ideal for new, sensitive, portable biochemical detection platforms [17–22]. However, traditional FETs are primarily based on rigid substrates like silicon and flat glass, which inevitably limits their flexibility, stretchability, and range of applications. Organic materials, known for their inherent mechanical flexibility, low cost, and ease of processing, are considered ideal for flexible electronic devices [3, 23, 24]. In recent years, the mobility of organic semiconductors in FETs has markedly improved, rising from less than 10⁻^5^ cm^2^ V⁻^1^ s⁻^1^ to approximately 100 cm^2^ V⁻^1^ s⁻^1^ [25–29], a level comparable to many inorganic semiconductors. Despite these advancements, these materials remain sensitive to water and oxygen, which constrains their effectiveness in solution-phase sensing applications, such as environmental and biological monitoring [30, 31].

Graphene, a single-layer two-dimensional honeycomb lattice of carbon atoms linked through *sp*^2^ hybridization, is a promising alternative material with numerous advantages. Firstly, its remarkable electronic properties stem from its unique band structure. The symmetrical, cone-shaped valence and conduction bands are evenly distributed around the Fermi level, creating a zero-band gap semiconductor with the Dirac point at the intersection of the bands [32, 33]. This distinctive structure enables efficient electron transport, as graphene can be readily tuned from n-type (electron-dominated) to charge neutral, and further to p-type (hole-dominated) by adjusting the gate voltage [34, 35]. This tunability allows for precise control over the type and concentration of charge carriers, significantly enhancing the material's overall transport properties. Secondly, graphene has excellent gas and liquid barrier properties, a high surface area, and biocompatibility, which not only maintains stability but also supports a higher load of biological receptors, offering great stability and sensing potential [36, 37]. Furthermore, graphene’s exceptional chemical tunability allows for the formation of various derivatives, facilitating chemical modifications [38, 39]. These custom interface designs not only improve the selectivity of graphene-based biosensors but also significantly expand their applications in biometric detection. Lastly, graphene’s mechanical properties provide attractive prospects for wearable and implantable smart devices [40, 41]. Combining the advantages of graphene and FETs, flexible graphene field-effect transistors (FGFETs) are considered revolutionary innovations in flexible sensor technology, demonstrating many advantages in biosensing including ease of operation, fast response [42, 43], real-time monitoring [44–47], high specificity and sensitivity, low detection limits [48, 49], broad detection ranges [50, 51], and microfluidic integration [52–54]. Therefore, FGFETs and their applications in flexible biomedical sensors are garnering increasing attention. Through a literature review, we found that most existing reviews on flexible field-effect transistor electronics mainly focus on those based on organic materials [3, 55]. Although there are also some reviews on flexible GFET electronics, they are mostly outdated and do not systematically summarize the construction of FGFET electronics and their applications in flexible biomedical sensing [56, 57]. Given the advantages of flexible GFETs in the field of flexible electronics and the general interest in flexible sensors, along with recent significant advancements in this area, a specialized review of FGFETs and their applications in flexible biomedical sensing is necessary, which would be greatly beneficial not only to experts in electronics/semiconductor, material chemistry, soft matter science, and sensor technology but also to newcomers in these fields.

**Fig. 1:**
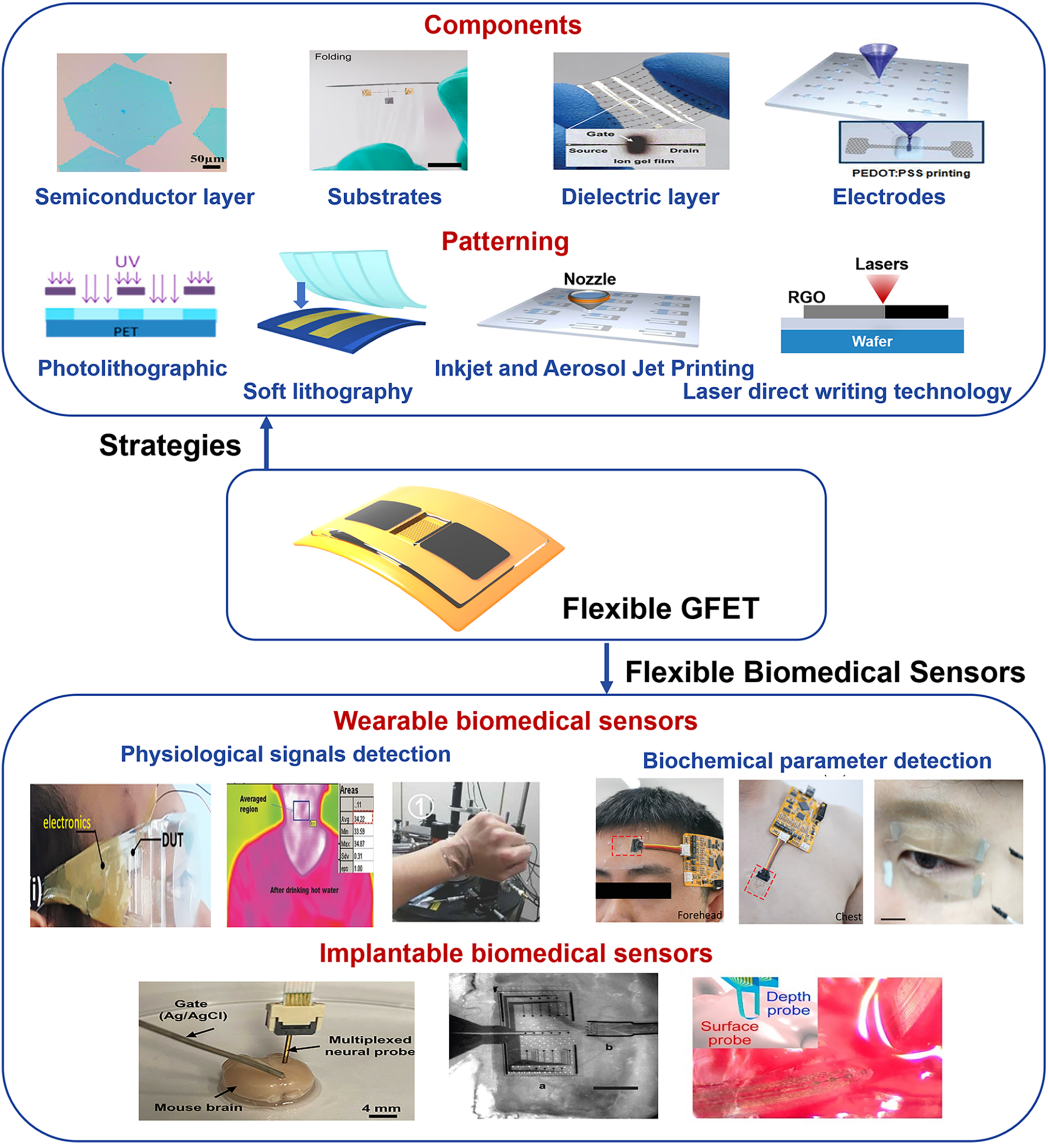
FGFET, including the construction of high-performance FGFET and their application in flexible biomedical sensing. Our focus is on high-performance components (graphene semiconductors [58], substrates [59], gate dielectrics [60], and electrodes [61]) and patterning methods (photolithography [62], soft lithography [63], inkjet printing [64], and laser direct writing) of FGFETs, and their applications in flexible biosensing including wearable FGFET-based biomedical sensors (physiological signal monitoring [65–67] and biochemical parameters detection [68, 69]) as well as implantable FGFET-based biomedical sensors [38, 70, 71]. Reproduced with permission from Ref. [58], Copyright 2020, Elsevier Ltd; Ref. [59], Copyright 2019, WILEY–VCH; Ref. [60], Copyright 2010, American Physical Society; Ref. [61], Copyright 2011, American Physical Society; Ref. [62], Copyright 2010, American Physical Society; Ref. [63], Copyright 2014, American Physical Society; Ref. [64], Copyright 2012, American Physical Society; Ref. [67], Copyright 2022, WILEY–VCH; Ref. [66], Copyright 2016, Wiley–VCH; Ref. [65], Copyright 2015, Elsevier Ltd; Ref. [68], Copyright 2021, WILEY–VCH; Ref. [69], Copyright 2021, American Association for the Advancement of Science

This review provides a detailed overview of the latest advancements in FGFETs, including the construction of high-performance FGFETs and their applications in flexible biomedical sensing (Fig. 1). It is important to note that the term "graphene" here encompasses all forms of graphene and its derivatives. To facilitate a better understanding of FGFETs, this paper begins with an introduction to the basic structure, operating mechanism, and evaluation parameters of these devices. Subsequently, we discuss the constituent materials and device patterning techniques for FGFETs, offering guidance on strategies for constructing high-performance devices. The FGFETs’ versatile strain sensing capabilities and sensitive response to bioelectrical signals make them highly adaptable for various applications. We particularly focus on the applications of FGFETs in wearable and implantable biosensing, highlighting key aspects of building high-performance flexible biosensors. Lastly, we explore the future developments in this field. It is worth noting that this review does not aim to exhaustively cover all literature related to FGFETs and FGFET-based flexible biomedical sensors. Instead, it emphasizes significant protocols, opportunities, trends, and challenges in this area. We hope that this discussion will be beneficial for future research aimed at realizing high-quality GFETs and their applications in flexible biomedical sensing.

## Introduction of FGFETs

### Structure and Operation Mechanism of FGFETs

**Fig. 2:**
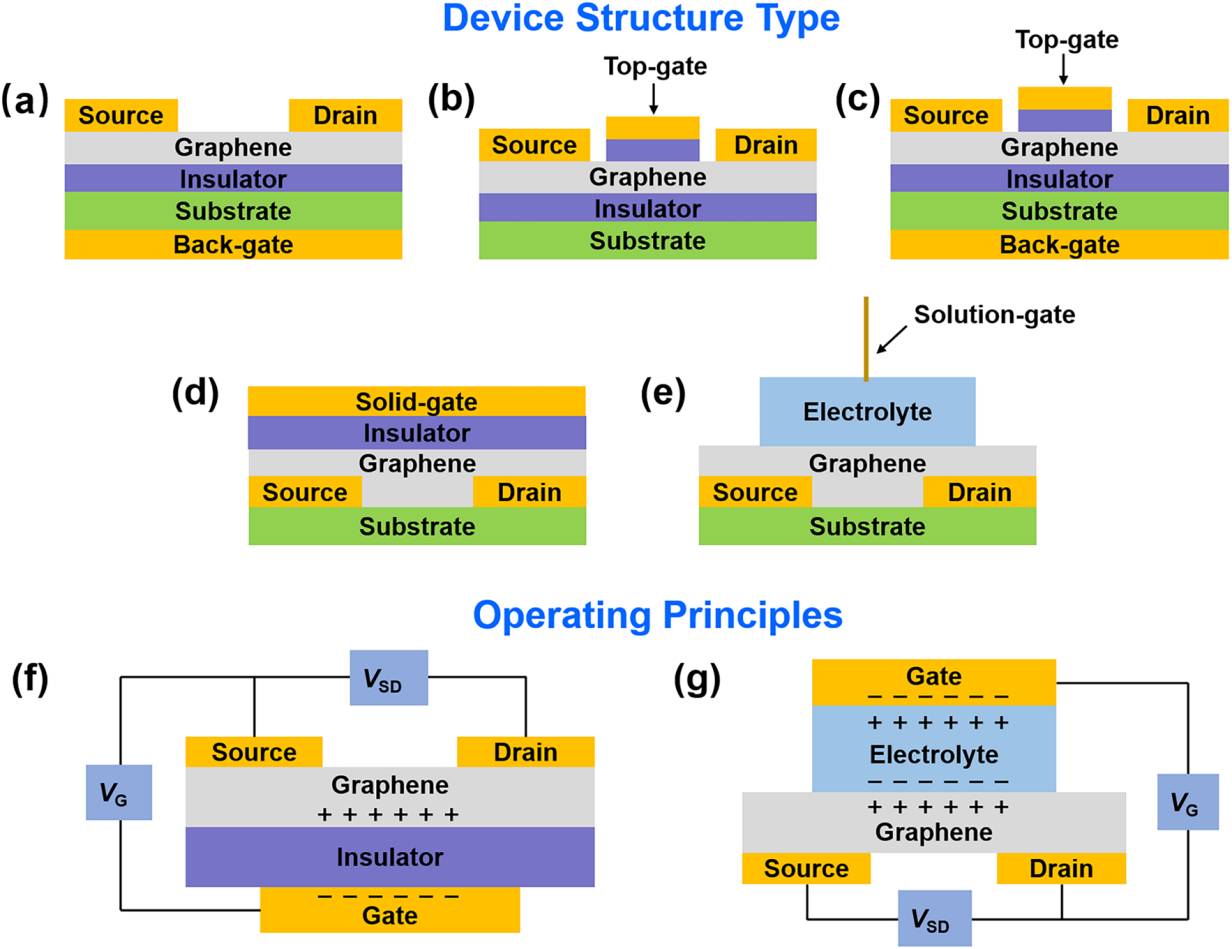
Schematic diagram of different graphene FET types. **a** Back-gated, **b** top-gated, **c** double-gated, **d** solid-gated, and **e** solution-gated GFETs. Operating principles of **f** solid-gated GFET and **g** solution-gated GFET

To fully understand FGFETs, it is essential to explore their structure and how they operate. FGFETs are typically three-terminal devices consisting of a flexible substrate, a dielectric layer, a graphene active layer, and source-drain and gate electrodes. These transistors come in several forms depending on the position of the gate: back-gated, top-gated, and double-gated, as depicted in Fig. 2a-c. Additionally, based on the type of gate dielectric used, they can be classified into solid-gated and solution-gated structures, illustrated in Fig. 2d, e.

As shown in Fig. 2f, solid-gate GFETs operate similarly to traditional silicon-based field-effect transistors. In these devices, the voltage applied to the gate modulates the charge density and type (either holes or electrons) in the channel via the gate insulator's capacitance. Different gate voltages create varied electric fields between the gate and the channel, inducing a space charge layer of equal magnitude but opposite polarity on each side of the gate insulation. When sufficient space charge accumulates, it forms a conductive path in the semiconductor layer. At a given source-drain voltage (*V*_SD_), charge flows from the source electrode through this conductive channel to the drain electrode, creating a current.

For solution-gated GFETs, which use an electrolyte solution as the dielectric instead of a traditional insulating material, the operation is slightly different. As depicted in Fig. 2g, applying a voltage between the gate and the source causes anions and cations in the electrolyte to migrate, forming two electrochemical double layers at the interfaces of the semiconductor/electrolyte and gate/electrolyte. This gate voltage modulates the potential in the channel via these double layers, establishing a channel of charge carriers (electrons or holes). Consequently, under the influence of *V*_SD_, a current flows from the source to the drain.

Both solid-gate and solution-gate GFETs control the concentration of carriers in the graphene layer by manipulating the gate voltage, which in turn influences the drain current. It is important to note that the carrier density and type (electrons or holes) in the GFET channel are dictated by the potential difference between the channel and the gate. A high positive gate voltage encourages electron accumulation, forming an n-type channel, whereas a high negative gate voltage results in a p-type channel.

### Evaluation Parameters of FGEETs

In general, the evaluation parameters for conventional FETs include threshold voltage (*V*_Th_), mobility (*μ*), switching ratio (*I*_on_/*I*_off_), and subthreshold swing (*SS*). However, due to the unique zero-bandgap structure of GFETs, these devices cannot achieve a true-off state. As a result, our discussion focuses on alternative key performance metrics for FGFETs, specifically *μ*, the Dirac point, and mechanical flexibility.

#### Device Mobility (μ)

*μ* represents the drift velocity of charge carriers per unit electric field, which indicates the ability of electrons or holes to move within a semiconductor under varying electric fields. There exists a positive feedback relationship between the speed of carrier movement and its mobility; that is, the faster the carriers move, the higher their mobility, and conversely, the slower they move, the lower their mobility. Transfer curves are a commonly used method to estimate mobility.

For devices operating in the linear region, the μ can be calculated using the slope of the transfer curve fitting curve with the Eq. (1), where *I*_SD_ represents the source-drain current, *L* the channel length, *W* the channel width, and *C*_*i*_ the capacitance per unit area of the gate dielectric layer.

1 $$\mu = \frac{L}{W{C}_{i}{V}_{\text{SD}}}\cdot \frac{{\partial I}_{\text{SD}}}{{\partial V}_{G}}$$

In the saturation region, the mobility can be derived from the slope of the tangent of the *I*_SD_^1/2^-*V*_G_ curve using Eq. (2). 

2 $$\mu = \frac{2L}{W{C}_{i}}\cdot {\left(\frac{\partial \sqrt{{I}_{SD}}}{{\partial V}_{\text{G}}}\right)}^{2}$$

Due to the unique electrical characteristics of graphene, GFETs are typically only weakly saturated. As a result, mobility calculations for GFETs usually rely on the formula for the linear region. Additionally, GFETs can transport both electrons and holes, thus exhibiting both electron and hole mobilities. High mobility means that GFETs have high-speed switching capabilities, high charge density, and low power consumption, expanding their applications in advanced electronic technologies. The mobility of GFETs is influenced by various factors, such as material quality, substrate effects, environmental conditions, electron scattering, doping, device structure, and dimensions. Understanding how these factors impact graphene mobility is essential for optimizing GFET design and performance.

#### Dirac Point

**Fig. 3:**
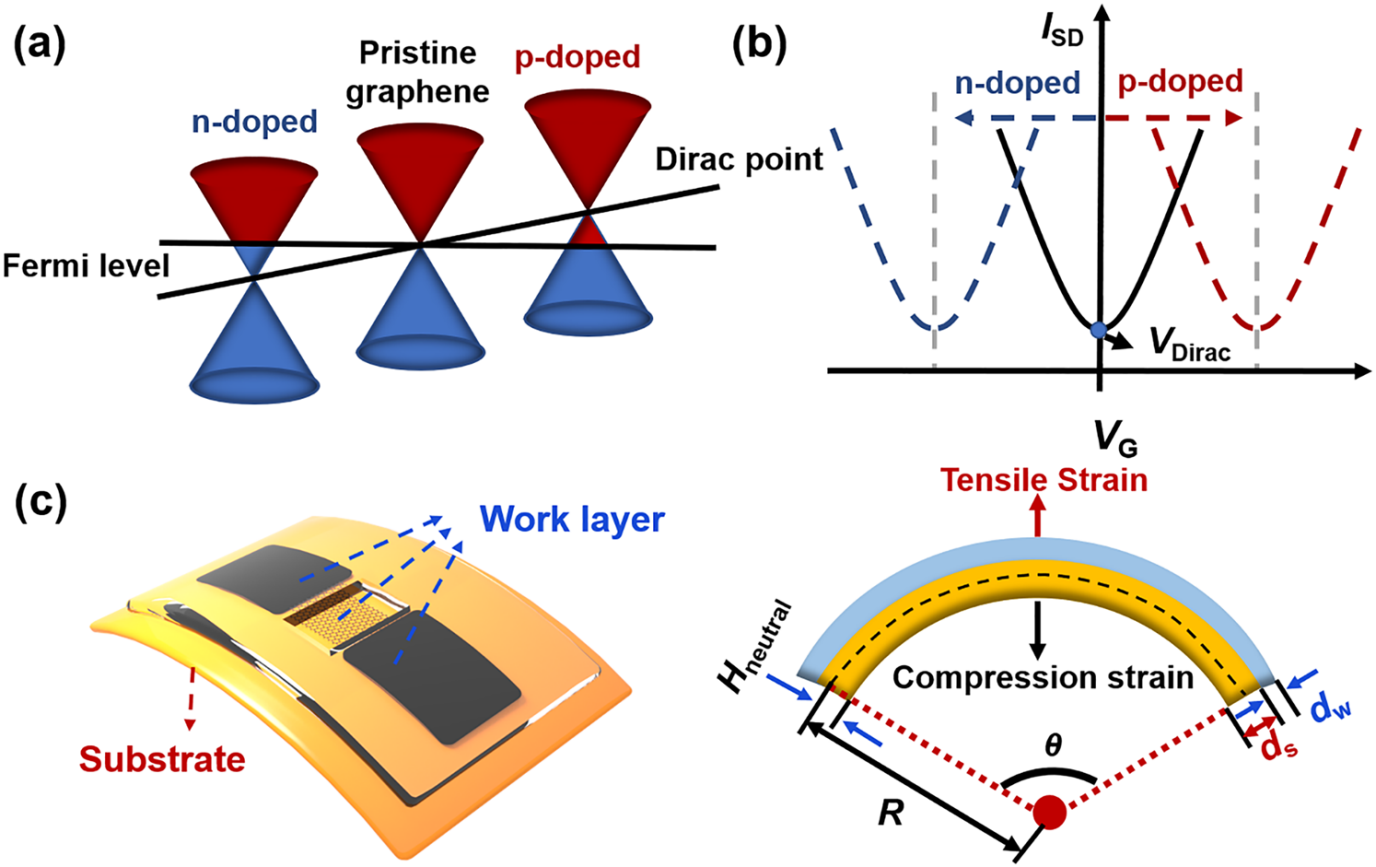
Evaluation parameters of FGFET. **a** Schematic representation of graphene Fermi energy levels and Dirac point positions as a function of dopant. **b** Variation of the corresponding device transfer curves/*V*_Dirac_ in different doping processes. **c** Strain diagram of flexible graphite field effect transistor

The Dirac point is an evaluation parameter unique to GFETs. In a graphene FET, the minimum conductivity point on its transport curve is the Dirac point (*V*_Dirac_), representing the potential where electrons and holes are equally abundant. In intrinsic graphene, the Dirac point corresponds to the energy level of the Fermi energy, close to 0 V on the transport curve. Typically, the presence of adsorbed molecules or defects induces doping effects, shifting the graphene Fermi surface above or below the Dirac cone point, as depicted in Fig. 3c. This shift transforms the dominant carriers into electrons or holes, primarily through charge transfer between the dopant and graphene. If the dopant's lowest unoccupied electron orbital is below graphene’s Fermi energy level, the charge transfers from graphene to the dopant, creating a p-type doping scenario. Here, the p-type branch of the GFET transport curve requires a higher gate voltage to turn on, moving *V*_Dirac_ to a more positive potential, as shown on the right side of Fig. 3d. Conversely, if the highest occupied orbital of the dopant is above the Fermi energy level, the charge transfers from the dopant to graphene, establishing n-type doping, which reduces the gate voltage to turn on the n-type branch of the GFET transport curve and shifts *V*_Dirac_ to a negative potential, as shown on the left side of Fig. 3d. The Dirac point in graphene is a crucial parameter for GFET-based sensors, with many such sensors detecting target molecules through doping effects [72–74].

#### Mechanical Flexibility

Mechanical flexibility is a crucial factor in assessing whether FGFET can maintain good performance parameters when subjected to bending, stretching, and twisting. Bending is the most common mechanical deformation, thus we focus here on the mechanical flexibility of flexible GFETs under bending deformation.

As illustrated in Fig. 3e, when FGFET is subjected to bending stress, a strain-neutral mechanical plane emerges within it that is neither tensile nor compressive [75, 76]. Thus, the material surface at this layer remains unaffected by strain. The surface above the neutral layer experiences tensile strain, while the surface below experiences compressive strain. Excessive tensile strain on the upper surface of the sensor beyond its tolerance limit may lead to material failure, while excessive compressive stress on the lower surface may cause detachment of certain structures on the device. Therefore, the design and fabrication of the sensor should ensure that the working layer is positioned as close as possible to the strain-neutral layer, minimizing strain on the working surface of the sensor during mechanical deformation.

For systems with complex multilayer composite structures like sensors, the distance between the strain-neutral layer and the substrate's lower surface (*H*_neutral_) is given by the following Eq. (3):

3 $${H}_{neutral}=\frac{{\sum }_{i=1}^{n}\frac{{E}_{i}}{1-{\nu }_{i}^{2}}{d}_{i}\left[\left(\sum_{j=1}^{i}{d}_{j}\right)-\frac{{d}_{i}}{2}\right]}{\sum_{i=1}^{n}\frac{{E}_{i}}{1-{\nu }_{i}^{2}}{d}_{i}}$$

where *n*, *d*_i_, *E*_i_, and *ν*_i_ are the total number of layers in the FGFET, the material thickness of the ith layer, Young's modulus, and Poisson's ratio, respectively.

The relationship between the distance from the working layer to the strain-neutral layer and the strain (*ε*) applied to the surface is given by:

4 $$\upvarepsilon =\frac{\left(R+{d}_{s}+{d}_{w}-{H}_{neutral}\right)\theta -R\theta }{R\theta }=\frac{{d}_{s}+{d}_{w}-{H}_{neutral}}{R}$$

where *d*_*s*_, *d*_*w*_, *H*_*neutral*_*,* and *R* represent the thickness of the substrate, the thickness of the working layer, the distance between the strain-neutral layer and the lower surface of the substrate, and the radius of curvature of the bend, respectively. The maximum ultimate strain represents the highest level of strain a device can endure without significant performance degradation or structural damage. A combined analysis of Eqs. (3) and (4) reveals that the position of the strain-neutral layer in an FGFET is determined solely by the material's physical properties and thickness, and is independent of the degree of bending deformation the flexible sensor experiences. In other words, variations in the bending radius do not affect the neutral layer's location, indicating that the maximum ultimate strain sustainable by the FGFET surface is pre-determined by material selection and structural design. Typically, in flexible GFETs, the working layer is much thinner than the substrate material, rendering its impact on the strain-neutral layer's position negligible. However, a thicker substrate shifts the neutral layer closer to itself, increasing the distance between the working layer and the neutral layer. This results in an increase in strain and a decrease in the maximum ultimate strain. Additionally, in FGFETs, the neutral layer tends to shift towards materials with a higher effective Young's modulus ($\frac{{E}_{i}}{1-{\nu }_{i}^{2}}$). Therefore, to position the working layer as close as possible to the neutral layer, the working layer's effective Young's modulus should be slightly higher than that of the base material. This implies selecting a working layer material with a higher Young's modulus and Poisson's ratio. However, these parameters should not be excessively high; they should be dynamically balanced with the substrate material to ensure the working layer aligns as closely as possible with the strain-neutral layer.

Thus, minimizing the thickness of each component is crucial in the design and fabrication of flexible GFETs. Furthermore, the Young's modulus and Poisson's ratio of the working layer stack should be slightly higher than those of the substrate material. These strategies maximize the ultimate strain that the FGFET can withstand while reducing the strain on the working surface during mechanical deformation.

## Strategies for the Preparation of FGFETs

Unlike conventional silicon-based GFETs, which primarily focus on electrical properties, FGFETs require a comprehensive trade-off between electronic properties and mechanical flexibility. The flexibility, ductility, and fatigue resistance of these devices place new demands on the materials used, necessitating that all components exhibit a requisite level of mechanical flexibility and strength. In addition, high-resolution patterning techniques directly affect device performance, such as output current and mobility, and are a key fabrication technique for preparing high-performance FGFETs. Therefore, this section will explore common patterning processes (photolithography, soft lithography, inkjet and aerosol jet printing, and laser direct writing) for high-performance components (including graphene semiconductors, substrates, gate dielectrics, and electrodes). These high-performance components and precise patterning techniques are foundational for constructing low-cost, compact, high-performance FGFETs on flexible substrates. The integration of these advanced fabrication methods ensures the development of FGFETs that meet the rigorous demands of modern flexible electronics, combining outstanding electronic functionality with essential mechanical properties.

### Components for FGFETs

#### Semiconductor Layer of FGFETs

It is well known that the semiconductor layer material is the core part of the field effect transistor and is closely related to the performance of the device. Therefore, the quality of the graphene material is particularly important for the GFETs. High-quality, high-throughput, large-scale, and cost-effective production methods are essential to balance ease of fabrication with the high quality of graphene.

To obtain high-quality pristine monolayer or multilayer graphene sheets, the most common method is micromechanical exfoliation of graphite with adhesive tape. This exfoliation method suffers from high preparation cost, low yield, difficulty in precise control, poor reproducibility, and difficulty in large-scale preparation, making it difficult to be integrated into FGFETs on a large scale. Although the epitaxial growth method can achieve high-quality growth of graphene with large area and high uniformity, its growth conditions are harsh and difficult to separate from the silicon substrate, and it is also not suitable for the preparation of flexible graphene FETs [77–79].

**Fig. 4:**
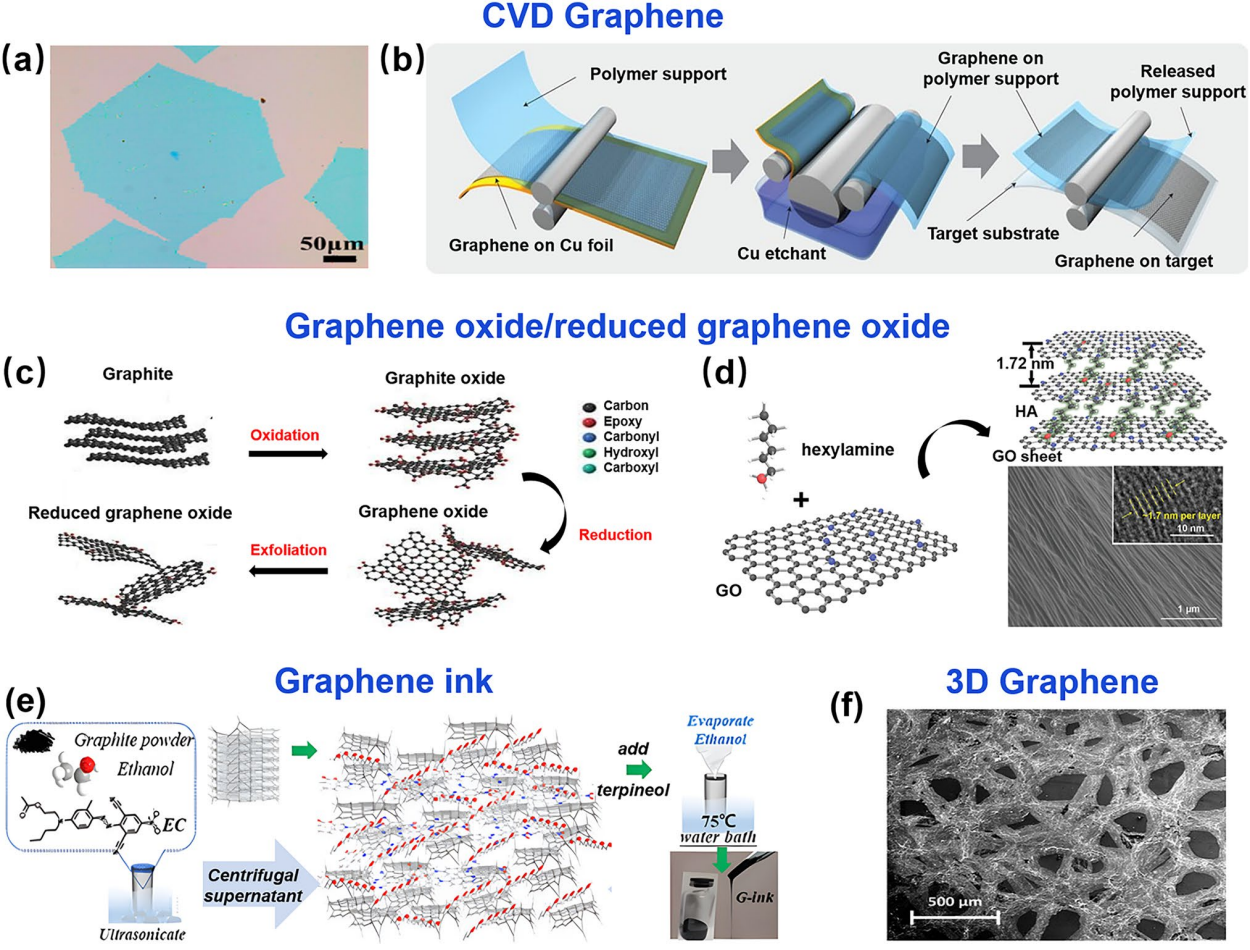
The semiconductor materials of FGFET. **a** Optical microscopy images of single-crystal graphene synthesized by CVD with staged oxidization/annealing process. Reproduced with permission from Ref. [58]. Copyright 2020, Elsevier Ltd. **b** Schematic diagram of roll-to-roll graphene film production method including adhesion of polymer supports, copper etching (rinsing) and dry transfer-printing on a target substrate. Reproduced with permission from Ref. [85]. Copyright 2010, Springer Nature. **c** Schematic chemical structures of graphene, graphene oxide, and reduced graphene oxide and route of graphite to reduce graphene oxide. Reproduced with permission from Ref. [39]. Copyright 2016, IntechOpen. **d** Schematic model of GO/hexylamine superlattice. SEM and TEM (upper right) images of GO/hexylamine superlattice. Reproduced with permission from Ref. [99]. Copyright 2021, WILEY–VCH. **e** Procedures for synthesizing graphene ink by modified liquid exfoliation method. Reproduced with permission from Ref. [100]. Copyright 2021, American Physical Society. **f** Scanning electron microscope (SEM) image of a 3-dimensional graphene foam. Reproduced with permission from Ref. [106]. Copyright 2016, WILEY–VCH

Chemical Vapor Deposition (CVD) method is the preferred method for large area synthesis of high-quality graphene. Hydrocarbons are cleaved into carbon and hydrogen atoms at high temperatures, the carbon atoms are deposited on the surface of metals such as copper or nickel sheets to form graphene films in this method [80–82]. The metal in this process is both a catalyst and a substrate for the growth of graphene. Graphene can be prepared on a large scale by this method. And the obtained graphene possesses high mobility and quality, while the number of layers can be controlled [78, 83]. Therefore, they are widely integrated as graphene channels in FGFETs [84]. Ning et al. [58] proposed a CVD cyclic oxidation/hydrogen annealing process that facilitates the reconstruction of copper foil surfaces, which in turn enables the growth of large single-crystal graphene (LSG) (Fig. 4a). Flexible FETs were formed on polyethylene terephthalate substrates using LSG, which exhibited excellent electrical properties and high bendability. The roll-to-roll (R2R) process is considered to be one of the most promising methods for fabricating electronic devices for large-area applications due to the relatively low production cost. Roll-to-roll (R2R) CVD fabrication has been proposed for continuous, large-area graphene film production. Sukang et al. [85] combined a roll-to-roll production method with the wet transfer process to achieve efficient etching and transfer of large-size (30-inch) graphene. (Fig. 4b) These films have sheet resistance as low as 125 Ω μm^−1^, have 97.4% optical transmittance, and exhibit high quality. The ultra-large area synthesis and roll-to-roll production of graphene offer better scalability and processability for graphene films, promising large-scale continuous production of FGFETs and flexible/stretchable electronics.

Graphene oxide (GO) is a derivative of graphene. It is prepared by oxidizing graphite using strong acids and oxidizing agents, followed by exfoliating the graphite layer by ultrasonic treatment, and finally purifying the exfoliated GO by ultracentrifugation (Fig. 4c) [86]. GO has advantages in terms of quantity, yield, and cost compared to CVD graphene. In addition, it possesses solution processing capabilities compatible with various flexible electronics preparation processes, such as spin-coating, spraying, printing, and adhesive coating methods [87–90], which allows its use as a channel layer for flexible FETs and is of great interest in the field of flexible electronics. However, the resulting GO is usually electrically insulating. To solve this problem, chemical or thermal reduction methods are commonly used to remove oxygen-containing groups from the surface of graphene oxide to obtain reduced graphene oxide (rGO) (Fig. 4d**)** [86, 91–93]. rGO not only retains the above-mentioned advantages of GO, but also substantially improves its conductivity. As a channel material for field-effect transistors, it shows electronic properties similar to those of graphene, such as those of bipolar transistors, such as bipolar transistor properties, making it seem to be a promising alternative material to graphene [91, 93, 94]. For example, He et al. [91] fabricated fully rGO thin-film transistors using a combination of solution-treated rGO electrodes and micropatterned rGO channels. This electronic device has perfect flexibility, high transparency, and typical bipolar properties, and shows good sensitivity in detecting proteins in physiological buffers.

Graphene oxide paper is a novel paper material made of single graphene oxide flakes that maintains excellent electrical properties and chemical stability in the format of GO nanosheets [95, 96]. Nevertheless, the large aggregation of 2D graphene flakes severely limits the field-effect sensing response [97], allowing the conductivity change of the GO paper to occur only within its nanometer-thick epidermal layer [98]. Therefore, the conductivity change of macroscopic materials up to hundreds of micrometers in thickness is negligible or even undetectable. In order to overcome the aggregation effect, Huang et al. [99] constructed 3D hybrid materials with alternating GO and hexylamine molecular layers (GO/hexylamine paper). This material has a layer-by-layer ordered superlattice structure that maintains stable electrical and field effect properties under mechanical stress (Fig. 4d). Together with the cost-effectiveness, robustness, and lightness of microthickness paper, this GO/hexamine superlattice material is expected to be used for a variety of flexible biochemical assays.

Due to the proliferation of various printing technologies, graphene ink, which is compatible with them, has been widely noticed and used as a semiconductor layer material for FGFETs in recent years [100, 101]. Unlike the chemical exfoliation method of graphene oxide, graphene ink is obtained by liquid phase exfoliation (LPE). This method treats graphite in solvent in ultrasonic waves and uses local hydrodynamic shear to achieve graphene exfoliation [102]. It has the advantages of inexpensive raw materials, high scalability potential, low thermal budget, and compatibility with additive manufacturing technologies, making it an ideal and cost-effective method for manufacturing graphene inks [103, 104]. Torrisi et al. [105] used N-methylpyrrolidone as a solvent to exfoliate graphite via LPE technology to produce graphene-based inks (Fig. 4e). Combined with inkjet printing technology, thin-film transistors, with mobilities of ∼95 cm^2^ V^–1^ s^–1^, as well as transparent and conductive patterns, with ∼80% transmittance and ∼30 kΩ μm^–1^ sheet resistance were prepared. The graphene-based ink opens the door to all-printed, flexible, and transparent graphene devices on a variety of substrates. Zhang et al. [100] also used a modified liquid exfoliation method to synthesize graphene ink (Fig. 4e) and combined it with an extruded direct ink writing (DIW) technique to prepare liquid gate graphene field effect transistors on PI substrates. Compared to the conventional ethanol-based liquid phase peeling method [103], the addition of pine alcohol to this modified method makes the graphene ink more compatible with printing methods by making it viscous through the exchange of ethanol solvent with pine alcohol.

Compared to 2D graphene, 3D graphene has a higher surface area and 3D structure, which will lead to better charge transport. Ameri et al. [106] created an ion gel-gated flexible 3D graphene transistor made from graphene foam (Fig. 4f). The device not only has a high current capacity of 26.72 times that of a 2D transistor, but is fabricated at room temperature and ambient pressure, making it suitable for realization on many flexible polymer substrates. As such, it is applicable to large-area electronics for displays, structural health monitoring and power systems.

In summary, the quality of graphene material plays a pivotal role in the performance of FGFETs. Traditional fabrication methods, such as mechanical exfoliation, although capable of providing high-quality graphene, exhibit significant limitations in terms of cost, yield, controllability, and scalability. Similarly, epitaxial growth methods have shown great potential in improving material quality, yet they still face challenges in terms of integration and flexibility. In contrast, CVD has emerged as the preferred technology for large-scale production of high-quality graphene due to its outstanding scalability, precise controllability, and seamless compatibility with modern device manufacturing processes. Particularly, R2R CVD technology has opened up new avenues for producing continuous, large-area graphene films, greatly enhancing the production scalability and processability of field-effect transistors, as well as flexible and stretchable electronic devices. Additionally, solution-phase graphene derivatives, such as GO, reduced graphene oxide, and graphene inks, demonstrate unique advantages in terms of yield, mass production, and solution processing, bringing additional versatility to the manufacturing of flexible electronic devices. Therefore, graphene prepared via CVD and its solution-phase derivatives emerge as the ideal choice for fabricating high-performance flexible GFETs, offering robust technical support and broad application prospects for future electronic devices.

#### Substrates of FGFETs

**Fig. 5:**
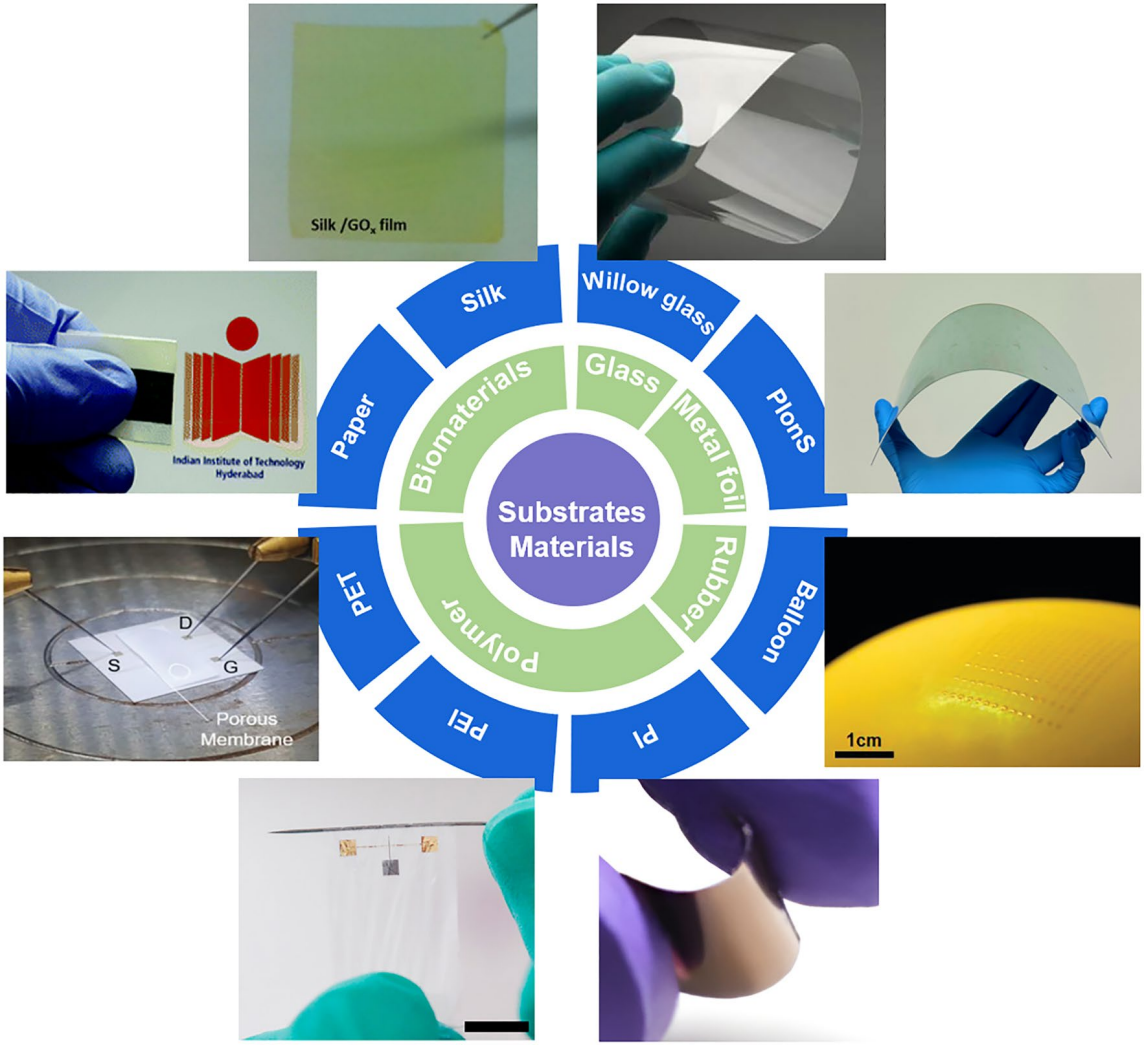
The substrate materials of FGFET. Clockwise from right top: willow glass, PIonS, balloon [61], polyimide (PI) [115], polyethylene glycol terephthalate (PET) [110], polyethylenimine (PEI) [59], paper [112], silk [113]. Reproduced with permission from Ref. [61], Copyright 2011, American Physical Society; Ref. [115], Copyright 2017, American Physical Society; Ref. [110], Copyright 2022, American Physical Society; Ref. [59], Copyright 2019, WILEY–VCH; Ref. [112], Copyright 2016, RSC Publishing; Ref. [113], Copyright 2014, Elsevier Ltd

Flexible substrates play a central role in the design and functionality of FGFETs, providing not only physical support for the devices but also directly influencing the device fabrication process and the final flexibility performance. To ensure the excellent bending performance of FGFETs, the chosen substrate material must demonstrate outstanding mechanical stability, capable of withstanding repeated bending and stretching. Additionally, the thermal stability and surface smoothness of the substrate material are crucial for maintaining good contact between the substrate and other components, as well as compatibility with conventional device fabrication processes. As shown in Fig. 5, currently reported FGFET substrates include thin glass [107, 108], metal foil [109], rubber [61], polymers [110, 111], biodegradable biomaterials [112–114], etc.

Polymer substrates have a number of natural advantages that make them the most common flexible substrates. Among them, polyethylene glycol terephthalate (PET) and polyethylene naphthalate (PEN) are widely used in early FGFETs because of their advantages in spatial stability, moisture absorption, transparency, chemical corrosion, and cost [59, 116–119]. However, the low upper working temperature and high surface roughness are their biggest drawbacks, which make them difficult to be compatible with fine manufacturing processes and fabricate microscale devices. Compared with PET and PEN, the polydimethylsiloxane (PDMS) has better thermal stability that glass transition temperature is as low as 125 °C and the coefficient of thermal expansion (CTE) is 301 ppm °C^−1^ [120]. In addition, its easy availability, chemical stability, low Young’s modulus (≈3.7 MPa), skin-friendliness, and good adhesion to electronic materials make it a good choice for E-skin applications [61, 121]. However, PDMS is permeable to aqueous solutions [122], which causes it to swell in liquid environments and damage metal parts of the device [123], thus limiting its application in biosensors. The properties of polyimide (PI), with a higher glass transition temperature (360–410 °C) [124] and a lower CTE (16 ppm °C^−1^) and low Young's modulus (≈2.8 GPa) [125], are better than PDMS. In addition, it has long-term stability and good biocompatibility [126], so flexible PI-based devices can be used for implantation [101, 111, 127]. In addition, polyethersulfone (PES) [92, 128], polyethylene (PE) [129] and polyethylenimine (PEI) [121] have also been used as flexible substrates of the FGFETs. Although the above polymer substrates are well suited to meet the repeated bending and stretching requirements of flexible GFETs, they take a long time to biodegrade, which can lead to serious environmental pollution problems. Therefore, cellulose paper [112, 130], microbial cellulose [131] and silk substrates [110, 114] have also been investigated as substrates of FGFETs because they are biodegradable, flexible, lightweight, cost-effective, breathable, comfy, and durable [132].

In a word, in selecting substrate materials for FGFETs, it is crucial to consider both application requirements and material properties comprehensively. Choosing the right substrate can optimize the overall performance of FGFETs to meet the demands of specific application areas. For wearable electronic devices, key attributes include high flexibility, good biocompatibility, chemical resistance, and skin-friendliness. Consequently, PDMS is widely used in electronic skin and similar devices due to its excellent flexibility and low cost. PI is particularly suitable for long-term skin contact applications due to its high thermal stability and exceptional biocompatibility. For high-performance flexible electronics that require heat resistance and mechanical stability, PES and PI exhibit superior thermal stability, making them ideal choices. Environmental sensors need materials that offer chemical stability, environmental tolerance, and cost-effectiveness. In this context, PEI and PI are preferred due to their robust chemical and thermal stability, which maintains performance under various environmental conditions. In the field of biomedical sensors, biocompatibility, chemical stability, and gentle mechanical properties are essential. PI is suitable for implantable devices, while microbial cellulose, with its biodegradability and mild mechanical properties, fits temporary biomedical applications well. For eco-friendly electronic products, biodegradability, low environmental impact, and cost-effectiveness are central considerations. Cellulose paper and silk substrates, due to their low cost and good biodegradability, are suitable for eco-friendly disposable electronic products.

#### Dielectric Layer of FGFETs

The dielectric layer is also an important factor affecting the performance of FGFETs. On the one hand, in order to prevent current leakage between the gate and the semiconductor channel and to ensure the low voltage operation of the FGFET, the dielectric layer material of the FGFET should have a high resistance coefficient and a high dielectric constant. On the other hand, in order to ensure that the device is not easily damaged when mechanical deformation occurs, the dielectric layer material should also have good mechanical flexibility.

**Fig. 6:**
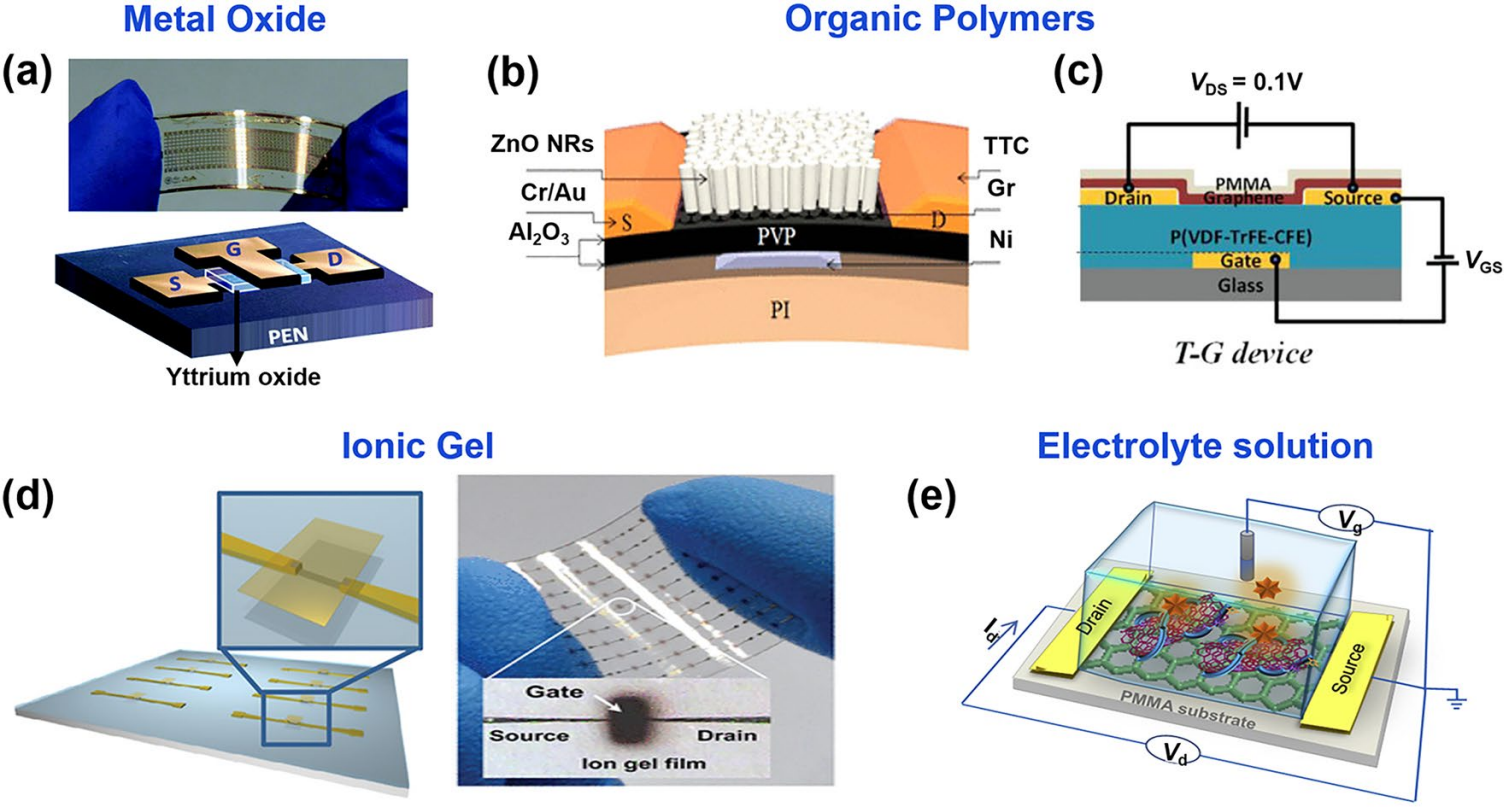
The dielectric layer materials of FGFET. **a** Schematic diagram of a flexible GFET with a dielectric layer of yttrium oxide. Reproduced with permission from Ref. [136]. Copyright 2015, RSC Publishing. Schematic diagram of a flexible graphene GFET with organic polymer **b** Al_2_O_3_/PVP/Al_2_O_3_ [151] and **c** P(VDF-TrFE-CFE) [149] as a dielectric layer. Reproduced with permission from Ref. [151], Copyright 2015, American Physical Society, and Ref. [149], Copyright 2020, WILEY–VCH. **d** Optical images of an array of GFET devices with a dielectric layer of ion gel film. Reproduced with permission from Ref. [60]. Copyright 2010, American Physical Society. **e** Schematic diagram of a solution-gated FET with a dielectric layer of PBS solution. Reproduced with permission from Ref. [159]. Copyright 2018, Elsevier Ltd

Metal oxide dielectrics (Al_2_O_3_, ZrO_2_, HfO_2_, and Y_2_O_3_) are applied in flexible GFETs to obtain relatively high electrical performance due to their high dielectric constants, chemical stability, low breakdown resistance, and high-temperature resistance (Fig. 6a) [115, 119, 131, 133–136]. However, the brittle and rigid nature of the metal oxide limits it to about 2% tensile strain, and subsequent mechanical cracking may lead to current leakage, causing it to fail at high strains [137, 138].

In contrast, organic polymer dielectrics have several built-in benefits, such as low-temperature solution processing, low film density, high leakage current density, strong compatibility with flexible substrates, customizable molecular structure [139–141], making it possible to include polymethyl methacrylate (PMMA) [142], polyxylene [119], polyimide (NPI) [143], poly-4-vinyl phenol and poly (melamine-co-formaldehyde) (PVP/PMF) [144], poly(1,3,5-trimethyl-1,3,5-trivinylcyclotrisiloxane) (pV3D3) [145] within the polymeric insulating materials are widely used for flexible GFETs. However, these polymer dielectrics generally have low dielectric constants (κ < 3.0) compared to inorganic rigid dielectrics. As a result, their use as dielectric layers leads to a weaker dielectric shielding effect. This reduced shielding makes the carriers in graphene more prone to scattering by impurities, defects, or phonons, thereby decreasing the mobility of the GFET [146–150]. Additionally, dielectric layers with low dielectric constants require a higher electric field strength per unit voltage to achieve the desired electric field. Consequently, the gate voltage must be increased to reach the necessary field strength, which in turn raises the operating voltage of the GFET. This increased operating voltage does not align with the low energy requirements of wearable electronics. To solve these problems, Dang et al. [151] combined organic polymers with metal oxide dielectrics to prepare the flexible hybrid organic–inorganic (Al_2_O_3_/PVP/Al_2_O_3_) dielectric layer (Fig. 6b), which improves the electrical properties of the device to some extent while retaining good flexibility. In addition, Wen et al. [149] applied the high-κ polymer P(VDF-TrFE-CFE) in the dielectric layer of graphene FETs (Fig. 6c). The overall performance of the GFETs with P(VDF-TrFE-CFE) dielectric is considerably improved compared to common dielectrics (SiO_2_ and PMMA), with hole mobility of 3.46 × 10^3^ cm^2^ V^–1^ s^–1^, electron mobility of 2.26 × 10^3^ cm^2^ V^–1^ s^–1^, and on/off ratio of 4.28. Furthermore, the devices have good adaptability to flexible substrates. This research fills the gap in the research topic of GFETs with organic high-κ dielectrics.

Ionic gel is a kind of gel electrolyte based on ionic liquid. Generally, it is formed by adding an ionic liquid as additives to the polymer framework, in which 1-ethyl-3-methylimidazolium bis(trifluoromethylsulfonyl)imide ([EMIM][TFSI]) and various polymers such as enolate (PEGDA) monomer, pentaerythritol tetraacrylate (PETEA), and poly(styrene block methyl methacrylate-block styrene) (PS-PMMA-PS) are often used as ionic liquid and the polymeric framework, respectively [106, 152, 153]. In addition to excellent mechanical flexibility and optical transparency, they have very high capacitance values [60, 61, 154, 155], and can be processed in solution at room temperature, which makes them ideal for use in flexible plastic substrates. Therefore, it is an ideal dielectric material for flexible field effect devices [60, 106, 156], Jin et al. [60] used ionic gels as gate dielectrics to fabricate low-voltage working graphene FET arrays on plastic substrates (Fig. 6d). Due to the extremely high capacitance of the ionic gel (5.17 μF cm^−2^), high on-state current and low voltage operation are provided for the graphene FET. And it also shows very good mechanical flexibility, with only a 20% change in *μ*/*μ*_0_ as the bend radius changes from 6 to 0.6 cm.

In addition to the dielectric materials mentioned above, there is a special gate dielectric layer, the electrolyte solution. It is often used to prepare FGFET because it not only greatly increases the gate capacitance and provides lower operating voltage and higher electrical performance for the device, but also its fluidic nature makes it good for flexibility [157]. We usually refer to transistors with solution dielectrics as solution-gated field-effect transistors, and their structural simplicity and stability in aqueous solutions make them good candidates for detecting solution-phase molecules. In solution-gated GFETs, the ionic concentration of the electrolyte solution is a critical factor. Increasing the ion concentration leads to near-complete neutralization of impurity charges by the ions, which brings the effective net impurity charge close to zero. This neutralization significantly reduces scattering caused by charged impurities, thereby enhancing the carrier mobility in GFETs [158]. Consequently, adjusting the content and concentration of the electrolyte can effectively modulate the performance of solution-gated GFETs [146, 158]. The most commonly used electrolyte solution is PBS, and Majd et al. [159] fabricated a solution-gated FET with a phosphate buffer solution at pH = 7.5 (Fig. 6e). This solution-gated graphene field effect transistor provided a lower operating potential compared to the reverse gated device. Notably, in solution-gated field-effect transistor-based biosensing, biomolecules are subjected to ion screening due to the presence of mobile ions in the solution, called the Debye screening effect. Higher ion concentrations will result in a stronger Debye screening effect, i.e., a shorter Debye length. Beyond this length, the charge is effectively shielded, with only 36.8% of the charge still visible to the graphene sensing device, which can significantly degrade the sensing performance of the biosensor. The issues related to the Debye length are described in detail in the review "Sensing at the Surface of Graphene Field-Effect Transistors” [160]. Therefore, considering the Debye screening effect and its impact on biosensor performance, proper selection and adjustment of the concentration of the electrolyte solution is crucial in optimizing the performance of solution-gated FGFETs.

In summary, the dielectric layer plays a pivotal role in defining the performance and reliability of FGFETs, with each type of dielectric layer having its unique characteristics and suitability for different applications. Metal oxide dielectrics are widely used due to their high dielectric constant and stability, but their brittleness and rigidity limit their use in flexible devices. Conversely, organic polymer dielectrics offer flexibility and processability, but their lower dielectric constant increases the operating voltage and reduces carrier mobility. Hybrid organic–inorganic dielectric layers combine the advantages of both materials, enhancing electrical performance while maintaining flexibility. Additionally, ionic gels provide mechanical flexibility, optical transparency, and high capacitance, making it easy to integrate into flexible plastic substrates and enabling FGFETs to operate at low voltages with high on-state currents. Solution-gated FGFETs using electrolyte solutions offer adjustable device performance and show great promise in biosensing applications, though the electrolyte concentration must be carefully considered to mitigate Debye screening effects and optimize sensing performance. Advances in dielectric materials and structures have significantly propelled the development of high-performance, flexible graphene-based electronic devices across various applications.

#### Electrode of FGFETs

Flexible electrodes, another important research consideration in FGFET design, have also attracted significant research interest. The ability to smoothly inject carriers from the electrode into the semiconductor layer is a fundamental requirement for flexible FET electrodes, involving both good, tight physical contact between the electrode material and the graphene semiconductor, and a good energy level match between the two. Low and consistent contact resistance will be possible as a result, which will enhance charge injection between the semiconductor and the electrode. In addition, the flexibility of the electrodes is also an issue to be considered in order to achieve high performance FGFETs.

**Fig. 7:**
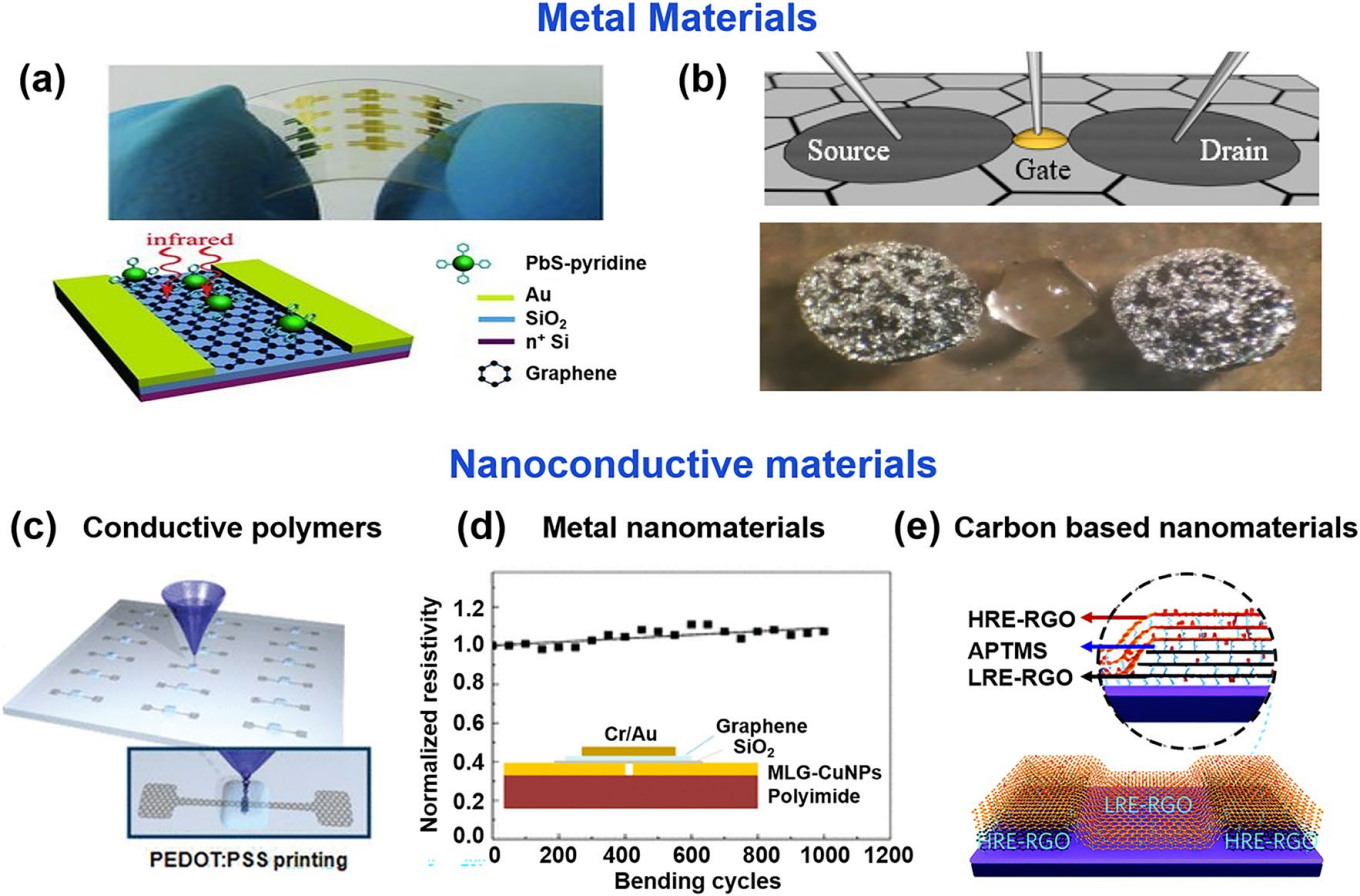
The electrode materials of FGFET. Schematic diagram of a flexible graphene GFET with metal electrodes **a** Au [116] and **b** liquid–metal [171]. Reproduced with permission from Ref. [116], Copyright 2012, WILEY–VCH and Ref. [171], Copyright 2019, Multidisciplinary Digital Publishing Institute. Schematic diagram of a flexible graphene GFET with nanoconductive materials electrodes **c** polystyrene sulfonate conductive polymers [61], **d** graphene-encapsulated copper nanoparticles (MLG-CuNPs) metal nanomaterials [115], and **e** reduced graphene oxide (rGO) [199]. Reproduced with permission from Ref. [61], Copyright 2011, American Physical Society; Ref. [115], Copyright 2017, American Physical Society; Ref. [199]. Copyright 2020, Royal Society of Chemistry

Indium tin oxide (ITO) films, known for their electrical conductivity and optical transparency, have been widely used in optoelectronics for decades [161]. However, ITO films are hard and brittle, and can easily break when subjected to small tensile strains, which limits their application in FGFETs [106, 161, 162]. Currently, the most prevalent electrode material in flexible graphene transistors is gold (Fig. 7a). In order to reduce the contact resistance between the gold electrode and graphene, a layer of Ti, Cr, Ni, and other metals with Fermi energy levels closer to the function of graphene is normally deposited first. This operation not only reduces the contact resistance between the two but also improves the adhesion of the gold electrode so that it is not easy to fall off. Although conventional metallic materials serve as electrodes with good electrical conductivity, they suffer from a very high Young's hardness, which increases the hardness of the built devices. Although small mechanical bending and stretching can be withstood by reducing the thickness of the metal film, due to the Ostwald ripening effect, the reduction in metal film thickness usually causes the creation of isolated metal islands, thus sacrificing conductivity [163, 164]. Therefore, when using metals as electrodes, the thickness of the appropriate film should be selected to balance the electrical and mechanical properties of the FGFET. Liquid metal, an indefinite and flowable special metallic material, has been studied as a candidate for flexible device electrodes because of its high electrical conductivity, high flexibility (Young's modulus is almost zero) and non-toxicity [165–169]. Generally, it is prepared by a low-temperature melting process, where different metallic materials (mainly gallium and indium alloys as base materials) are formed in a certain ratio and fully melted by temperature control [170]. In combination with sprayed stencil technology, Melcher et al. [171] have successfully integrated it as an electrode in a flexible top-gated GFET (Fig. 7b) with the hole and electron mobilities of 663.5–689.9 cm^2^ V^–1^ s^–1^, respectively.

Unlike conventional materials, nanomaterials are characterized by macroscopic quantum tunneling effects, surface and interface effects, quantum size effects, and small size effects, which give them unique optical, electrical, magnetic, thermal, and mechanical properties. Consequently, nano-conductive materials such as conductive polymers (TC), metal nanomaterials (metal nanoparticles (NPs) and metal nanowires (NWs)), carbon-based materials (graphene and carbon nanotubes (CNTs)) can be dissolved in inexpensive solvents by relatively simple methods to prepare flexible electrodes by printing, spin coating, and spraying. It has drawn a lot of attention in the field of the preparation of flexible electronic devices as their low temperature and non-vacuum operation, low raw material cost can compensate for some defects in macroscopic materials.

Conductive polymers (CP), including polypyrrole, polythiophene, polyaniline and poly(3,4-ethylenedioxythiophene) polystyrene sulfonate (PEDOT: PSS), are commonly used as flexible electrode materials [133, 172, 173]. The electrical and mechanical properties of these conducting polymers can be fine-tuned depending on their microstructure, synthesis method and doping process [174–176]. Among them, PEDOT: PSS is the most widely used CP in flexible electronics due to its good electrical stability, mechanical strength, optical transmittance, film homogeneity and biocompatibility [177–179]. Li et al. [61] constructed an all-graphene FET on a rubber substrate using PEDOT: PSS (conductivity of 100 S cm^−1^) as the gate (Fig. 7c). All-graphene FETs exhibited good electrical properties with hole and electron mobility rates of 1188 ± 136 and 422 ± 52 cm^2^ V^−1^ s^−1^, and mechanically stable, even after 1000 or more cycles, at stretching degrees up to 5%.

Metal nanoparticles (mNPs) and metal nanowires (mNWs) can achieve higher electrical conductivity compared to conductive polymers. In addition, it also exhibits improved mechanical flexibility and stretchability [180, 181]. Precious metal particles, such as gold nanoparticles (AuNPs), platinum nanoparticles (PtNPs) and silver nanoparticles (AgNPs) have attracted great interest due to their high electrical conductivity and excellent electrochemical activity [101, 182, 183]. However, the high cost of these precious metals hinders their practical applications. Copper nanoparticles (CuNPs) are considered promising candidates to replace most noble metals because of their relatively low cost, non-toxic elements and high electrical conductivity [22]. Zeng et al. [115] designed multilayer graphene-encapsulated copper nanoparticles (MLG-CuNPs) with excellent electrical and mechanical properties (Fig. 7d). The resistivity of MLG-CuNPs/PI increased to 1.7 × 10^–6^, which is 100 times lower than that previously reported [184], and its resistivity increased by only ∼7% after 1000 bending cycles. The MLG-CuNPs were used as source/drain electrodes to fabricate the GFET on the flexible PI substrate, showing hole and electron mobilities of ∼940–∼800 cm^2^ V^–1^ s^–1^, respectively.

Metal nanowires (mNW) not only have the advantages of low thin layer resistance, visual transparency, ease of use and low cost [185, 186], and due to their relatively large aspect ratio, have good mechanical compliance [187–190]. Therefore, it shows crucial promise in flexible transparent electrodes/conductors as well as in flexible stretchable electrodes. The most commonly used mNW is AgNW [185, 188]. However, the single mNW also has some drawbacks, such as the limitation of mNW percolation network in reducing its pattern size and weakness of mNW to electric breakdown, chemical oxidation, and high thin layer resistance [185, 191–193]. Graphene–mNW hybrid nanostructures can make up for the shortcomings of mNWs single material. Moreover, with their low resistance (< 35 Ω sq^−1^) and high light transmission (> 90% in the visible range) as well as their excellent mechanical flexibility and stretchability, they have attracted considerable attention [194–196]. Jin et al. [197] used a graphene-AgNW hybrid film as source/drain electrodes to prepare a GFET. Graphene penetrated all the conducting elements of the device, including the channel and the electrode, the contact resistance between the channel and the source/drain was reduced, which improved the mobility of the field-effect transistor (≈3000 cm^2^ V^−1^ s^−1^). The high elastic modulus of graphene and AgNW and the hydrophobicity of parylene enable their transfer to various substrates. This is an exciting approach for the fabrication of transparent wearable electronic devices.

Carbon-based nanomaterials, including carbon nanotubes (CNTs) and graphene, are attractive electrode materials for FGFET due to their high electrical conductivity, mechanical strength, and excellent optical transparency [198]. Among them, multi-walled carbon nanotubes (MWNTs) are relatively suitable as gate electrodes due to their unique characteristics, including high surface area, corrosion resistance, electrochemical stability, high electrical conductivity, and relatively lower manufacturing costs. For example, Liu et al. fabricated a fully carbon-based FET on a flexible polyethylene terephthalate substrate using spray-jet printing. Conductive reduced graphene was used as electrodes (source and drain) and channel, while oxidized graphene served as the dielectric, and multi-walled carbon nanotubes were used as gate electrodes. The FET exhibited a favorable mobility of 350 cm^2^ V^–1^ s^–1^. Reduced graphene oxide, due to its varying degrees of reduction, exhibits different properties, thus can be used as both a semiconductor layer and an electrode. For instance, Zhang et al. [199] proposed a fully covalent-based FET, which was prepared by covalently layer-by-layer assembling oxidized graphene with (3-aminopropyl) trimethoxysilane (APTMS) coupling agent and subsequently reduced at different temperatures (Fig. 7e). Films of rGO with relatively high and low reduction degrees/conductivities (abbreviated as HRE-RGO and LRE-RGO) were used as source/drain electrodes and semiconductor layers of the device, respectively. Unlike FETs prepared by traditional physical deposition methods, the covalent structure of the FET endows it with excellent stability. Even after continuous ultrasound treatment in 10 common solvents for 100 min (total 1000 min), the electrical characteristics of the device fluctuated insignificantly. Therefore, it is less prone to degradation or fluctuation during subsequent multi-step fabrication and biological testing processes, and is expected to shine in the application of FGFET.

In the design of FGFETs, choosing the right electrode material is crucial for device performance. Metal electrodes like gold, silver, and copper are highly conductive, making them ideal for high-performance electronic devices and precision sensors; however, their poor flexibility can lead to fractures under bending. In contrast, liquid metal electrodes, particularly those based on gallium alloys, offer exceptional flexibility and ductility, making them highly suitable for ultra-flexible and stretchable electronics. Despite this, they may require special encapsulation techniques to prevent metal leakage or corrosion. Additionally, nanomaterial electrodes (conductive polymers, metallic nanomaterials, carbon-based nanomaterials) provide good conductivity and transparency, making them well-suited for transparent electronics and flexible displays, and they also possess good biocompatibility for biomedical devices. Nevertheless, these materials face challenges related to uniformity and repeatability during manufacturing. Therefore, the selection of electrode materials must balance electrical performance, mechanical properties, and cost-effectiveness to ensure suitability for specific applications and environments.

### Patterning Process for FGFETs

Patterning technologies are an important part of the fabrication of graphene-based nanodevices and directly affect the performance of the devices. Patterning during the preparation of FGFETs, which includes patterning of multiple components of graphene semiconductor layers, electrodes, and gate dielectrics, follows the basic idea of removing material from top to bottom or adding material from bottom to top during the fabrication process. The characteristics of flexible electronics should also be considered when drawing on patterning techniques for microelectronic and micro-electro mechanical system (MEMS) devices. Flexible electronics require large-area, low-temperature, low-cost patterning techniques. Commonly used patterning techniques for FGFETs include photolithography, soft lithography, inkjet and aerosol jet printing, laser direct writing.

#### Photolithographic

**Fig. 8:**
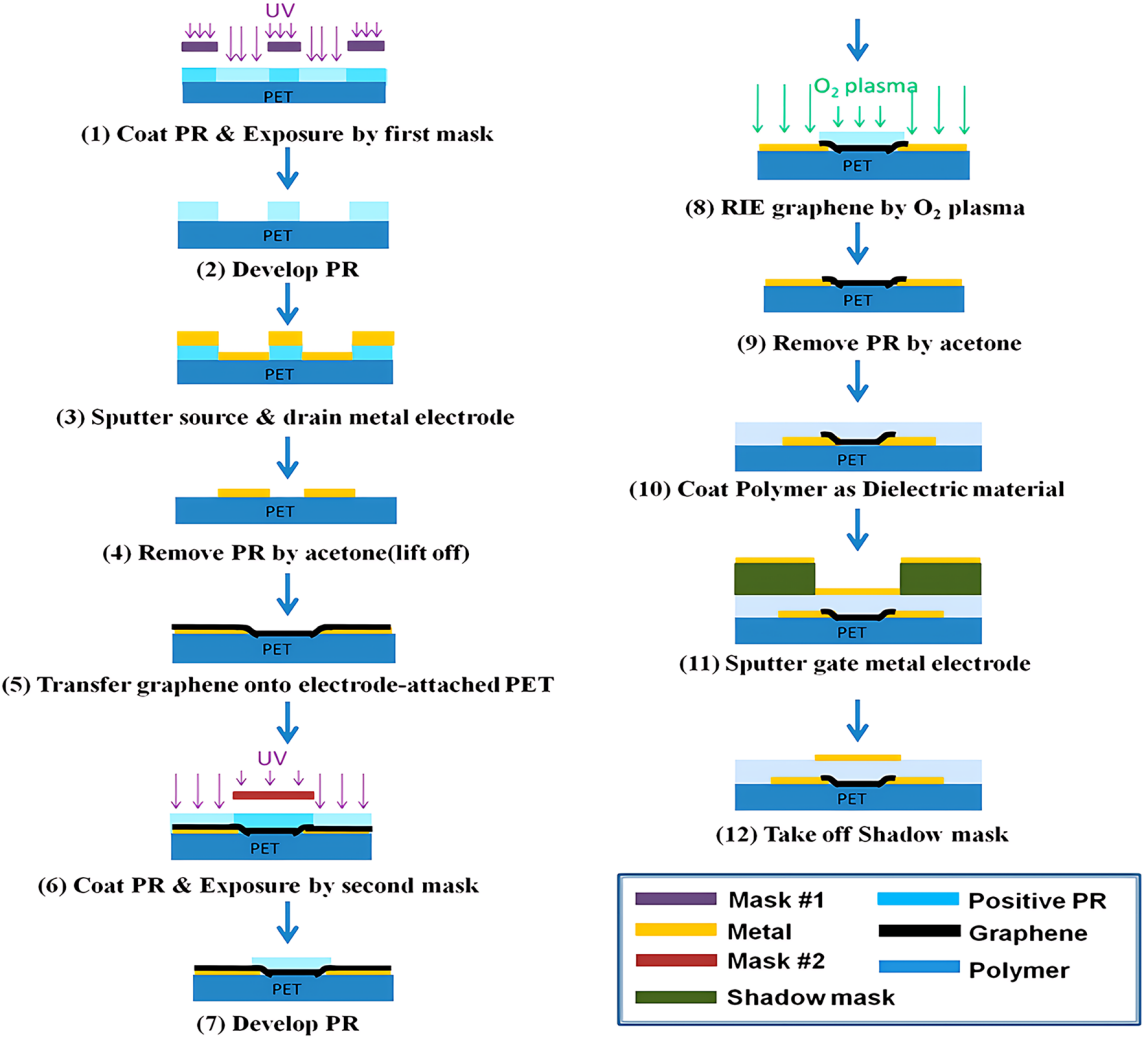
Photolithographic techniques associated with patterning the components of FGFET. Flowchart of the preparation process for flexible transparent GFET using photolithography patterning technique. Reproduced with permission from Ref. [62], Copyright 2014, American Physical Society

Photolithographic process is a method of transferring patterns from photomasks to substrates through a physicochemical process using photoresists with varying light sensitivity under illumination. It is usually patterned on the insulator using photoresist after spin coating of the desired pattern or structure, and further realized by a peeling process (Fig. 8) [200, 201]. Due to its high alignment and nesting accuracy, relatively simple photomask production, and easy-to-understand process conditions, photolithography has become a cornerstone of the development of the microelectronics industry. Generally, all components of a GFET device can be patterned by photolithography. For example, Tsai et al. [62] utilized micromachining of the semiconductor layers and source-drain electrodes of GFETs using photolithography. The flexible transparent Gr-FET showed good carrier mobility with 350 cm^2^ V^–1^ s^–1^, which changed by less than 10% when the bending radius of the flexible device was reduced from 6.0 to 1.0 cm. Zhuang et al. [110] patterned chromium/gold metal electrodes in the conductive channel and gate of the FGFET using standard double-layer lift-off lithography. In addition, Qing et al. [131] developed GFETs on microbial cellulose (MBC) using photolithography to micromachine the semiconductor layers, and dielectric layers of the device. Utilizing the biocompatibility of graphene and the unique biocompatibility of MBCs, their development of implantable sensors and radiofrequency electronics was a major step forward.

Although photolithography enables the patterning of graphene on flexible substrates, the photoresist solvent used during the photolithography process may remain in the polymer and thus degrade the device performance. To avoid this chemical damage, Gao et al. [202] optimized the photolithography process i.e., using a photoresist bilayer (polydimethyl glutarimide (PMGI) protective layer and S1813 imaging layer) to build the GFET. This method eliminates the photoresist on the graphene channels and forms a clean graphene interface, allowing the constructed GFETs to have high carrier mobility (3590 ± 710 cm^2^ V^–1^ s^–1^) and low accidental doping (Dirac voltage of 9.4 ± 2.7 V).

#### Soft Lithography

Soft lithography includes a range of methods that use patterned elastomers as stamps, molds or masks (rather than rigid photomasks) to produce micropatterns and microstructures [203, 204]. Common soft lithography methods include micro-transfer molding (μTM) [205], micromolding in capillary (MIMIC) [206], solvent-assisted micromolding [207] (SAMIM) and microcontact printing (μCP) [208]. In general, soft lithography is suitable for solution technology and thus is mainly used for patterning graphene solution materials. Compared to conventional lithography, soft lithography offers numerous advantages such as affordability, simplification of experiments, and adaptability to various sample geometries, making it an ideal method for fabricating micrometers and nanostructures [204, 209, 210].

**Fig. 9:**
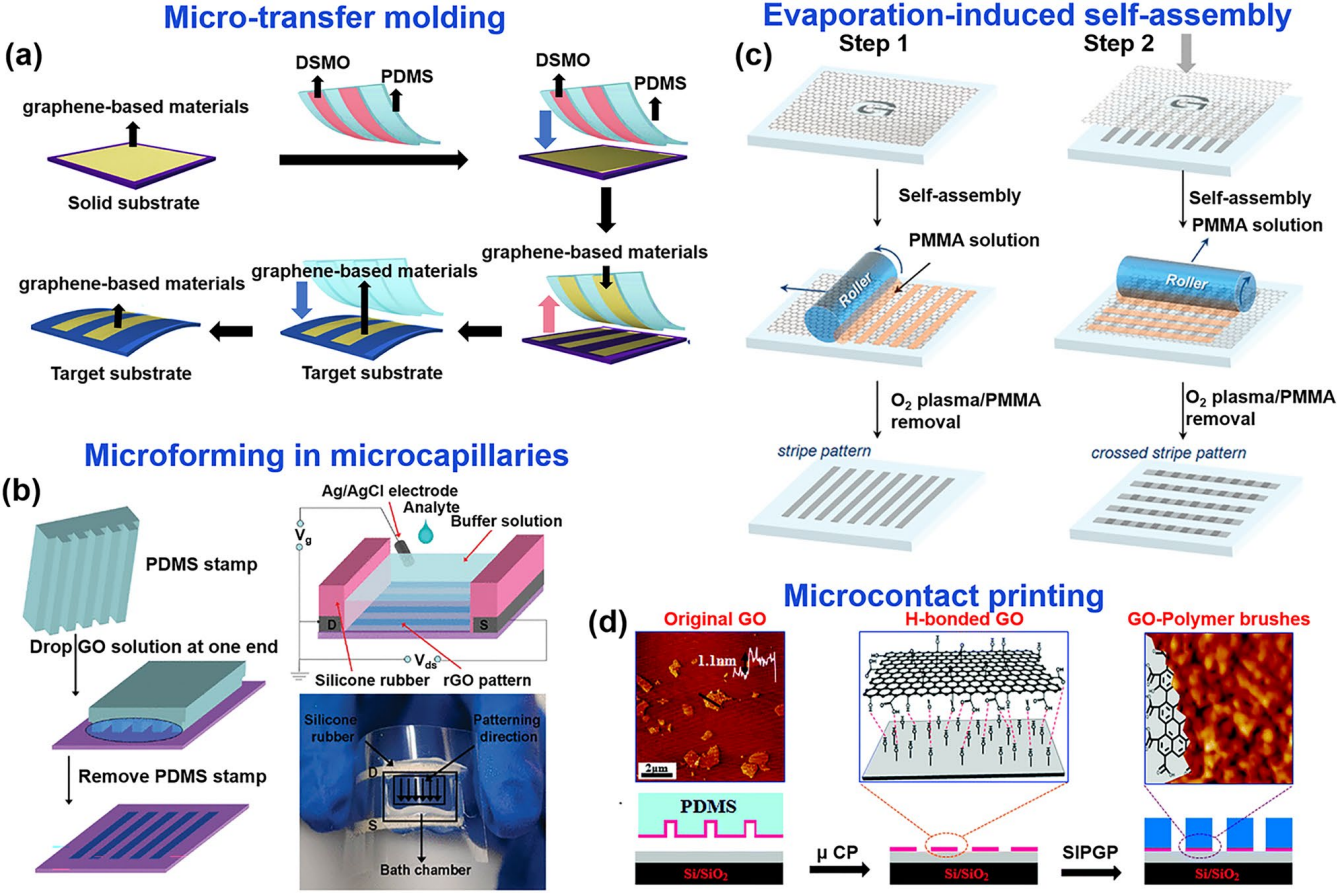
Soft lithography techniques associated with patterning the components of FGFET. **a** Schematic diagram of patterning by the micro-transfer molding (μTM) method. **b** Schematic diagram of fabrication of rGO FETs, in which graphene channel was patterned by the microforming in microcapillaries. Reproduced with permission from Ref. [63], Copyright 2010, American Physical Society. Schematic of graphene patterning by **c** evaporation-induced self-assembly (EISA) method [215] and **d** microcontact printing (μCP) method [216]. Reproduced with permission from Ref. [215], Copyright 2012, American Physical Society, and Ref. [216], Copyright 2014, Royal Society of Chemistry

The micro-transfer molding (μTM) method represents a valuable approach for precisely patterning parallel and isolated microstructures. At present, μTM is widely acknowledged as a straightforward, efficient, and adaptable technique specifically suited for creating graphene patterns (Fig. 9a). In general, this method involves first spin-coating graphene-based materials onto a substrate, then bringing a PDMS mold modified with hydrophilic dimethylsulfoxide (DMSO) into contact with the graphene-based material, transferring the graphene-based material from the initial substrate to the PDMS stamp. Subsequently, the PDMS stamp with the attached graphene-based material is brought into contact with the target substrate. Due to the energy recovery of the PDMS surface, the graphene-based material separates from the PDMS surface, forming a graphene pattern (Fig. 9a). Jung et al. [211] provide a typical example of using this graphene patterning method to manufacture FGFETs. They utilized surface energy modification of hydrophilic coating molecules to transfer GO-palladium nanoparticles (rGO-PdNP) composite material onto silk substrates. This rGO-PdNP material was used as a semiconductor layer to construct FGFETs, and exhibited good mobility (311.37 cm^2^ V^–1^ s^–1^). Based on the ease of manufacturing, biocompatibility, and excellent physical properties of graphene-based materials, graphene material μTM patterning methods have become ideal candidates for advanced flexible devices or bio-related applications.

Micromolding in capillary (MIMIC) is a soft lithography technique that relies on microfluidic systems and is considered a suitable method for fabricating polymer-patterned microstructures on different substrate surfaces [212]. It forms an empty channel grid (PDMS mold) through the contact between the mold and the substrate. The solution then flows into the channels and fills them by capillary forces [213, 214]. The pressure difference between the two hydrodynamic connection regions of the fluid leads to the flow of the material solution in the capillaries. After infusion and material deposition, the PDMS mold can be removed from the substrate (Fig. 9b) [63, 212]. Using this technique, He et al. [63] mapped centimeter-long, micron-wide, ultrathin (1–3 nm) continuous rGO network films on flexible polyethylene terephthalate (PET) films as conducting channels for field-effect tubes. rGO field-effect tubes exhibit significant, bend-insensitive field effects, capable of detecting the presence of biomolecules and dynamic cellular secretion. The ability to fabricate large area ultra-thin rGO films in high yield and reproducibility using the MIMIC patterning technique will open the way for extensive doping of graphene in electronics or bioelectronics.

The spontaneous self-assembly approach is an attractive alternative because it provides a straightforward and economical way to produce highly ordered surface patterns in a scalable manner. Notably, evaporation-induced self-assembly (EISA) has been recognized as an effective bottom-up method capable of producing a wide range of complex structures. Figure 9c shows the patterning of graphene using the EISA method, which is compatible with the roll-to-roll system and allows the pattern to be extended to an arbitrarily large region [215]. By the EISA method, PMMA forms an ordered array on the graphene surface, and the part of graphene not protected by PMMA stripes is removed from the top of the graphene pattern by O_2_ plasma etching to form a graphene pattern with transverse stripes. The graphene longitudinal stripe pattern perpendicular to the transverse stripe is formed by rotating the substrate by 90° in the self-assembly step. Based on the developed graphene patterns, flexible GFET were successfully constructed, demonstrating excellent performance at low voltages of ± 2 V with hole and electron mobility reaching 214 and 106 cm^2^ V^–1^ s^–1^, respectively. This assembly strategy presents a cost-effective and easily scalable approach while also enabling the large-scale production of graphene patterns on flexible substrates. This advancement paves the way for the development of large-area and flexible graphene devices.

μCP is considered one of the most useful fabrication methods for patterning large areas (> cm). Unlike microtransfer techniques that require ink deposition on a rigid substrate, it involves the direct application of self-assembled monolayer molecule (SAM) chemical ink to the surface of a flexible polymer stamp, followed by contact between the stamp and the substrate to form patterns. Peng et al. [216] fabricated GO micropatterns directly on various hydroxylated substrates through μCP-induced supramolecular interactions (Fig. 9d). Due to the formation of multiple hydrogen bonds between GO nanosheets and OH end-surfaces, which resulted in a stronger interaction between GO and silicon, the GO patterns remained stable on the surface even during the ultrasonic treatment cleaning process. This lays the foundation for the preparation of stable graphene patterns.

#### Inkjet and Aerosol Jet Printing

**Fig. 10:**
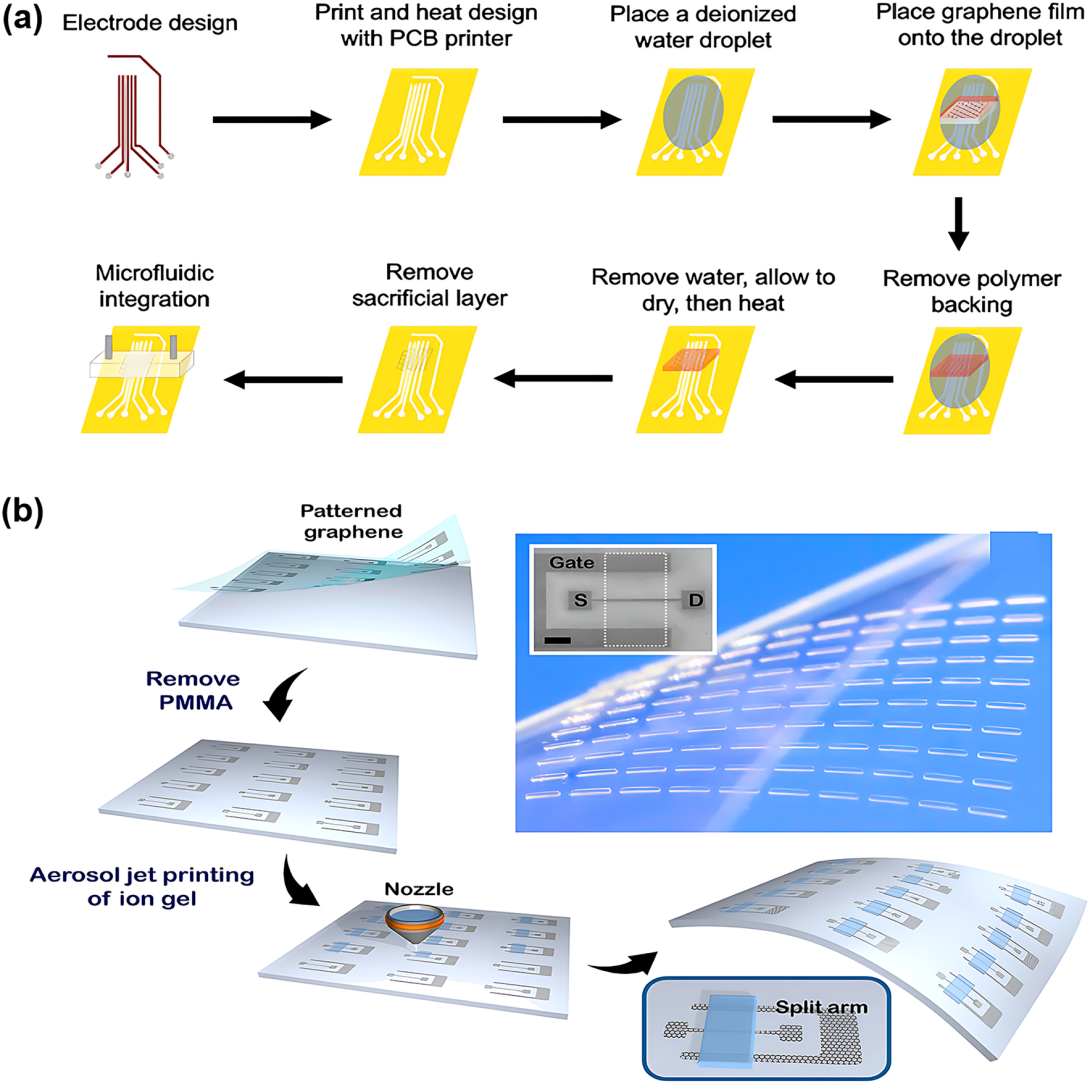
Inkjet and aerosol printing techniques associated with patterning the components of FGFET. **a** Fabrication process of FGFET devices in which the electrodes are patterned by inkjet printing. Reproduced with permission from Ref. [182], Copyright 2022, Elsevier Ltd. **b** Schematic of the fabrication of ion-gel gated graphene transistor arrays on a flexible plastic substrate, where the ion-gel gated dielectric is pattern by aerosol jet printing techniques. Reproduced with permission from Ref. [64], Copyright 2012, American Physical Society

Inkjet printing is a new and rapidly evolving technology. It uses a small particle size micro-scale process for contactless jetting on flexible or rigid substrates for patterning [105]. In inkjet printing technology, several critical parameters, including droplet jet characteristics, solvent evaporation behavior, ink viscosity, and nozzle diameter, significantly influence the resolution. To enhance the quality of inkjet printed films, researchers have made many efforts in ink engineering [217–219], substrate treatment [220–222] and equipment development [223]. These improvements can effectively improve the controllability of the shape, thickness and morphology of the dried droplets, and enhance the resolution of printed films. Inkjet printing has many advantages including precise control of deposition volume and location, ease of patterning, efficient material usage and minimal contamination from non-contact deposition processes, and good biocompatibility, all of which are required for advanced flexible and wearable electronics manufacturing [103]. Laliberte et al. [182] used a conductive ink printer to print source, drain, and gate electrodes on flexible polymer films and then transferred graphene films to form a flexible GFET (Fig. 10a). This GFET modified the aptamer to achieve detection of interleukin-6 (IL-6) protein and exhibited good flexibility. In addition to printed electrodes, inkjet printing techniques have also been used to prepare semiconductor and dielectric layers for FGFETs [101, 224]. A typical example is reported by Xiang et al. [224] All components of the FGFET they built were prepared by inkjet printing.

Aerosol jet printing is a cutting-edge technique that utilizes aerodynamic focusing to enable high-resolution deposition of colloidal suspensions or chemical precursor solutions. This printing method uses ultrasonic vibration or airflow to form an aerosol of nano-conductive ink, which is then transferred using a transfer carrier gas (atomization) to the inkjet port where the conductive ink is printed onto the desired substrate [225]. In aerosol jet printing, the jet consists of numerous droplets, each having a diameter of 2–5 µm, as opposed to inkjet printing, where a single droplet is used. Aerosol printing offers a freer range of height adjustment than inkjet printing that relies on the gravity of the ink droplets to be ejected downward. It depends on the directionality of the masking gas to ensure precise printing, and it is compatible with non-flat substrates [226, 227]. Typically, aerosol jet printing is used to pattern electrodes and semiconductor layers, and the FGFET proposed by Yu et al. is a typical example [228]. The devices were printed a source and drain with a channel width of 200 μm and rGO ink (PBA/rGO) semiconductor layer on a flexible PET substrate by AJP. The Ag/AgCl reference electrode was employed as the gate electrode to provide the desired gate voltage (*V*g), thus constructing a flexible solution-gate GFET. By immobilizing sheep anti-rabbit IgG with AJP, the sensor exhibited good sensing performance. In addition, Kim et al. [64] transferred graphene layers and patterned dielectric layers using an aerosol jet printing technique to prepare transparent flexible graphene transistors and inverters based on coplanar gate configurations (Fig. 10b). Despite the straightforwardness of the production process, these devices demonstrate outstanding mechanical flexibility and environmental stability. Aerosol jet printing will drive graphene FETs toward printability, transparency, flexibility, and large scale.

#### Laser Direct Writing Technology

Laser direct writing (LDW) technology uses a computer to design the patterned pattern and then ablates the laser beam directly onto the substrate surface to complete the pattern transfer [229, 230]. The process eliminates the need for masking and vacuum deposition, and thus has become one of the most powerful patterning strategies due to its fast and simple processing capabilities, high material utilization, nanoscale spatial resolution, and low contamination [231–233].

**Fig. 11:**
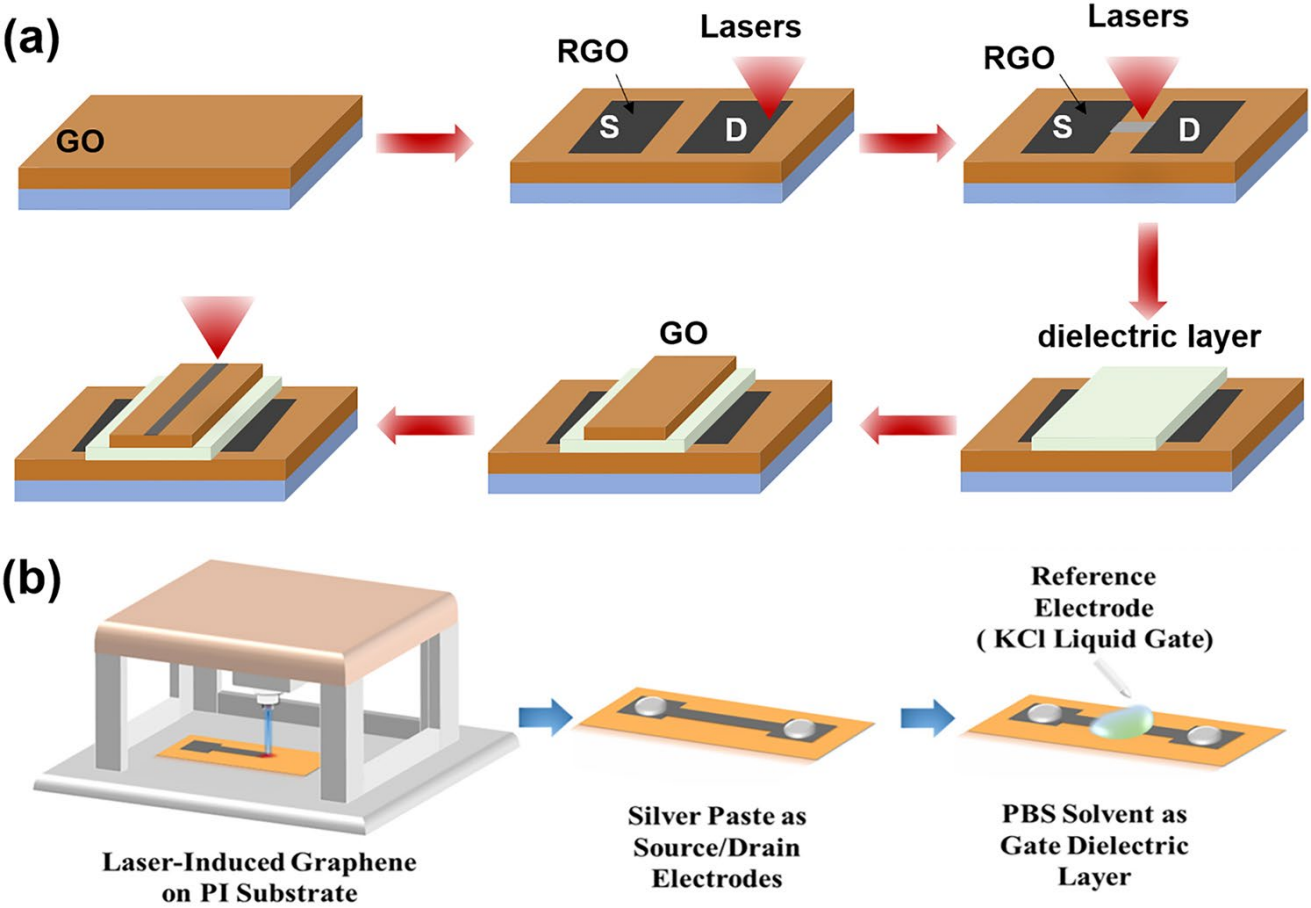
Laser Direct Writing technologies associated with patterning the components of FGFET. **a** Schematic illustration of the experimental procedures for generation of all-RGO FETs based on the FsLDW, in which the metallic and semiconducting RGO micro-patterns could be achieved by FsLDW. **b** Fabrication process of the laser-induced graphene field-effect transistor, in which the source/drain electrodes, as well as semiconducting channel were written directly onto PI substrate by laser. Reproduced with permission from Ref. [59], Copyright 2019, WILEY–VCH

Ultrafast laser-based graphene processing has yielded impressive results, opening up a wide range of subtractive and additive processes for patterning and functionalizing atomic layer materials. Among them, femtosecond laser processing has attracted attention for its valuable attributes, including scalability, heat-affected zone reduction, and mask-free, programmable patterning capabilities [230, 232, 233]. Femtosecond laser pulses are capable of locally patterning and tuning the electrical properties of graphene oxide (and ultimately transforming it into reduced graphene oxide) in a controlled manner, and are therefore widely used for GO electrodes and patterning of semiconductor layers (Fig. 11a) [234]. For example, He et al. [235] employed an innovative femtosecond laser direct writing (FsLDW) technique to effortlessly produce fully reduced graphene oxide field-effect transistors on flexible substrates by precisely controlling the intensity of the femtosecond laser pulses. At high power and medium power lasers, the FsLDW enables metal and semiconductor RGO micropatterning, respectively, so that source/drain and gate electrodes, as well as semiconductor channels of field effect transistors, can be written directly onto the flexible substrate under ambient conditions. The device shows an *I*_on_/*I*_off_ of 2.04 and an estimated hole mobility of 0.27 cm^2^ V^−1^ s^−1^. FsLDW advances the application of graphene in transparent, flexible, and stretchable electronic devices, while also facilitating the development of FGFETs. Significantly, the fabrication of metal-free all-RGO FETs through FsLDW eliminates the need for masks or chemical reagents. This method simplifies the preparation process and allows for the flexible integration of graphene-based microdevices, showcasing its significant potential for advancing future electronic devices. FsLDW for all-RGO devices offers distinct advantages, making it a promising avenue for realizing the next generation of electronic devices.

Laser-induced graphene (LIG) is a special type of LDW, a technique that forms stacked graphene structures in a photothermal reaction by irradiating PI and PEI films with a pulsed CO_2_ infrared laser [236]. LIG provides favorable electrical properties and the ability to simultaneously fabricate patterns, which makes laser-induced technology an effective method for fabricating graphene-based devices. In addition, the low cost, high fabrication density and the ability to integrate laser-induced graphene directly into flexible common polymers have led to the widespread interest in this method in the field of flexible electronics and its application to the preparation of FGFETs [237]. For example, Cui et al. [238] used a laser platform by tuning the characteristics of the laser to obtain laser-induced graphene with different reduction levels (Fig. 11b). The FGFETs were constructed using higher reduction LIG as the source-drain electrode and lower reduction LIG as the semiconductor layer. Individual LIG-FETs can be fabricated by the laser platform in seconds, and LIG-GFET arrays can be constructed in minutes. This device construction method patterns semiconductor layers and electrodes while preparing them, providing a good solution for low-cost, simple processes and large-scale fabrication of FGFETs.

In summary, as outlined in Table 1, photolithography offers high resolution, capable of achieving micrometer- and even nanometer-scale patterns. Its reproducibility, suitability for large-scale and batch production, and process maturity make it ideal for fabricating high-precision GFETs on rigid substrates. However, the complexity of the process and the high cost of equipment limit its application on flexible substrates, restricting its use primarily to laboratory research and small-scale production of FGFETs. In contrast, soft lithography is characterized by lower cost and a relatively simpler process. Its flexibility makes it suitable for fabricating GFETs on large-area flexible substrates, particularly for low-cost biosensors and wearable devices. However, the lower resolution and pattern consistency of soft lithography render it less suitable for applications requiring high precision. Inkjet and aerosol jet printing techniques offer a non-contact printing process that simplifies production, reduces costs, and provides high flexibility to print complex and diverse patterns, making them well-suited for customized FGFET production. However, these technologies have relatively low resolution and demand precise ink formulation and printing accuracy, limiting their use mainly to sensors, displays, and flexible electronic circuits. On the other hand, laser direct writing technology is capable of producing high-resolution, high-precision patterns without the need for a mask. This technique allows direct pattern writing onto materials, making it ideal for manufacturing flexible GFETs with complex designs. Despite its high equipment costs and slower processing speeds, laser direct writing offers unique advantages in producing high-precision, complex patterns and nanostructures, making it suitable for microelectronic devices and high-end flexible electronics. The integrated application of these technologies effectively addresses the growing market demand for flexible electronic devices and fosters the advancement of the FGFET field and related industries.

**Table 1:** Comparison of FGFET patterning processes

Patterning processes	Advantages	Disadvantages	Application scope
Photolithographic	High resolution, capable of sub-micron features Mature process, good repeatability Suitable for large-scale production	Complex process with many steps Requires high-cost equipment and cleanroom environment Difficult to process on flexible substrates	Mainly used for high-precision patterns on rigid substrates in GFET fabrication Suitable for laboratory research and small-scale production of FGFETs
Soft lithography	Low cost, requires less equipment Relatively simple process Suitable for large-area production Ideal for flexible substrates	Relatively low resolution Poor pattern consistency Limited applicability for high-repeatability needs	Suitable for large-area flexible GFET manufacturing Applicable in biosensors and wearable devices
Inkjet and aerosol jet printing	Enables non-contact printing Flexible process, capable of printing a wide range of materials Low cost Suitable for large-area production Ideal for flexible substrates	Relatively low resolution High requirements for ink formulation High demands on printing accuracy and uniformity	Suitable for low-cost, large-area, customizable flexible GFET manufacturing Applicable in sensors, displays, and flexible electronic circuits
Laser direct writing technology	High resolution, suitable for complex patterns Non-contact processing Ideal for flexible substrates Flexible process	High equipment cost Relatively slow processing speed	Suitable for high-precision, complex patterns in flexible GFET manufacturing Applicable in microelectronics and nanostructure fabrication

### Effect of Strain on the Electrical Properties of FGFETs

For conventional FGFETs, the essence of the stress-induced electrical change is the change in graphene structure due to strain on the graphene conducting channel. Strain modifies the lattice structure of graphene, influencing its electrical transport properties and leading to significant changes in electrical coupling [239–241]. Theoretical calculations indicate that both symmetric and asymmetric strains can dramatically alter the width of the pseudo-bandgap near the Fermi level [242, 243]. Specifically, tensile strain reduces the pseudo-bandgap, while compressive strain increases it. A reduction in the pseudo-bandgap decreases the electronic density of states, making it more challenging for electrons to transition between the conduction and valence bands, thereby increasing graphene’s resistance. In contrast, compressive strain enhances the electronic density of states, which leads to a decrease in resistance. Therefore, graphene with a perfect lattice exhibits a piezoresistive effect. Additionally, asymmetric strain disrupts the symmetry of graphene and can open a bandgap, with the magnitude of this effect being directly related to the direction of the applied strain. For example, when the strain direction is parallel to the C–C bond, a strain of 12.2% causes the gap to widen by 0.486 eV. When the strain direction is perpendicular to the C–C bond, a strain of 7.3% causes the gap to widen by 0.170 eV [242]. The continued increase in strain eventually leads to C–C bond breakage and graphene film destruction. Ni et al. [244] simulated the fracture behavior of graphene by molecular dynamics, when the strain direction is parallel to the C–C bond, graphene cracks at ~ 30% of the strain; if the strain direction is perpendicular to the C–C bond, the fracture strain increases to ~ 40%.

**Fig. 12:**
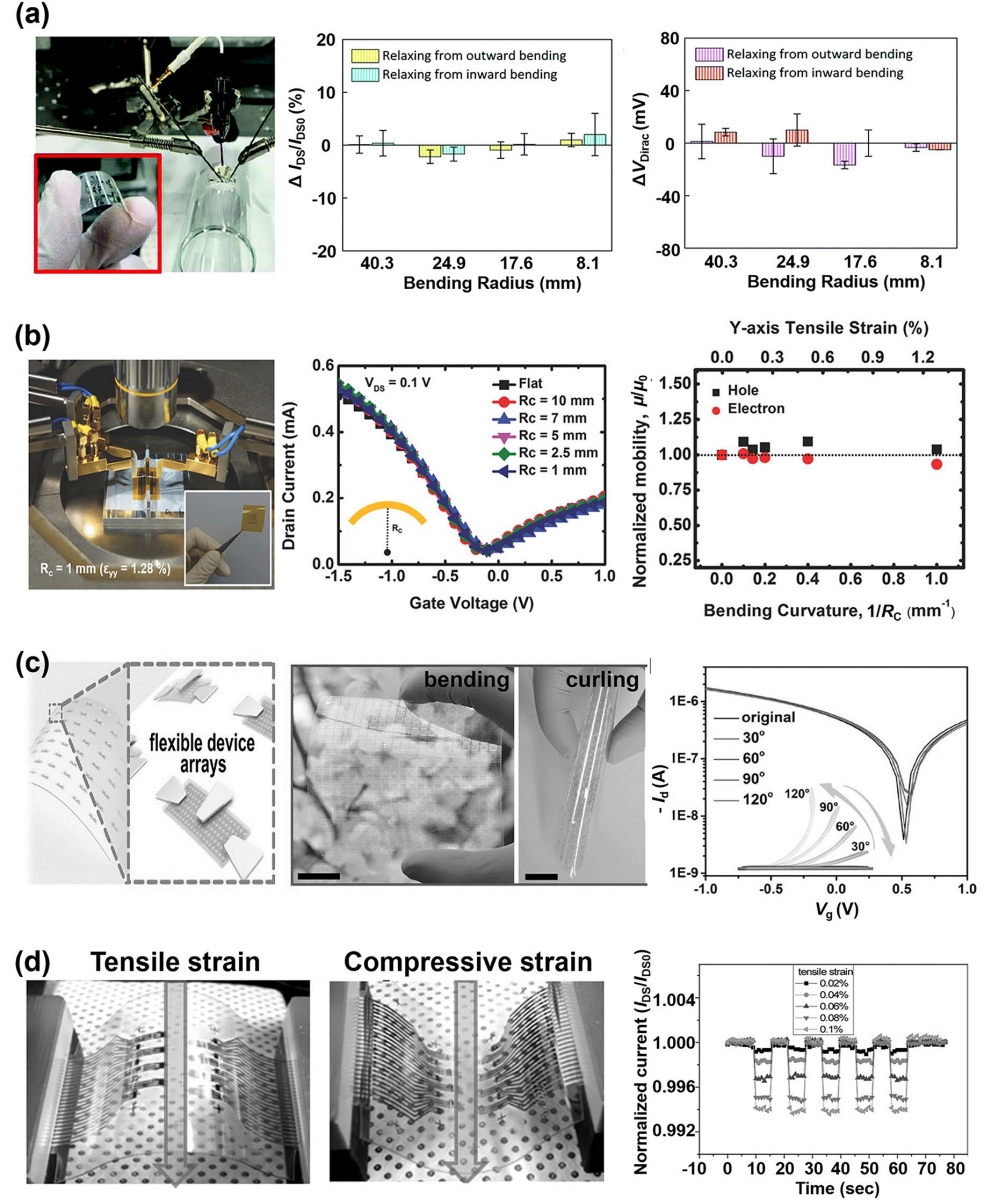
Effect of strain on the electrical properties of flexible graphene FETs. **a** Flexible nanosensor placed on a nonplanar surface. Changes in the resistance, the Dirac point and the transconductance of graphene after recovery (for 15 min) from bending. Reproduced with permission from Ref. [245], Copyright 2018, RSC Publishing. **b** Image of electrical measurement setup for the flexible top-gated graphene FETs. Charge transfer characteristics of graphene FETs flexed at different bending radius. Normalized hole and electron mobility values of graphene FETs flexed at different bending curvature and repeated cycles of bending stress. Reproduced with permission from Ref. [145], Copyright 2018, WILEY–VCH. **c** Structural models of GNM FET device arrays on flexible PET substrate.* I*_d_-*V*_g_ at bending angle of 30°, 60°, 90°, and 120°, respectively. Optical images of the transparent and flexible GNM FET device arrays under bending, curling, and twisting conditions, respectively. Reproduced with permission from Ref. [246], Copyright 2017, WILEY–VCH. **d** Photographs of the device array under tensile and compressive strain loads. Time dependence of the normalized current *I*_DS_/I_DS0_ under very small applied tensile and compressive strains. Reproduced with permission from Ref. [92], Copyright 2014, WILEY–VCH

However, based on the limitations of the preparation method, true graphene is often not an ideal single crystal, but has a polycrystalline structure containing grain boundaries with various internal defects (excess or missing carbon atoms in the carbon six-membered ring, oxygen-containing functional groups). These grain boundaries and defects will inevitably affect the electronic structure of graphene, making the electrical characteristics of FGFETs different during strain [139, 145]. For example, Zhuang et al. [245] utilized CVD graphene to prepare field effect transistor nanosensors. When the bending radius is reduced from planar to 8.1 mm, the graphene resistance and the Dirac point of the transfer curve undergo significant unidirectional changes in terms of increase and negative shift, respectively. Upon relaxation from the bent state, both the original resistance and the Dirac point are also nearly restored, while the transconductance, which is an important marker of the nanosensor's sensitivity, remains nearly constant at all times (Fig. 12a). Joong Gun Oh et al. [145] also used CVD graphene to prepare GFET sensors. The 1-vinylimidazole (VIDZ) monomer with imidazole portion was doped into pV3D3 to form a homogeneous copolymer (p(V3D3-*co*-VIDZ)) thin film as a dielectric layer by an iCVD process, which suppressed the unintentional p doping in graphene and improved the device performance. The Dirac point and transconductance of the sensor were unaffected by the increase in tensile strain from 0 to 1.28%, and the carrier mobility changed by less than 9%. The sensor was subjected to 1000 reciprocal bending tests with a bending radius of five millimeters and the change in carrier mobility was less than 8% (Fig. 12b). Due to its zero-bandgap structure, graphene has a low switching ratio, which limits its utility in digital/logic applications. Yang et al. [246] transferred large-area graphene grown by CVD onto poly(ethylene terephthalate) (PET) substrates and prepared ultrathin graphene nanomesh (GNM) with considerably narrower neck width (< 3 nm) and stronger quantum confinement by punching holes in the graphene layer using plasma etching with directly grown mesoporous silica as a template. GNM-based field effect transistors achieve high on/off ratios (up to 1000) and large surface/volume ratios. The on/off ratio decreases only slightly as the bending angle increases from 30° to 120°, and the conformal contact is maintained during bending, curling, and twisting, indicating that the GNM-based field-effect transistor exhibits excellent bending durability throughout the bending process (Fig. 12c).

Unlike the strain stability of CVD graphene, the residual oxygen functional groups and inherent structural defects in the rGO nanosheets as well as the disorder caused by the coupling between neighboring nanosheets result in a strong modulation of the electrical resistance or conductivity of their films under strain [92, 247]. The rGO FET strain transducer exhibits ultra-sensitivity in very low strain modes and is capable of detecting tensile and compressive strains as small as 0.02% (Fig. 12d) and its ability to respond and relax dynamic tensile and compressive strains very quickly [92]. Moreover, the addition of appropriate dopants such as Al, B, N, S, and Si to graphene can also alter the electronic properties of graphene under strain, improve the sensitivity of graphene to strain, and increase its potential for applications in strain sensing [248].

In summary, the choice of graphene raw materials, manufacturing processes, and transfer methods plays a critical role in determining the number of graphene layers, lateral dimensions, internal defects, grain boundaries, and interactions with the substrate when designing and deploying FGFETs. These factors ultimately dictate the strain sensitivity of FGFETs and their suitability for various applications. In general, grain boundaries and defects can affect the transport gap in graphene, thereby enhancing its sensitivity to strain [249–251]. Consequently, CVD graphene with fewer grain boundaries and defects exhibits lower strain sensitivity but demonstrates high responsiveness to charged biomolecules. This makes it particularly advantageous for developing highly stable and flexible biomedical sensors. In contrast, rGO, due to its inherent structural defects and the presence of oxygen functional groups, is more sensitive to strain, making it ideal for high-sensitivity applications such as pressure sensors and deformation detection. Furthermore, the strain sensitivity of graphene can be enhanced through doping and chemical modifications, broadening its applications in advanced sensors and wearable devices such as electronic skin and various activity monitors. The versatility and adaptability of FGFETs allow them to meet a wide range of demands, from high stability to high sensitivity. However, achieving this requires careful design and the selection of appropriate graphene materials and processing methods.

## Applications of FGFET in Flexible Biomedical Sensors

On the one hand, the unique three-terminal structure, signal amplification, and small size of field-effect transistors enable them to capture small charge changes while adjusting any component parameter. They are also easy to integrate into electronic fabrication processes for "lab-on-a-chip" functionality. On the other hand, graphene's excellent electrical, biocompatibility, and mechanical properties make GFETs not only capable of maintaining the biological activity of biomolecules and sensitively responding to changes in them, but also ideally suited for applications that require contact with soft or curved biological interfaces. Thus, FGFETs inherit the advantages of both graphene and field-effect transistors and offer significant benefits as flexible biomedical sensors. These benefits include ease of operation, fast response time, real-time monitoring capability, high specificity and sensitivity, low detection limits down to femtomole and sub-femtomole ranges, and microfluidic integration, as well as being suitable for wearable and implantable applications. Currently, FGFETs have been integrated into wearable and implantable sensors, which are widely used in various biomedical assays for detecting physiological signals and biochemical parameters.

### FGFET-Based Wearable Biomedical Sensors for Physiological Signals Sensing

With the rapid advancement of smart wearable electronic devices, wearable biosensors have emerged as a promising technology aimed at continuous, real-time, and non-invasive monitoring of physiological indicators [252, 253]. Currently, FGFET-based biosensors are designed to be attached to flexible substrates, allowing them to be easily attached to skin or clothing, enabling the detection of physiological signals (signals generated by movement and contact as well as body temperature) to provide information about the body and its interaction with the environment [254–256].

#### Biomechanical Signals Monitoring

Biomechanical signals are generated by the movement, deformation and mechanical interactions of an organism and play an important role in the organism by providing critical physiological information that helps to regulate movement, control posture, maintain balance and sense the external environment. Monitoring biomechanical signals is crucial not only in sports, rehabilitation, and medicine but also in smart device interaction, virtual reality, and ergonomics. By accurately capturing and analyzing these signals, we can better understand and improve human health and behavior. Therefore, FGFETs are widely used to monitor biomechanical signals such as pressure, touch stimulation, joint motion, heartbeat, and pulse.

**Fig. 13:**
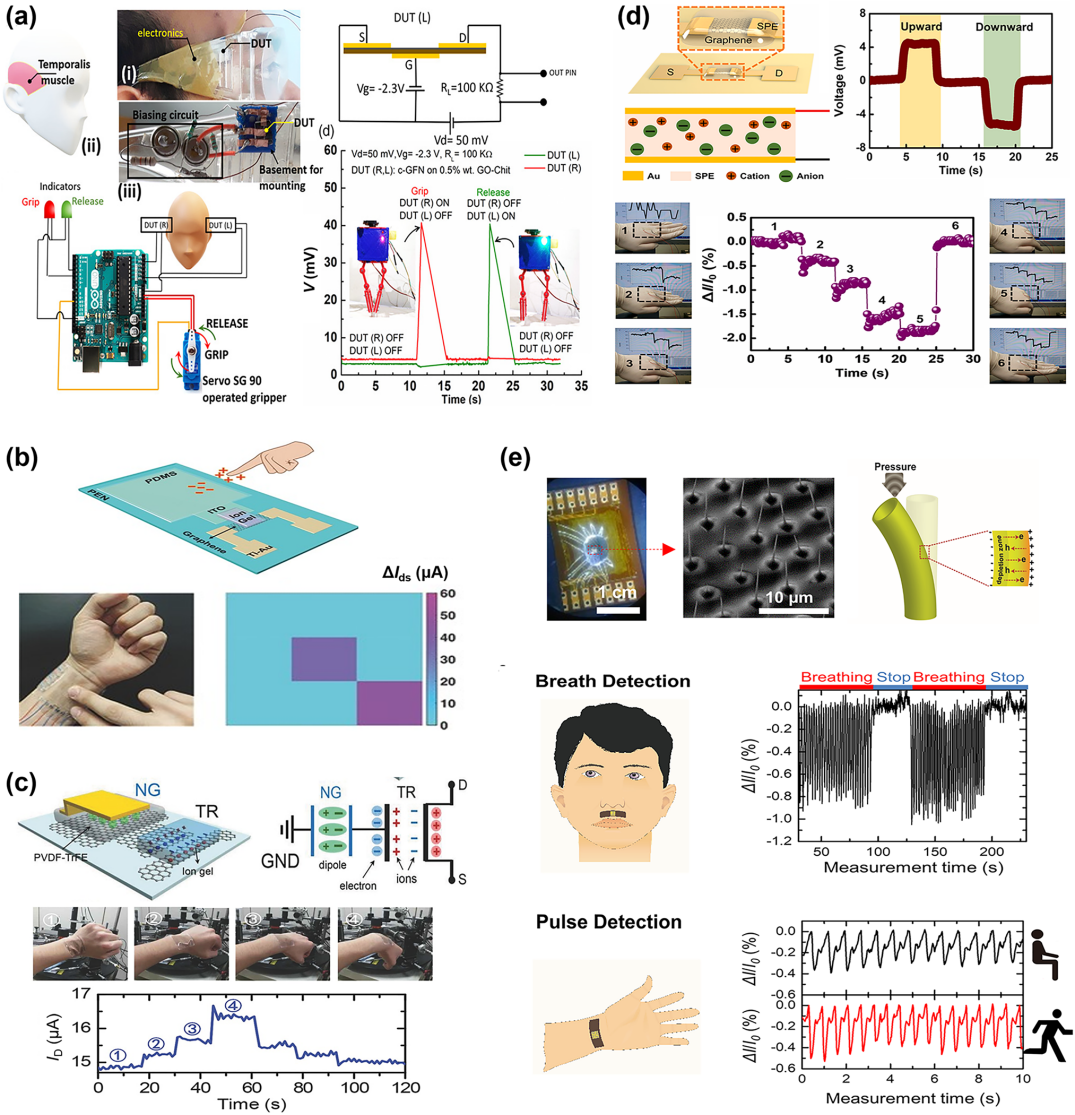
FGFET-based wearable biomedical sensors for biomechanical signals monitoring. **a** The PRESSFET was attached to the inside of a pair of glasses, keeping them in contact with the temple area of the face, to demonstrate hands-free control of the robotic device. Reproduced with permission from Ref. [67], Copyright 2022, WILEY–VCH. **b** Tribotronic touch sensor based on coplanar coupling of a single-electrode-mode triboelectric nanogenerator (S-TENG) and a graphene FET. The devices can effectively detect touch stimuli from both bare and gloved fingers. Reproduced with permission from Ref. [162], Copyright 2017, WILEY–VCH. **c** Hand movement monitoring by a conformal piezopotential-gated GT strain sensor fabricated on a PDMS substrate. Reproduced with permission from Ref. [65], Copyright 2015, Elsevier Ltd. **d** Detection of the joint movement using the SPE-coated graphene field-effect transistor strain sensor. Reproduced with permission from Ref. [261], Copyright 2021, Elsevier Ltd. **e** Pressure sensor directly attached to the philtrum and the wrist of a volunteer for breath detection and pulse monitoring, respectively. Reproduced with permission from Ref. [262], Copyright 2021, Springer Nature

The piezoresistive effect is the most fundamental mechanism for detecting biomechanical signals based on FGFET biomedical sensors. For example, Sahatiya et al. developed a flexible all-paper-based few-layer graphene (Gr)-MoS_2_ FET [130]. When strain is applied, the Gr/MoS_2_ channel deforms under force, leading to resistive modulation of the channel and thus achieving a sensitive response to tensile strain. The integration of graphene not only improves the mobility of this FET but also forms a potential barrier with MoS_2_, increasing sensitivity to small strains. This sensor, integrated into a human hand, enables wireless monitoring of corresponding hand movements via a smartphone, with applications in wireless wearable electronics, security, and human motion monitoring. Additionally, Paul et al. developed a pressure sensing field effect transistor (PRESSFET) used as a wearable switch based on a crumpled graphene flakes network (c-GFN). The unique crumpled morphology of the GFN results in a bandgap of 800 meV, enabling the device to have well-defined ON and OFF electronic states as well as a low subthreshold swing. The device is activated by temporal muscles in the temple region of the face, allowing for hands-free control of robotic platforms (Fig. 13a). The ultra-thin and skin-friendly PRESSFETs are also attractive for wearable applications and are expected to benefit amputees and hemiplegics who have partially or completely lost the ability to control their limbs autonomously.

Although some FGFETs can be strain-sensitive by optimizing the material design of the semiconductor layer, most of the graphene produced by CVD has fewer grain boundaries and defects, making it insensitive to strain. To use FGFETs for detecting biomechanical signals, it is usually necessary to couple components that are sensitive to strain changes. For example, Khan et al. [162] demonstrated a graphene-based friction electronic touch sensor designed to sense touch stimuli. They utilized a single-electrode mode triboelectric nanogenerator (S-TENG) and coplanar coupling of graphene with co-local coupling of graphene field-effect transistors. When any object (e.g., a human finger) contacts the friction layer of the S-TENG, an electrical charge is generated due to the triboelectric effect [257–259]. These charges can be used as the gate bias of the graphene field-effect transistor, regulating its current transfer and effectively detecting touch stimuli from bare and gloved fingers and spatially mapping sphere motion and touch stimuli from multiple fingers (Fig. 13b). The application of piezoelectric sensor matrices can reduce system size and weight and provide advanced sensing characteristics including low signal interference, monitoring of multiple parameters, high sensitivity, and high spatial resolution. Therefore, Sun et al. [65] fabricated a piezoelectric-powered active matrix strain sensor array using a combination of a P(VDF-TrFE)-based piezoelectric nanogenerator (NG) and coplanar-gate graphene transistors (GT). In this sensing system, the piezoelectric potential of the NG under externally applied strain is coupled to the graphene channel via an ion-gel dielectric, enabling effective modulation of the channel conductance of the GT. The resulting strain sensor exhibits excellent sensitivity and stability for continuous monitoring of hand movements. Transparent conformal strain sensors fabricated on a rubber substrate were mounted on the hand of a human tester for continuous monitoring of hand movements (Fig. 13c).

Nevertheless, adding additional piezoelectric or friction electrostatic nanogenerators would undoubtedly increase the complexity and fabrication difficulty of the device [260]. Liu et al. [261] demonstrated piezoelectrically driven strain and touch sensors using mechanically doped graphene and solid polymer electrolytes (SPE). Due to the piezoelectric effect in the SPE, the piezoelectric potential modulates the carrier concentration in the graphene channel when strain is applied. As a result, the Dirac point voltage of the SPE-coated graphene FET (S-GFET) is displaced, and this new doping technique is able to tune the pristine graphene to either an n-type doped state or a p-type doped state for an efficient response to strain. The GFETs exhibit stable output signals at continuous strains and can differentiate between tension and compression without the need for extra components. The strain sensor attached to the hand responds efficiently to hand joint movements and has potential applications in long-term rehabilitation (Fig. 13d). Additionally, the device is well-suited for touch sensing, as the friction potential modulates the current transfer in the S-GFET due to the charged contact between the SPE and an external object. Similarly, Park et al. [262] reported a GFET wearable piezoelectric pressure sensor fabricated using vertically aligned, positionally, and dimensionally controllable arrays of ZnO nanotubes grown on a graphene layer. Due to the piezoelectric properties of ZnO, under increasing applied pressure, the piezoelectric field formed across the width of the ZnO nanotubes traps charge carriers and creates charge depletion zones on the curved outer and inner surfaces of the nanotubes, resulting in a systematic decrease in current. The wearable sensor shows high-pressure sensitivity (− 4.4 kPa^−1^) and can detect weak inert gas flows. Connecting the sensor to the volunteer’s philtrum and wrist allowed for the detection of respiration and heart rate (Fig. 13e). The prototype of the hybrid sensing device holds significant promise as a wearable technology, particularly in the realm of sophisticated healthcare devices.

#### Body Temperature Monitoring

**Fig. 14:**
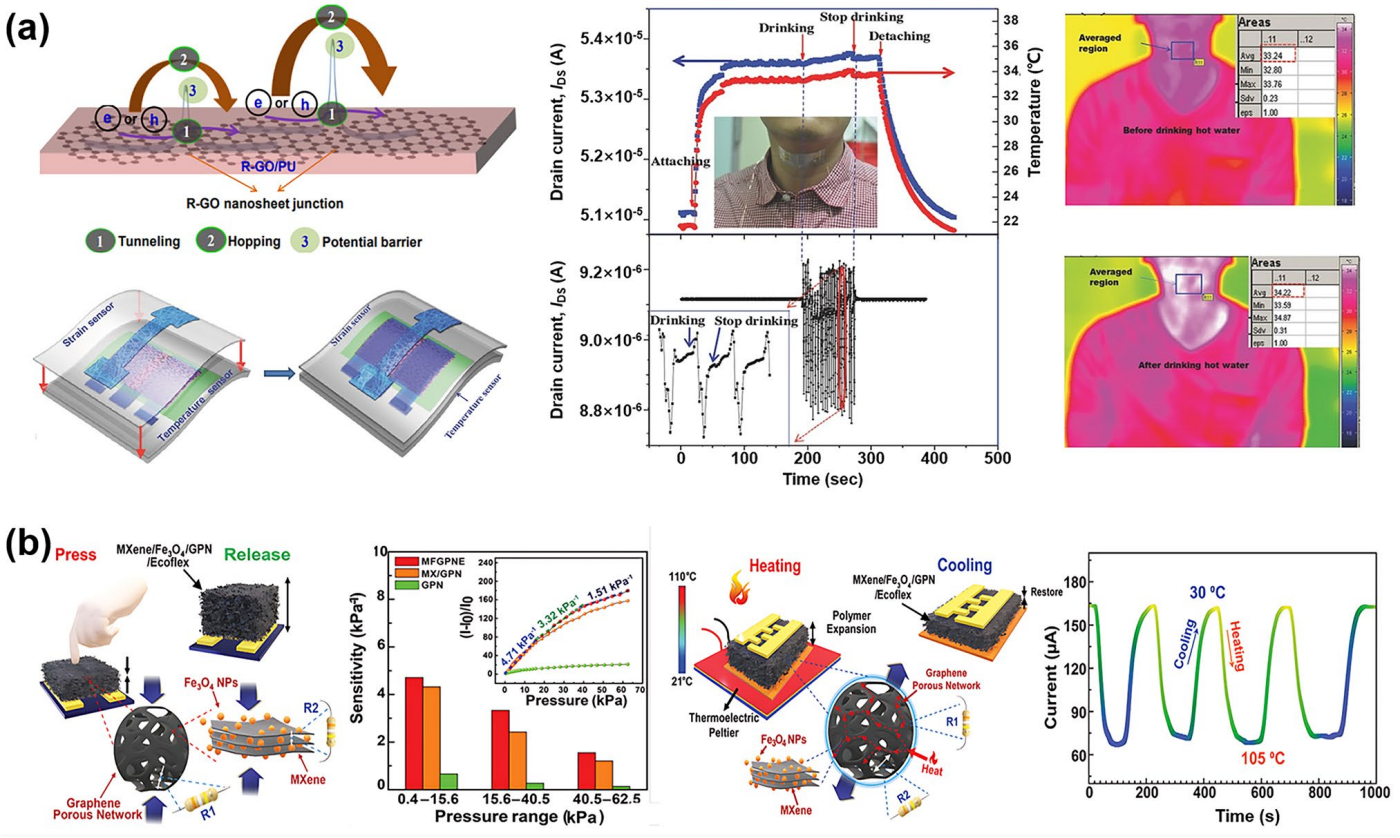
FGFET-based wearable biomedical sensors for body temperature monitoring. **a** Schematic diagram of the sensing mechanism of the R-GO/PU temperature sensor. Electrical and infrared thermography of the temperature sensor integrated with the pressure sensor to simultaneously monitor neck skin temperature and muscle movement. Reproduced with permission from Ref. [66], Copyright 2016, Wiley–VCH. **b** Sensing mechanism and detection performance of the flexible MFGPNE-FET multifunction pressure–temperature sensor. Reproduced with permission from Ref. [264], Copyright 2021, Wiley–VCH

Monitoring body temperature is critical for detecting and controlling diseases, assessing the effectiveness of treatments, and maintaining overall health and well-being [263]. There have been considerable advancements in the creation of GFET temperature sensors. For instance, Trung et al. [133] developed transparent, flexible nanocomposite FETs for temperature monitoring using R-GO/P (VDF-TrFE) nanocomposites. The thermal sensing mechanism of this transparent R-GO/P (VDF-TrFE) nanocomposite FET is explained by hopping and tunneling transport within the material. During hopping transport, the field-effect mobility increases with rising temperature. Consequently, these transparent, flexible nanocomposite FETs are extremely sensitive and can detect temperature changes as small as 0.1 °C, making them ideal for monitoring human body temperature. To facilitate attachment to human skin as a patch, the group subsequently developed a fully flexible FET temperature sensor based on reduced graphene oxide/elastic polyurethane (R-GO/PU) nanocomposites [66]. This device can detect temperature-induced potential changes in the vicinity of the rGO channel region and exhibits an almost linear response to temperature variations. To mimic the versatility of human skin, the researchers also fabricated an integrated platform of transparent, stretchable temperature and strain sensors in a vertically integrated scheme. This setup, affixed to the neck and arm, concurrently tracks minor temperature fluctuations and muscle motions during human activities (Fig. 14a). In addition, Mondal et al. [264] fabricated a pressure–temperature multifunctional sensor using MXene/Fe_3_O_4_/graphene porous network/Ecoflex (MFGPNE) FETs (Fig. 14b). Based on the piezoresistive effect, the sensor responds sensitively to pressure. The different coefficients of thermal expansion of MXene, graphene, and Ecoflex affect the electrical characteristics of the connected networks during heating or cooling, resulting in a linear temperature response of the sensor. By integrating these two functions, the sensor is expected to excel in wearable sensing devices, enabling simultaneous detection of body temperature and pressure.

### FGFET-Based Wearable Biomedical Sensors for Biochemical Molecules Sensing

Wearable sensors designed for biochemical parameter monitoring primarily focus on the dynamic tracking of metabolites in various biological fluids such as sweat [265–268], tear fluid [269], and tissue fluids (ISF) [270]. In contrast to wearable sensors used for monitoring physiological signals, these biochemical parameter monitoring sensors offer a broader perspective on an individual’s health status, providing valuable and dynamic molecular-level information [253, 271, 272]. FGFET-based wearable sensors for biochemical parameter detection have been widely used in the detection of biomolecules such as large biomolecules, nucleic acids and other molecules as well as metal ions.

#### Large Biomolecules Detection

##### Protein Detection

**Fig. 15:**
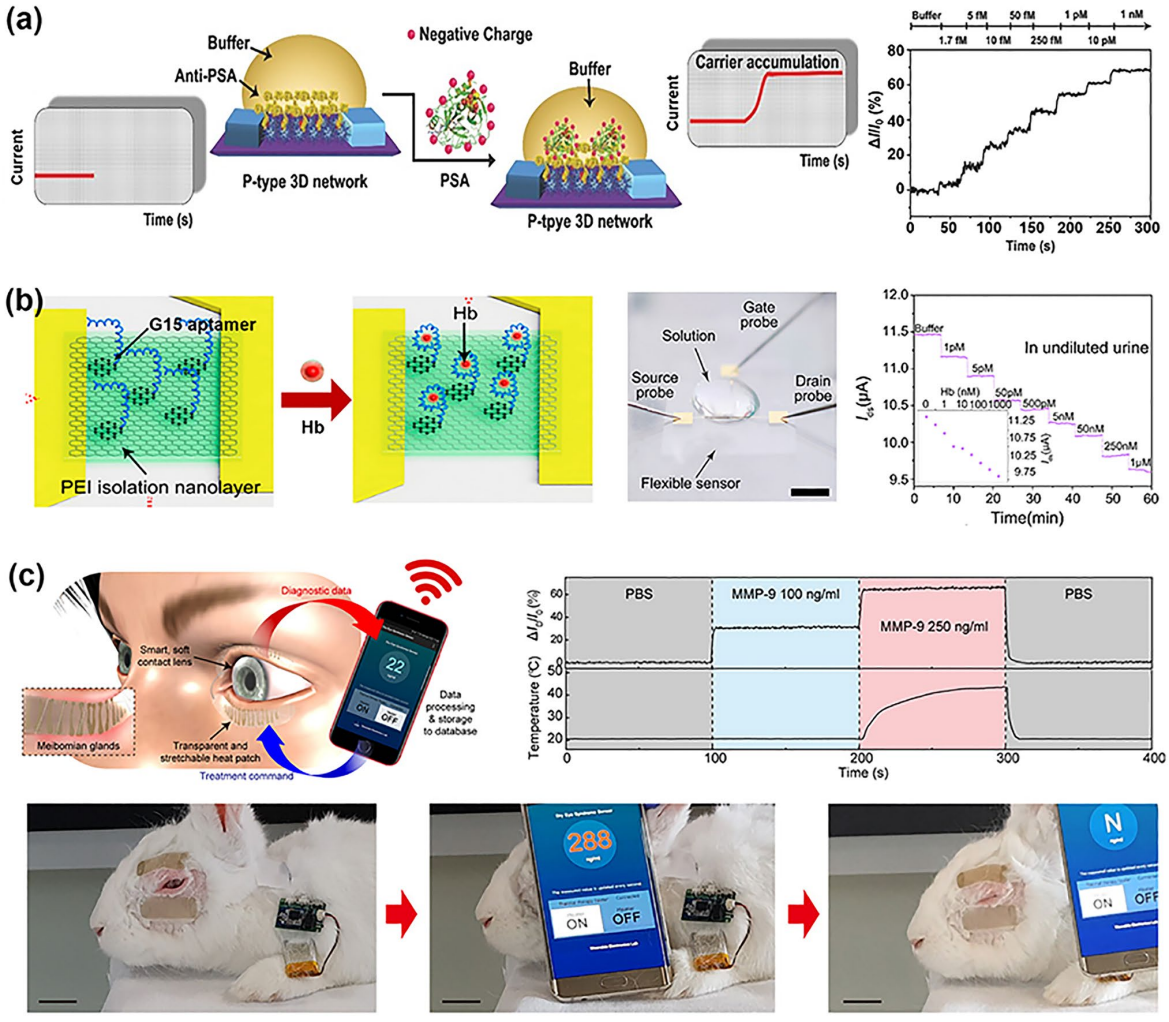
FGFET-based wearable biomedical sensors for protein detection. **a** Sensing mechanism of rGO@SFP FET protein biosensor and its sensing performance. Reproduced with permission from Ref. [274], Copyright 2016, WILEY–VCH. **b** Sensing mechanism of graphene-based aptamer nanobiosensor and its electrical performance in detecting Hb in undiluted urine after various deformations. Reproduced with permission from Ref. [110], Copyright 2022, American Chemical Society. **c** Graphene-FET-based contact lens for remote monitoring and treatment of chronic OSI. Reproduced with permission from Ref. [69], Copyright 2021, American Association for the Advancement of Science

With the emergence of diseases, components such as enzymes, proteins, and antigens that are absent in normal tissues begin to proliferate. Consequently, protein-based targets have become the most commonly used means for accurate disease diagnosis and are widely applied in diagnosing various conditions [273]. To facilitate disease prevention and diagnosis, protein sensors based on FGFETs have been developed, laying the groundwork for wearable sensors. Typically, common FGFET protein assays rely on specific recognition between antigens and antibodies. For instance, Wang et al. [274] developed a flexible biosensor based on an antibody-antigen recognition system, assembled from sunflower pollen (SFP) particles coated with reduced graphene oxide (rGO) (Fig. 15a). This biosensor can detect prostate-specific antigen (PSA) in real-time and selectively. Since the isoelectric point of PSA is approximately 6.8, it is negatively charged in phosphate-buffered saline (PBS, pH = 7.4). When the antibody specifically recognizes and captures the PSA, a negative potential gating effect is induced, increasing the density of holes in the rGO@SFP layer, which ultimately leads to an increase in the current. This mechanism enables the quantitative detection of PSA. The three-dimensional hybridized rGO@SFP structure provides a larger surface area for analyte binding, which significantly enhances the sensor's sensitivity by 3–4 orders of magnitude compared to conventional two-dimensional rGO films. Moreover, the rGO@SFP biosensor maintains high sensitivity and selectivity even under bending. Thus, natural biocomposites are expected to be an integral part of flexible and wearable sensors and improve sensing performance sensitivity.

Nucleic acid aptamers exhibit higher binding affinity, specificity, and stability than conventional antibody-antigen recognition systems. Therefore, Zhang et al. [110] developed a graphene-based aptamer nanobiosensor technology for the rapid detection of hemoglobin (Hb) in biofluids. The sensor utilized PASE to immobile amino-modified Hb aptamer G15. Given that Hb has an isoelectric point of 6.86, it is weakly negatively charged at pH = 7.4. When the aptamer binds to Hb, its structure folds into a compact and stable morphology, bringing the negatively charged Hb close to the graphene surface. This alters the charge distribution at the graphene-liquid interface, inducing an equivalent positive charge on the other side of the interface and generating free-electron carriers in the graphene body, resulting in a detectable change in the source-drain current. Based on this mechanism, the graphene aptamer nanobiosensor could detect Hb in undiluted serum and urine with estimated limits of detection (LODs) of 10.6 fM (1 × PBS), 14.2 fM (undiluted serum), and 11.9 fM (undiluted urine), respectively (Fig. 15b). Additionally, even after 120 cycles of 100% cyclic stretching or one bending cycle with a radius of 100 mm, the sensor's excellent flexibility and stretchability maintained nearly unchanged signals at each Hb detection concentration. Therefore, this aptamer nanobiosensor holds promise for implantable and wearable continuous real-time hemoglobin monitoring applications.

In addition to detecting protein molecules, therapeutic functions can be integrated into wearable bioelectronic devices to provide on-demand treatment based on real-time detection and feedback control of disease biomarkers. For example, Jang et al. [69] developed a contact lens based on GFETs that not only detects matrix metalloproteinase-9 (MMP-9) but also treats ocular surface inflammation (OSI). The sensor formed a continuous network of ultra-long silver nanofibers (AgNFs) prepared by electrospinning technology as source and drain electrodes, with pristine graphene fabricated by CVD technology as the semiconductor layer. This smart contact lens, modified with an immunoglobulin G (IgG) antigen-binding fragment that specifically identifies MMP-9, facilitates real-time quantification of MMP-9 concentration, a biomarker of OSI. In addition, a clear, stretchable heating patch based on AgNFs was integrated into the FET smart contact lens device, which fits comfortably on the eyelid and immediately automates OSI treatment based on detection feedback (Fig. 15c). Both the diagnostic and therapeutic functions can be wirelessly controlled via a programmed application software, realizing a technological leap forward for bioelectronic wearable devices.

##### Cytokine Detection

**Fig. 16:**
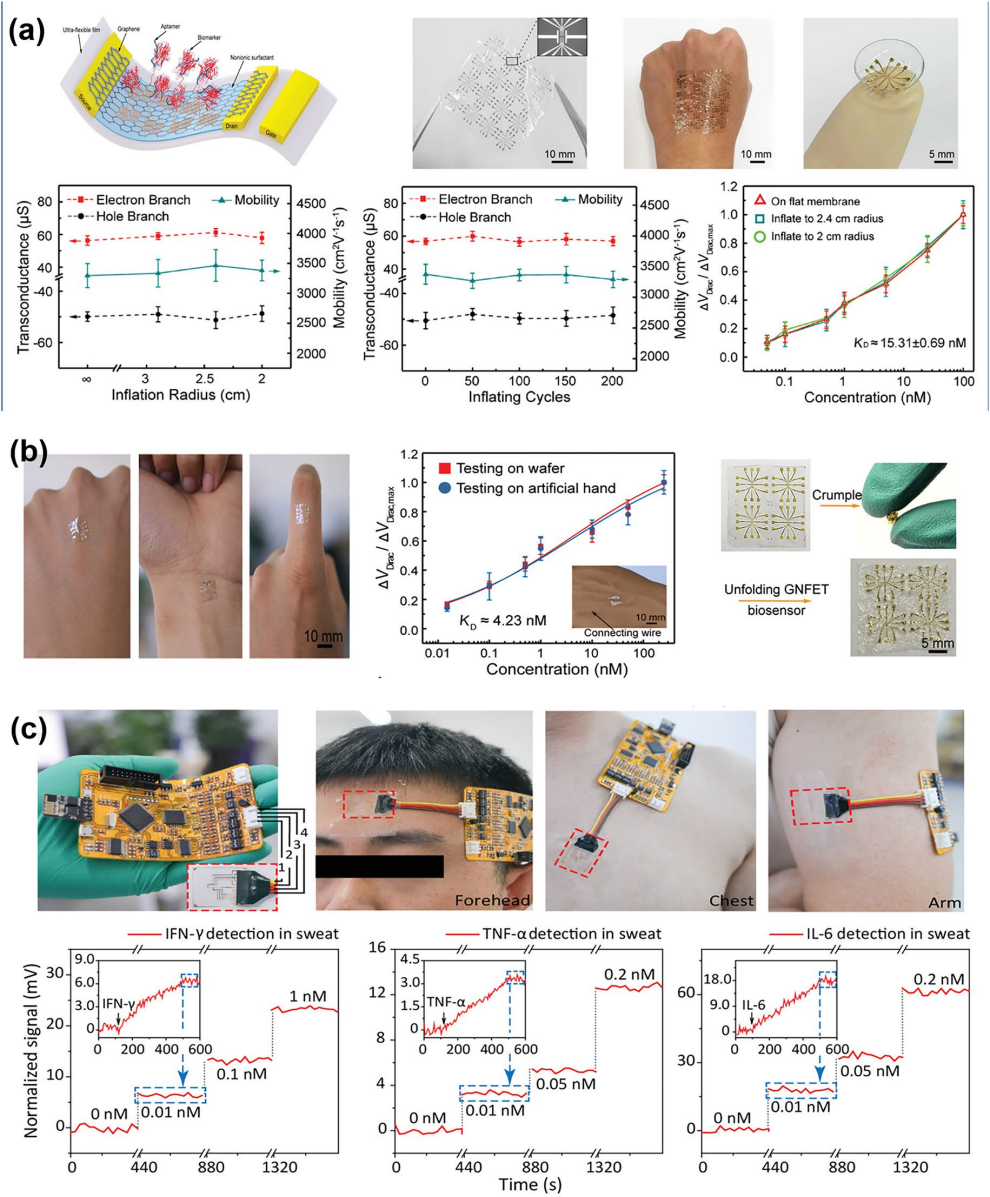
FGFET-based wearable biomedical sensors for cytokine detection. **a** Flexible and regenerative aptameric field-effect transistor biosensor conformably mounted on the human hand and contact lens. Transconductance and carrier mobility of the GFET nanosensor at different inflation radii. Transconductance and carrier mobility of the sensor after differernt inflating cycles. Measurements of TNF-α protein with concentration at different inflation radii. Reproduced with permission from Ref. [277], Copyright 2019, WILEY–VCH. **b** A flexible and regenerative aptameric graphene–Nafion field-effect transistor biosensor for IFN-γ detection in human sweat. Reproduced with permission from Ref. [278], Copyright 2021, WILEY–VCH. **c** Flexible aptamer DGTFET biosensing for cytokine detection. This fully integrated device can be comfortably worn on different parts of the body such as the forehead, chest and arms. It responds well to different concentrations of cytokines (including IFN-γ, TNF-α and IL-6) in human sweat. Reproduced with permission from Ref. [68], Copyright 2021, WILEY–VCH

Cytokines are a class of protein molecules produced by immune cells and other cells, which can act as signaling molecules to transmit information between cells, regulate immune cell activities and interactions, and play a key role in immune responses and inflammatory processes. In general, cytokines are mostly detected by aptamers for specific recognition and capture. Similar to the principle of conventional aptamer-based protein detection, this kind of detection relies on the change of charge density inside graphene caused by the charge of the cytokine itself and the conformational change of the aptamer during the detection process, so as to realize the detection of cytokines. Currently, FGFET biosensors have successfully detected various cytokines, such as tumor necrosis factor-α (TNF-α) [245], vascular endothelial growth factor (VEGF) [275], gamma interferon (IFN-γ) [276], and interleukins [182]. However, for wearable field-effect transistors, achieving good detection is not enough; they must also possess mechanical flexibility to be applied on human skin or implanted in the body, enabling the collection of clinical-grade data. Therefore, Wang et al. [277] fabricated an ultra-flexible stretchable graphene field-effect transistor nanosensor on the 2.5 μm polyester film. Based on an ultrathin substrate, the nanosensor was able to fit onto the underlying surface (e.g., human tissue or skin surface) and maintained good electrical and sensing properties when deformation occurred. It was able to detect TNF-α protein stably and reliably even under large deformation with a LOD of 5 × 10^–12^ M (Fig. 16a). With these capabilities, the nanosensor holds potential for use in wearable or implantable systems for human health monitoring and clinical diagnostics. Nevertheless, these sensors face limitations in detecting cytokines in physiologically relevant environments, especially in wearable applications, owing to the low concentration of cytokines and the interference of background substances in human biofluids such as sweat, tears, and saliva. To overcome this challenge, this group further developed a flexible and self-regenerating aptameric FET biosensor [278]. This biosensor utilizes a graphene-Nafion composite film to minimize non-specific adsorption and improve the reproducibility of the biosensor. As a result, the device achieved stable, sensitive detection of interferon-γ (IFN-γ) in undiluted human sweat with a detection limit as low as 740 fM (Fig. 16b). Moreover, it maintained a consistent sensing response throughout both regeneration (up to 80 cycles) and crumpling (up to 100 cycles) tests. The biosensor offers potential for the creation of wearable biosensing systems that can distinguish between patients with acute infectious diseases and monitor the health status of individuals in their everyday lives. Similarly, Zhang et al. [68] proposed an intelligent adaptor dual-channel graphene-tween 80 field-effect transistor (DGTFET) biosensing device. Benefiting from the differential measurement design and the TWEEN 80 passivation layer, the device can effectively minimize or eliminate erroneous response signals from unwanted background interferences in biofluids, thereby enabling the detection of cytokines (including IFN-γ, TNF-α, and IL-6) in human biological fluids such as serum, saliva, urine, and sweat. Additionally, through customized circuits and an Android application, the adaptor DGTFET biosensor, fabricated on ultra-thin flexible PET film, can be remotely controlled by physicians via Wi-Fi and comfortably worn on different parts of the body. It allows for real-time monitoring of patient's physical condition by detecting the levels of cytokines in sweat (Fig. 16c). This DGTFET holds significant potential in wearable sweat cytokine monitoring applications.

##### Glucose Detection

The use of continuous glucose monitoring devices for the treatment of insulin-dependent diabetes makes the development of wearable and flexible glucose sensors essential [279, 280]. Invasive and non-invasive glucose sensors have proven to be in high demand for accuracy, long-term stability, adaptability, and comfort. Recently, enzymatic glucose biosensors have attracted great interest due to the high selectivity of enzymes and their rapid response to specific substrates. Highly selective glucose biosensors that depend on enzymatic biometric events can be implemented at a relatively low cost [281]. By modifying glucose oxidase, FGFET has been successfully used to fabricate high performance enzymatic glucose sensors.

**Fig. 17:**
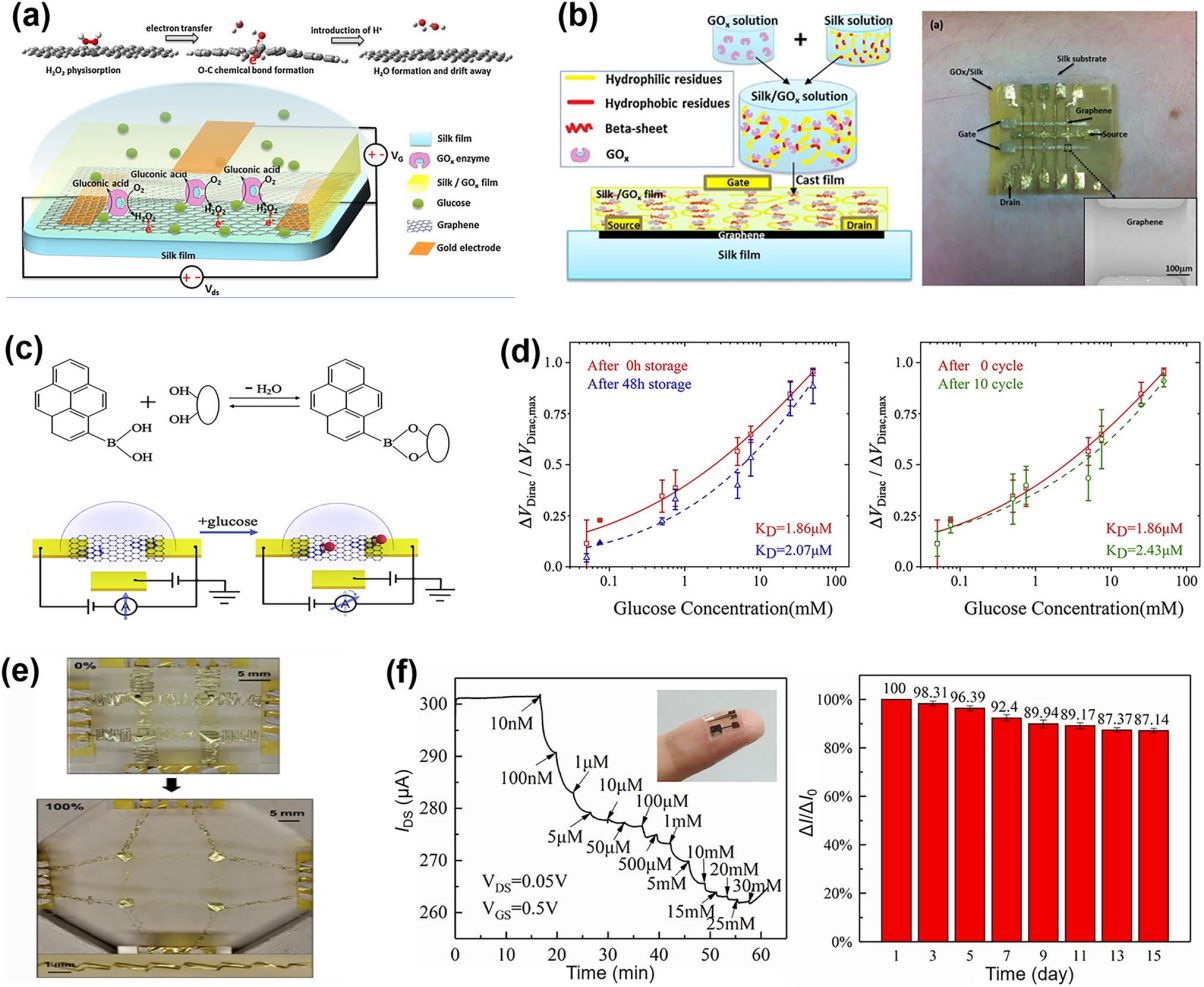
FGFET-based wearable biomedical sensors for glucose detection. **a** Detection mechanism of the FGFET-based enzymatic glucose sensor. **b** Schematic and optical image of silk–based graphene FET glucose biosensors attached onto the wrist. Reproduced with permission from Ref. [113], Copyright 2014, Elsevier Ltd. **c** Schematic diagram of the PBA functionalized graphene FET glucose-sensing method. **d** Characterization of the consistency for glucose detection using the PBA functionalized GFET nanosensor after extended-time storage and multiple reuse cycles. Reproduced with permission from Ref. [282], Copyright 2020, Elsevier Ltd. **e** Photographs of the kirigami-patterned stretchable sensor in neutral state and 100% biaxially stretched state. Reproduced with permission from Ref. [284], Copyright 2020, Springer Nature. **f** Sensitivity and stability of flexible gel electrolyte graphene transistors for glucose detection. Reproduced with permission from Ref. [285], Copyright 2023, Elsevier Ltd

In general, the detection mechanism of the FGFET-based enzymatic glucose sensor is depicted in Fig. 17a [113, 114]. When glucose is present, glucokinase (GOx) immobilized on the graphene surface catalyzes the oxidation of glucose according to the reaction Eq. (5) to produce gluconic acid and H_2_O_2_. The generated H_2_O_2_ induces an electrical reaction (Dirac point shift, current change,) in the graphene field-effect tube, which leads to the indirect detection of glucose.

5 $${\text{D}} - {\text{glucose}}\, + \,{\text{O}}_{{2}} \, + \,{\text{H}}_{{2}} {\text{O}}\, \to \,{\text{D}} - {\text{gluconic acid}}\, + \,{\text{H}}_{{2}} {\text{O}}_{{2}}$$

Kwak et al. [281] processed a flexible glucose sensor using CVD graphene, a prime example of an enzymatic glucose sensor. When glucose oxidase recognizes glucose and breaks it down to produce H_2_O_2_, the H_2_O_2_ molecule undergoes direct electron transfer with graphene creating an n-doping effect. In addition, adsorption of this H_2_O_2_ graphene acts as a source of scattering against mobile electrons and reduces the FET conductance. As a result, the sensor is capable of achieving a sensitive response to glucose. The glucose was detected by bending the flexible sensor to a radius of curvature of 6.625 cm, and the response signal was highly consistent with that in the planar state. The application of enzymatic biosensors is often hindered by the lack of long-term enzyme stability and low enzyme loading under measurement conditions. To address this issue, You et al. [113] constructed a flexible GFET glucose sensor using silk protein as a device substrate and an enzyme immobilization matrix. Due to the unique structure of the silk protein, it can load more enzymes and maintain their activity, improving the sensitivity of the sensor and enabling it to detect glucose levels in the range of 0.1–10 mM. The silk substrate is highly mechanically flexible, allowing the biosensor to fit snugly on curved surfaces such as the human wrist (Fig. 17b). Furthermore, the silk matrix offers the advantage of adjustable dissolution and biodegradation rates, spanning from hours to years, and produces non-inflammatory amino acid degradation products. These properties make the sensor suitable for use in wearable or implantable biosensor applications, including continuous glucose level monitoring patches. However, while the use of silk protein immobilization increases the enzyme load, its accuracy and stability are still affected by the inevitable enzyme inactivation. It has been shown that pyrene-1-boronic acid (PBA) can induce electrical changes in graphene through specific reactions with glucose, converting it to glucose-boronic acid ester complexes. Specifically, glucose can bind to PBA molecules and convert them from the neutral form to the negatively charged borate form (Fig. 17c). The increase in charge density of the PBA molecules leads to an increase in graphene carrier density [282, 283]. Based on this, Huang et al. [282] functionalized the channels of graphene GFETs using specific pyrene-1-boronic acids (PBAs) to achieve a non-enzymatic detection of glucose. The sensor is simpler to prepare than the more common enzymatic strategy and offers improved stability and accuracy for long-term glucose monitoring. The device demonstrates high sensitivity and a low detection limit of 0.15 μM on a flexible polyimide substrate. Additionally, extended storage periods (over 48 h) have negligible effects on its sensing performance (Fig. 17d). The experimental results indicate that the GFET nanosensor maintains good consistency and reliability in glucose detection even after prolonged storage.

Graphene-based wearable glucose sensors may be deformed by normal body movements when affixed to the surface of the skin, leading to potential disruption of their electrical signals. Recent studies have emphasized enhancing the mechanical stability of wearable glucose sensors based on graphene. Lee et al. [284] proposed a new method for fabricating graphene FET structures based on a "kirigami" pattern-a three-dimensional structure formed by cutting on a two-dimensional sheet, similar to origami (Fig. 17e). This Kirigami paper pattern not only normalized the resistance variations resulting from the combination of graphene and metal electrodes in the FET sensor, but also enhanced its mechanical stability. In their experiments, the resistance change at 100% biaxial stretching and 180° bending was less than ≈0.5%. The solution-gated GFET glucose sensor with this multiaxial stretchable Kirigami pattern has a high sensitivity of − 24 mV dec^−1^ and can effectively measure glucose concentrations from 1 pM to 1 mM. In addition, it minimizes motion artifacts, thereby improving the accuracy of glucose concentration measurements. The versatility of this approach allows it to be extended to a variety of other biosensing applications, making it an ideal candidate for wearable biosensors. Furthermore, sample collection is a crucial practical issue for non-invasive wearable blood glucose sensors. Gao et al. [285] have designed a skin-based flexible gel electrolyte graphene transistor (GEGT) for glucose detection. This sensor utilizes glycerol gel instead of traditional liquid electrolytes, which not only conforms better to human skin but also acts as a liquid collector, providing stable testing conditions. After 15 days, its current response to glucose remained above 85% of its initial value (Fig. 17f). Furthermore, when untreated sweat was directly applied to the surface of the prepared device, the measurement results were consistent with those obtained using a commercial glucose detection kit after impurity removal, with an RSD of less than 3%, further verifying the high accuracy of the device in sweat glucose detection. The GEGT sensor shows great precision in detecting sweat and has a responsive performance while being worn, suggesting its promising future in non-invasive smart medical monitoring through wearables.

In summary, FGFETs have demonstrated considerable potential in the field of blood glucose detection, offering advantages such as high sensitivity, miniaturization, real-time monitoring, low power consumption, and low cost. However, due to the complexity of the manufacturing process and their sensitivity to environmental conditions, the application of FGFETs remains in its early stages. Despite this, current studies indicate that FGFET sensors have already achieved accuracy comparable to existing optical and electrochemical biosensors [286, 287]. It is important to note, however, that traditional electrochemical and chemical blood glucose sensors typically maintain stability for 15–45 days [286, 288, 289], with some extending up to 90 days [287]. In comparison, FGFETs still face significant challenges in maintaining stability during blood glucose detection. To address this gap, efforts should be directed toward optimizing enzyme immobilization techniques, enhancing the surface treatment of graphene, and developing more robust non-enzymatic detection strategies. By doing so, we believe the stability of FGFET sensors will be significantly improved, potentially reaching or even surpassing the performance levels of existing technologies.

#### Nucleic Acids and Other Biological Molecule Detection

**Fig. 18:**
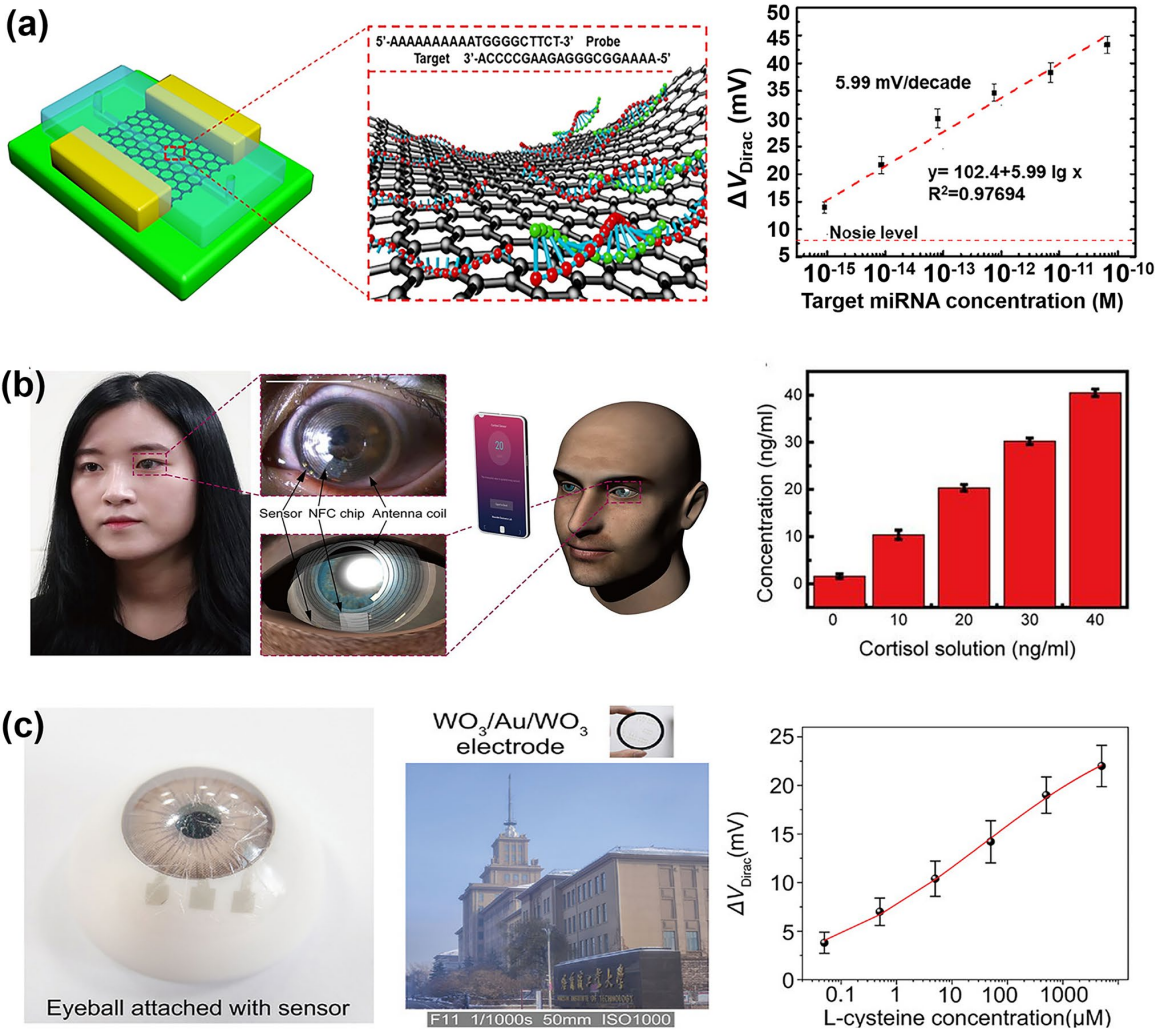
FGFET-based wearable biomedical sensors for nucleic acids and other biological molecule detection. **a** Schematic structure and sensitivity of the miRNA sensor. Reproduced with permission from Ref. [290], Copyright 2020, WILEY–VCH. **b** Schematic image of the cortisol level measurement using the smart contact lens. Cortisol concentration was measured using the contact lens sensor as a function of the concentration of the cortisol solution that was dropped into the eye. Reproduced with permission from Ref. [291], Copyright 2020, American Association for the Advancement of Science. **c** Photograph of the ultra-flexible and transparent GFET wearable nanosensor attached to the artificial eye. Δ*V*_Dirac_ of the flat, bend, fold, and shrink nanosensor as a function of the L-cysteine concentration. Δ*V*_Dirac_ of the nanosensor as a function of the L-cysteine concentration in artificial tears. Reproduced with permission from Ref. [292], Copyright 2022, WILEY–VCH

Nucleic acids (DNA, RNA, miRNA) are important regulatory molecules that regulate the expression of other functional genes and are involved in various physiological activities as well as the growth and development of organisms, and their expression levels are associated with various diseases and are promising biomarkers for disease progression. Therefore, the study of high-throughput nucleic acid detection technologies is crucial for biological research and clinical diagnosis. Our group has built a customizable Gr-FET biosensor for label-free detection of miRNAs [290]. Unlike other sensors, we designed a special DNA probe. It has 10 adenine bases at the 5' end that can be immobilized on the graphene channel by π-π interactions, while the 3' end recognizes the target miRNA and hybridizes to the miRNA fraction. Since the phosphate carbon backbone of miRNA is rich in negative charge, when the DNA probe specifically recognizes miRNA, the charge transfer between miRNA and graphene realizes n-type doping of graphene, which in turn leads to the left shift of the Dirac point. Based on this mechanism, the Gr-FET biosensor is capable of sensitive detection of miRNA in less than 20 min without any functionalization and labeling (Fig. 18a). In addition, the entire miRNA sensor is constructed on a flexible polyimide (PI) substrate, which exhibits stable performance even after multiple mechanical bends. This property makes it promising for integration into wearable health monitoring electronic devices.

Cortisol is a key hormone linked to stress, with its levels serving as a crucial barometer for mental health. It is present in tears, sweat, and saliva in large quantities, making it essential to monitor its levels to comprehend the body's hormonal reaction to stress. Zhang et al. [100] modified aptamer probes on flexible liquid-gated graphene field effect transistors (Lg-GFETs) and utilized these probes to capture cortisol molecules. Since cortisol has an isoelectric point between pH5.2 and pH5.4, it is negatively charged under physiological conditions (pH7.35 to pH7.45). When the aptamer traps cortisol, it dopes the negative charge into the graphene channel, which reduces the carrier concentration in the channel, leading to a decrease in the *I*_ds_ and enabling the detection of cortisol. The flexible salivary cortisol detection platform of this Lg-GFET can fulfill the requirement of immediate salivary cortisol detection and provides an alternative solution for the development of similar wearable home medical devices. Similarly, Ku et al. [291] developed a soft, smart contact lens designed to non-invasively monitor cortisol levels in tears in real-time using a graphene-based FET sensor. In this sensor, cortisol antibodies are used to modify the graphene channel, and upon selective attachment of cortisol, electrons are injected into the graphene channel, resulting in a lower drain current. This lens can detect cortisol levels as low as 10 pg mL^−1^, a low detection limit. Animal tests using live rabbits and human trials have demonstrated the excellent biocompatibility and reliability of the lens as a non-invasive mobile healthcare solution (Fig. 18b). As for practicality, this soft contact lens only needs to integrate the cortisol sensor with a transparent antenna and wireless communication circuits, allowing a smartphone to be the only device required for remote operation of the lens, without hindering the wearer's line of vision. This represents a substantial technological leap forward, merging user-friendly device control with critical health monitoring.

L-cysteine levels and metabolic status are associated with a wide range of physiologic and pathologic processes, making their monitoring and detection important in medical and biological research. Huang et al. [292] proposed an ultra-flexible and transparent GFET wearable nanosensor. The sensor successfully identified L-cysteine not only in undiluted human sweat but also in artificial tears, and the response of the sensing signal remained consistently reliable during the twisting test. Based on an ultra-thin substrate and WO_3_/Au/WO_3_ electrodes with up to 81% transparency, the sensor can be mounted on the eye for tear detection without affecting vision (Fig. 18c). This ultra-flexible and transparent GFET wearable nanosensor holds promise for various medical detection uses.

#### Metal Ions Detection

**Fig. 19:**
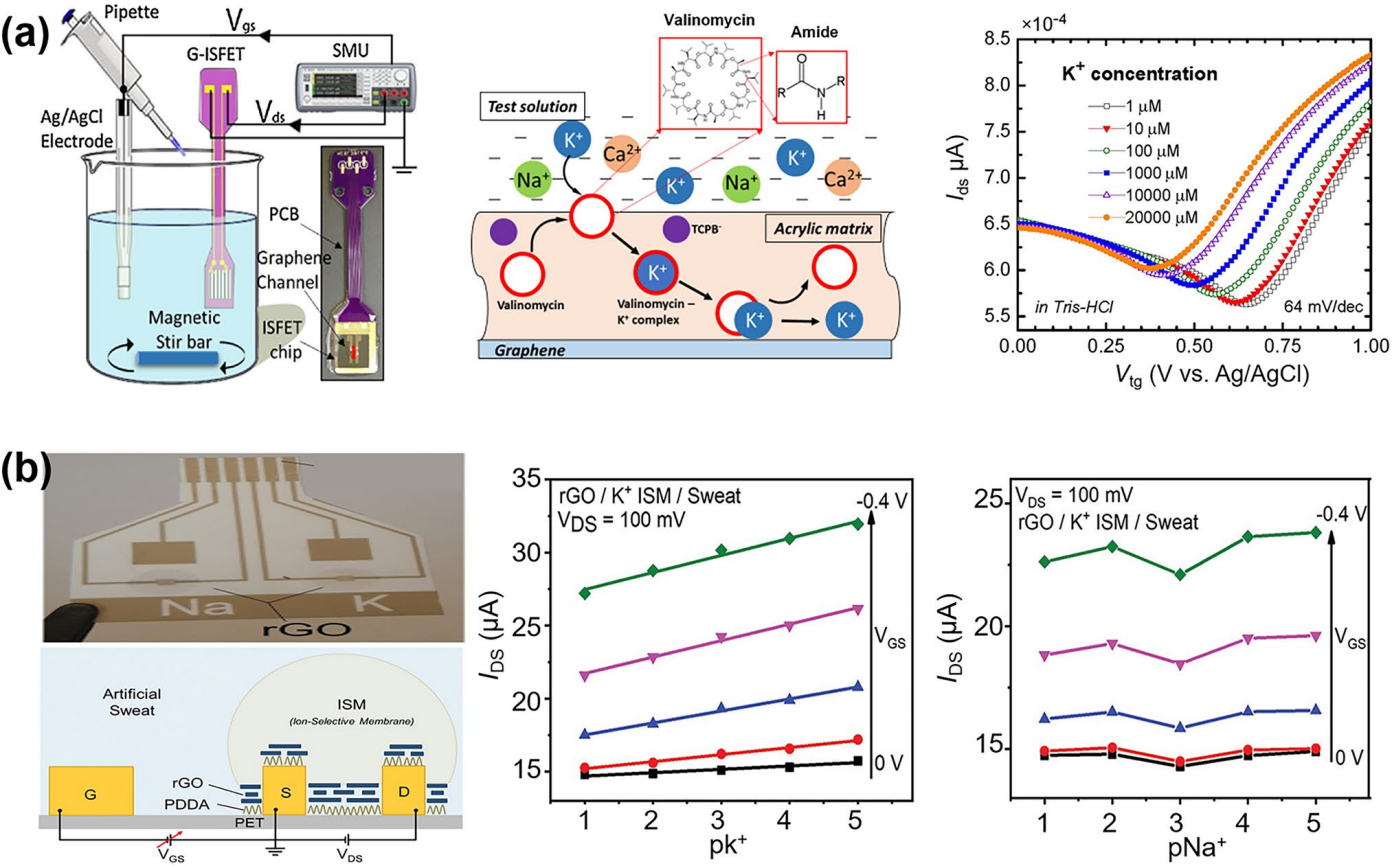
FGFET-based wearable biomedical sensors for metal ion detection. **a** Sensing mechanism and sensing performance of GISFET for detecting potassium ions. Reproduced with permission from Ref. [297], Copyright 2017, Elsevier Ltd. **b** Schematic and photograph of the rGO LGT cross-section employing an ISM and its sensitivity to K^+^ and Na^+^ concentration in artificial sweat with K- and Na-addition. Reproduced with permission from Ref. [299], Copyright 2022, WILEY–VCH

Alkali metal ions play a crucial role in biological structures and metabolic activities and are extremely important for humans, thus their detection is of great significance [293]. The detection of alkali metal ions is even more challenging because of the requirement of selective functionalized layers. Ion selective membranes (ISMs) have been widely used for the selective determination of ions [294, 295]. ISMs depend on the specific characteristics of the ion exchanger, such as valinomycin, which is one of the most commonly used potassium ion carriers that can confer membrane ion specificity [296, 297]. Li et al. [297] used valinomycin-based membrane coatings to prepare highly sensitive and selective graphene-based ion-sensitive field-effect transistors (GISFETs) (Fig. 19a). The structure of valinomycin, composed of alternating amide and ester bridges, features a unique donut-shaped conformation that enables it to bind metal ions. Due to the size of potassium ions (radius 1.33 Å) being a perfect match for valinomycin's structure, valinomycin exhibits a very high stability constant (K = 10^6^) for potassium ions compared to other metal ions. Additionally, the valinomycin ion-selective layer possesses specific permeability for potassium ions. Therefore, when the GISFET specifically recognizes potassium ions, they permeate the graphene surface, inducing a higher electron concentration (i.e., reducing the hole concentration in p-type graphene). This shift brings the Fermi level closer to the charge neutrality point, thereby lowering the Dirac point and enabling potassium ion detection. Furthermore, the sensor maintains effective performance in physiological solutions, making it suitable not only for wearable sensing devices but also promising for the development of flexible graphene-based bio-implantable ISFETs.

Despite the exceptional performance of GISFETs as cation sensors [296], the majority of these devices are currently fabricated using laboratory-scale methods that do not meet the stringent requirements of wearable chemical sensing technology [298]. Oliveira et al. [299] prepared plastic rGO liquid gate transistors (LGTs) using laser micromachining techniques. The use of rGO as an electron transducer and the coating of its surface with ion-selective films of potassium and sodium ions enabled real-time monitoring of potassium and sodium levels in artificial sweat (Fig. 19b). Based on the inherent physicochemical properties of rGO, including water-solubility, non-irritation to the skin, and tunable electrical characteristics, the device met the requirements for industrial scalability and is highly beneficial for wearable electronic devices, especially in continuous cation monitoring situations, such as sweat analysis during exercise, which can provide valuable physiological information for health monitoring.

Table 2 highlights several representative FGFET biosensors developed for the detection of biochemical parameters. By employing appropriate probes such as antibodies, aptamers, and ssDNA, these biosensors are capable of detecting a broad range of biochemical markers, including proteins, cytokines, glucose, nucleic acids, cortisol, L-cysteine, and metal ions, with LODs as low as fM, fM, μM, fM, pg/mL, μM, and 1 μM, respectively. Furthermore, FGFET-based sensors can operate in various biofluids, including serum, urine, tears, sweat, and saliva, making them versatile tools for diverse biological environments. FGFET-based wearable biomedical sensors have significantly advanced the detection of biochemical parameters, holding substantial promise for applications in medical monitoring, health management, and disease diagnosis. As these technologies continue to progress, FGFET sensors are poised to play an increasingly vital role in both clinical practice and personal health management.

**Table 2:** Summary of FGFET-based wearable biomedical sensors for detection of biochemical parameters

Analyte		Sensing material	Probe	Biofluid	Dynamic range	LOD	Refs
Protein	PSA	rGO@SFP	Antibodies	–	1.7 × 10^−15^ − 1 × 10^−9^ M	1.7 × 10^−15^ M	[274]
	Hb	CVD-graphene	Aptamer	Undiluted serum and urine	1 pM − 1 μM	10.6 fM (1 × PBS), 14.2 fM (undiluted serum), 11.9 fM (undiluted urine)	[110]
	MMP-9	CVD-graphene	Antibodies	Tear	1 − 500 ng mL^−1^	0.74 ng mL^−1^	[69]
Cytokine	TNF-α	CVD-graphene	Aptamer	–	50 × 10^−12^ − 100 × 10^−9^ M	5 × 10^–12^ M	[277]
	IFN-γ	CVD-graphene	Aptamer	Sweat	0.015 – 250 nM	740 fM	[300]
	IFN-γ, TNF-α, IL-6	CVD-graphene	Aptamer	Serum, saliva, urine, and sweat	0.1 × 10^−9^ – 500 × 10^−9^ M	476 × 10^−15^ M (IFN-γ), 608 × 10^−15^ M (TNF-α), or 611 × 10^−15^ M (IL-6)	[68]
Glucose		CVD-graphene	GOx	–	3.3 − 10.9 mM	3.3 mM	[281]
		CVD-graphene	GOx	–	0.1 − 10 mM	0.1 mM	[113]
		CVD-graphene	PBA	–	1 μM − 5 mM	0.1 μM	[283]
		CVD-graphene	PBA	–	0.05 – 100 mM	0.15 μM	[282]
Nucleic acid	MiRNA	CVD-graphene	Single-stranded DNA	–	1 fM – 100 pM	10 fM	[290]
Cortisol		TCPP@G-ink	Aptamer	Saliva	0.08 – 800 nM	–	[100]
		CVD-graphene	Antibodies	Saliva	1 – 40 ng mL^−1^	10 pg mL^−1^	[291]
L-cysteine		CVD-graphene	Aptamer	Sweat/Artificial tears	0.1 – 1000 μM	0.022 × 10^−6^ M (undiluted sweat), 0.043 × 10^−6^ M (artificial tears)	[292]
Metal ion	K^+^	CVD-graphene	ISM	–	1 μM – 20 mM	1 μM	[297]
	K^+^/ Na^+^	rGO	ISM	Artificial Sweat	10 µm – 100 mM	10 µM	[299]

### FGFET-Based Implantable Biomedical Sensors

Implantable sensors distinguish themselves from wearable devices in their monitoring of physiological and biochemical parameters. These sensors typically offer higher data precision and reliability, with the capability for 24-h continuous surveillance, unswayed by external environmental factors [301]. Post-implantation, these sensors usually operate without user intervention, effectively eliminating user adherence-related issues. Additionally, situated within the internal environment, these sensors are less likely to be affected by external physical interference. Graphene's flexibility allows it to form soft, adaptable interfaces that closely correlate with the mechanical properties of the cells, thereby improving the compatibility and long-term reliability of biological interfaces. This has driven the development of implantable sensing devices. As a result, a variety of FGFETs have been used in recent years for implantable applications, including electrophysiological recording and in vivo neurochemical detection.

#### Bioelectric Signal Monitoring

**Fig. 20:**
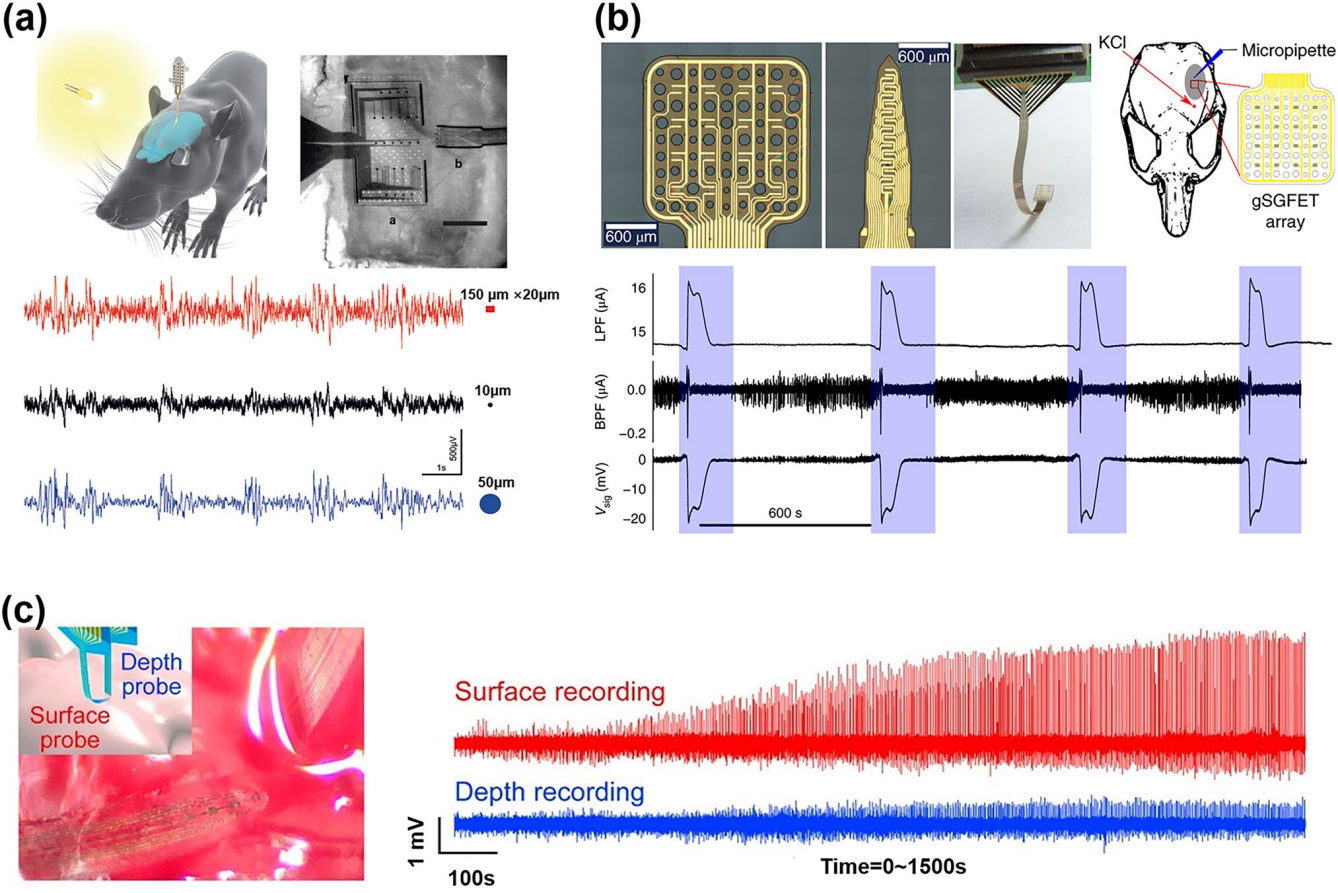
FGFET-based implantable biomedical sensors for bioelectric signal monitoring. **a** Simultaneous recordings of a graphene transistor showing bicuculline-induced brain activity. Reproduced with permission from Ref. [70], Copyright 2017, IOP Publishing Ltd. **b** gSGFETs for both epicortical and intracortical mapping of CSD. Reproduced with permission from Ref. [308], Copyright 2019, Springer Nature. **c** Dual-modality recording with graphene transistor-based neural probes. Reproduced with permission from Ref. [71], Copyright 2018, Elsevier Ltd

Research has also shown that graphene can promote the adhesion and regeneration of damaged neurons, aiding the healing process. Graphene may reduce the proliferation of glial cells around the device and improve coupling with target neurons [302], leading to high-quality electrophysiological recordings [303]. Consequently, various FGFETs have been utilized in implantable applications to record neural activity over the past few years [70, 304–307]. For example, Blaschke et al. [70] successfully used flexible graphene micro-transistors to map brain activity in rats (Fig. 20a). The use of solution-gated FETs (SGFETs) made from graphene allows for high-density integration and superior signal-to-noise ratio (SNR) in brain activity recordings. Graphene SGFETs can record spontaneous slow waves, visually evoked responses, and pre-epileptic activity in vivo in rats. The SNR achieved by graphene SGFETs is comparable to that of state-of-the-art platinum electrodes, and these transistors exhibit intrinsic signal amplification and bipolar behavior. Furthermore, graphene implants are biocompatible, causing no significant inflammation. Graphene-based SGFET technology potentially paves the way for future high-density neural recording implants, which are crucial for mapping brain activity, understanding behavioral studies, and developing neural prosthetics. Although graphene field-effect transistor arrays meet most requirements for μ-ECoG recordings and offer the potential for developing multiplexing technologies, existing systems still cannot provide the resolution needed to accurately map highly localized brain activities. This limitation arises because the size of connectors increases rapidly with the number of sensors. To address this issue, Schaefer et al. developed flexible graphene arrays. They created flexible graphene SGFET arrays and explored the concept of multiplexed readouts using an external switching matrix. This method not only increased the number of sensors but also significantly reduced the complexity of fabricating devices on flexible substrates, paving the way for scaling up SGFET technology to achieve large-scale, high-density μECoG arrays. Moreover, evaluations of these graphene SGFETs in anesthetized Long Evans rats confirmed the fidelity of broadband signal representation (infra-slow oscillations and local field potentials) during multiplexed operations, with signal quality comparable to state-of-the-art commercial neural sensors. Therefore, apart from their potential as effective neural activity transducers, graphene SGFETs also hold significant promise as foundational components for high-sensor-count multiplexed brain-machine interfaces.

Tracking brain activity below 0.1 Hz, known as low-frequency activity (ISA), is crucial for clinical purposes in neurocritical care, aiding in diagnosis, prognosis, and treatment. ISA provides insights into brain conditions like sleep or coma. Cortical spreading depression (CSD) is a gradual wave of nearly complete depolarization in neurons and astrocytes, followed by a phase of electrical activity suppression. While not common in brain physiology, CSD is often induced in individuals with stroke, brain trauma, and migraines. To record ISA in vivo, Masvidal-Codina et al. [308] utilized graphene solution-gated field-effect transistor (gSGFET) arrays to map CSD both on the cortical surface and within the cortex (Fig. 20b). Their results demonstrated that graphene transistors provide excellent spatial resolution for mapping ISA and can record a wide bandwidth from low frequencies to the typical local field potential range. By integrating gSGFETs with optical techniques such as laser speckle contrast imaging, a two-dimensional map of neurovascular coupling can be obtained, offering significant insights into this phenomenon.

Neural probe technology is essential for advancing both basic neuroscience research and brain-machine interfaces (BMIs). Deep intracortical recordings offer detailed insights into neural activity, greatly enriching our knowledge of the neuronal mechanisms underlying behavior. Despite their informative nature, deep probes can provoke inflammation and neuronal damage in the brain due to their invasive nature. In contrast, subdural surface probes have emerged as a non-invasive alternative, positioned directly on the cortical surface. These surface probes capture combined signals from multiple neurons beneath, offering a less invasive approach compared to deep probes. They have found application in clinically diagnosing neurological conditions like epilepsy. To understand the relationship between these two modalities, Du et al. [71] developed flexible dual-modal neural probes based on graphene transistors (Fig. 20c). Due to graphene's excellent mechanical properties, these neural probes exhibit stable electrical performance even when bent at 90°, allowing for multi-site recordings from the subdural surface of the rat cortex. Finite element analysis indicated that a tip angle of π/6 facilitates the penetration of neural probes into tissue. Consequently, the graphene transistor-based dual-modal neural probe was successfully used for simultaneous surface and depth recordings of epileptiform activity in the rat brain in vivo. The graphene transistor-based dual-modal neural probe can serve as a simple and versatile tool for studying the spatiotemporal patterns of neural activity. Compared to using single-modal probes, the dual-modal probe based on graphene transistors is easier to implement and allows for direct comparison of surface and depth neural activities.

#### Neurochemicals Detection

**Fig. 21:**
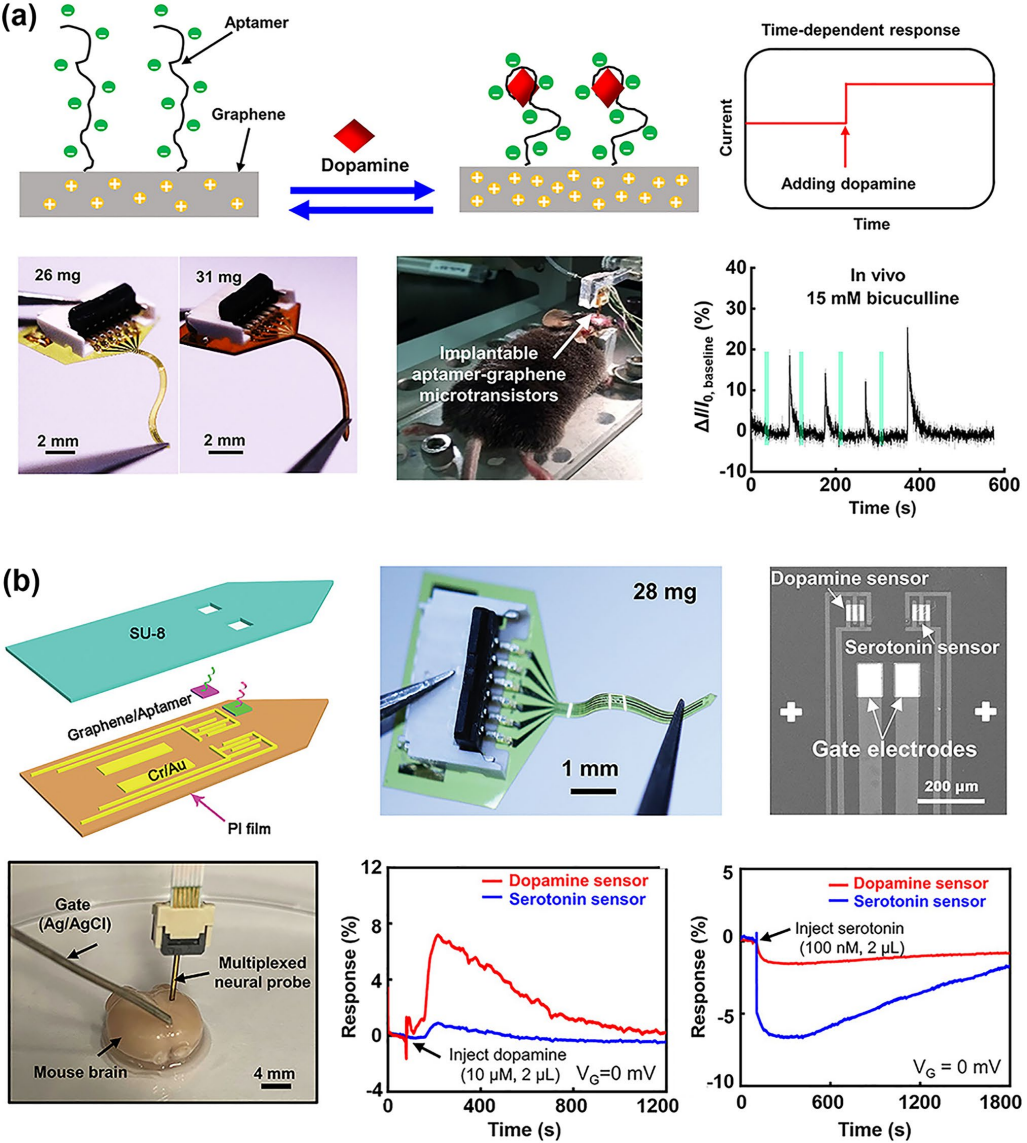
FGFET-based implantable biomedical sensors for neurochemicals detection. **a** Sensing mechanism and sensing performance of aptamer-graphene microtransistor probes for in vivo dopamine detection. Reproduced with permission from Ref. [315], Copyright 2022, American Chemical Society. **b** Multiplexed neural probe implanted into a harvested mouse brain tissue for monitoring dopamine and serotonin. Real-time response of a multiplexed neural probe implanted in the harvested mouse brain tissue upon the injection of dopamine solution and serotonin solution. Reproduced with permission from Ref. [38], Copyright 2022, American Chemical Society

The intricate processes of cognition, perception, and action rely on the dynamic neural networks in the brain, governed by a variety of neurochemicals [309, 310]. The brain contains over 200 identified neurochemicals, including monoamines (such as dopamine and serotonin), peptides, amino acids, lipids, and other small molecules such as acetylcholine [311]. Imbalances in specific neurochemical levels are linked to various neuropsychiatric and neurological conditions like Parkinson's disease [312], schizophrenia [313], and Alzheimer’s disease [314]. As a result, implantable sensors utilizing FGFET technology have been created to detect these neurochemical substances. For instance, Wu et al. [315] reported an implantable aptamer-graphene microtransistor probe. When dopamine binds, it triggers a structural reorganization of the aptamer, causing p-doping effects on the graphene channel (shift to the right) and boosting the source-drain current through electrostatic effect (Fig. 21a). This probe is capable of real-time monitoring of dopamine at near-cellular-level spatial dimensions with high selectivity and picomolar-level sensitivity. In a mouse model, dopamine release in vivo was successfully captured through pharmacological stimulation, suggesting that this neural probe holds promise for applications in basic neuroscience research and the study of processes associated with neurological diseases. Similarly, Gao et al. [38] presented a flexible neural probe designed for simultaneous monitoring of multiple neurochemicals via the electrografting-assisted site-selective functionalization of aptamers on G-FETs (Fig. 21b). Exhibiting exceptional flexibility and ultra-lightweight characteristics, these neural probes can operate at the cellular dimension. The incorporation of electrochemically grafted molecular linkers (− COOH or − NH_2_) and specific aptamers allows for monitoring of serotonin and dopamine with high sensitivity and selectivity. Both in vivo and in vitro studies using harvested mouse brain tissue demonstrate successful multiplexed monitoring of these two substances when a solution of neurochemical substances – dopamine and serotonin – with physiologically relevant concentrations is injected. The advanced multiplexed neural probes can be linked to various organs that necessitate multi-target detection, such as the spinal cord, heart, and peripheral nerves, thereby enabling in vivo detection of bioinformation.

Just like wearable biosensors, FGFET implantable biosensors could be implanted into the human body, allowing for the detection of large biological molecules in serum or tissue fluids, such as proteins, nucleic acids, cytokines, and blood glucose.

#### Long-Term Stability and Biocompatibility of FGFET-Based Implantable Biomedical Sensors

**Fig. 22:**
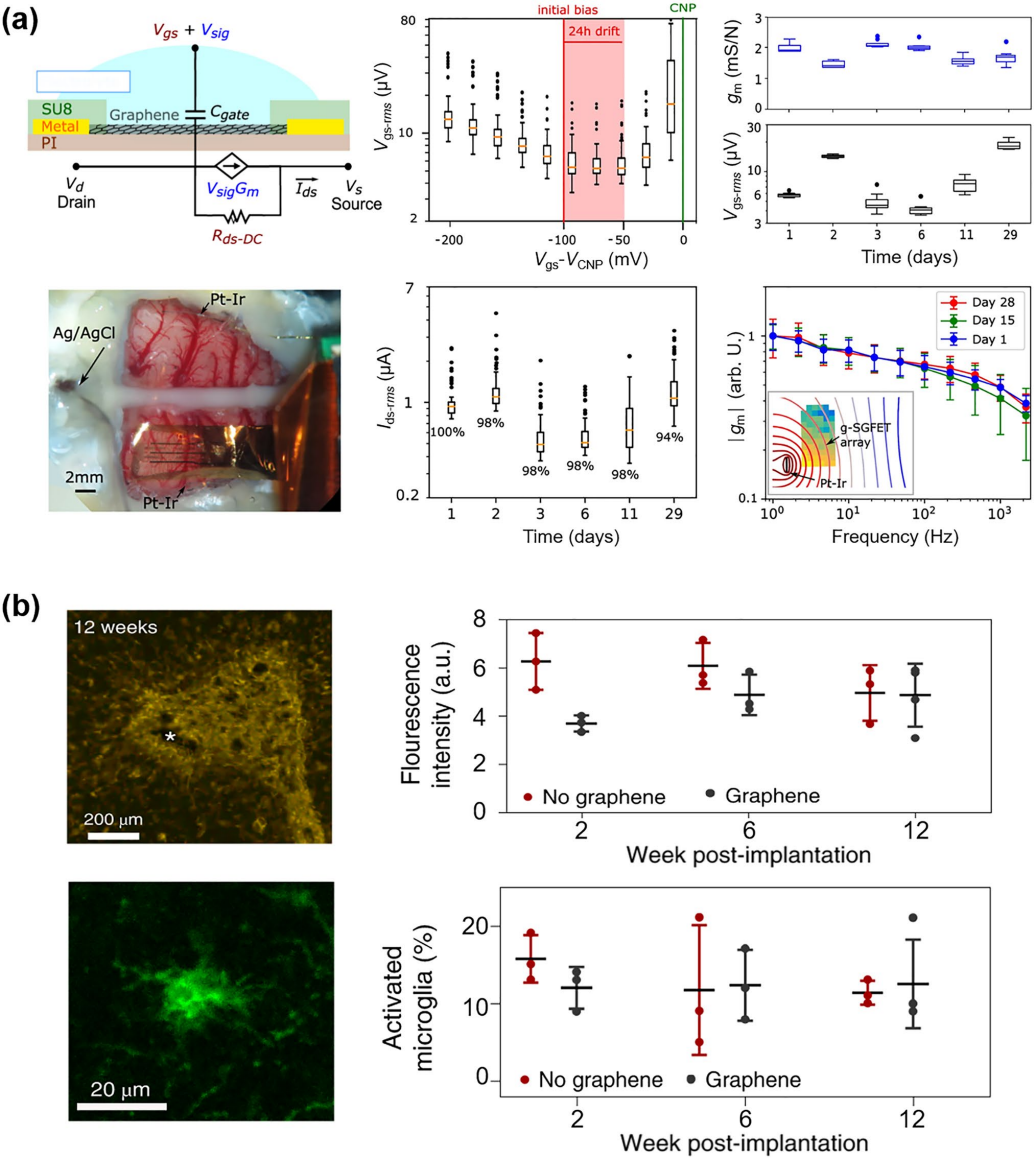
Long-term stability and biocompatibility of FGFET-based implantable biomedical sensors. **a** The Long-term stability of g-SGFET. Reproduced with permission from Ref. [316], Copyright 2021, Springer Nature. **b** Biocompatibility of gDNP. Reproduced with permission from Ref. [324], Copyright 2020, Springer Nature

Implantable biosensors must remain stable over time within the human body, requiring components and materials that can resist physiological conditions such as body fluids and enzymes. Long-term stability is critical to ensuring that the sensor can continuously and accurately monitor biomarkers, as it directly impacts the sensor's ongoing effectiveness. However, maintaining the long-term stability of implantable FGFETs in vivo presents significant challenges. Factors affecting this stability include electrochemical degradation at the graphene-electrolyte interface, adsorption of charged molecules, and fluctuations in the reference electrode potential. To address these challenges, Garcia-Cortadella et al. developed a graphene-based neural probe (Fig. 22a) [316]. By employing a 4-inch wafer-level fabrication process, they precisely controlled the doping of the g-SGFET and the homogeneity of the charge neutrality point, successfully ensuring the sensitivity of the sensor array. Additionally, the application of a 1 mHz high-pass filter effectively reduced the adsorption of charged molecules and mitigated electrode reference potential drift, thus maintaining probe stability over a period of 4 weeks. Beyond design optimization, surface coating techniques are crucial for ensuring the long-term stability of the sensor. For instance, coating the sensor surface with bio-contamination-resistant materials, such as polyethylene glycol and its derivatives [317], zwitterionic polymers [318], or polyacrylamide hydrogels [319], significantly reduces the effects of bio-contamination, further enhancing sensor stability in complex in vivo environments. Moreover, the choice of encapsulation technology is essential; using biocompatible and waterproof materials to protect electronic components not only prevents short-circuiting or damage but also effectively prolongs the sensor's service life [71].

Biocompatibility is a key factor in determining whether a sensor material will interact harmfully with human tissues. Highly biocompatible materials can significantly reduce post-implantation inflammatory reactions and minimize the risk of immune rejection, thereby improving the safety and comfort of implanted devices. However, most current implantable sensors are still based on polymeric material substrates, which tend to trigger an inflammatory response when implanted in the body [313]. This inflammatory response may damage surrounding tissues, increase the risk of infection, and potentially lead to more serious complications, thus limiting the biocompatibility of existing FGFET implantable biosensors. To address this issue, researchers have begun exploring more suitable alternative materials, such as silk protein (SF). Compared with conventional polymer materials, silk protein not only has superior mechanical properties but also offers excellent biocompatibility and biodegradability [320, 321]. Additionally, when SF is degraded by enzymes (e.g., proteases), its by-products exhibit low antigenicity and do not trigger inflammatory reactions [322, 323]. Leveraging these advantages, Bonaccini Calia et al. [324] developed flexible graphene deep neural probes (gDNPs) based on silk proteins. In a 12-week chronic biocompatibility test of deep brain structures in adult male Sprague Dawley rats, the gDNPs did not provoke significant inflammatory responses (Fig. 22b), indicating that gDNPs possess good biocompatibility and are suitable for long-term implantation into deep brain structures without causing significant tissue damage or neuroinflammatory responses. Furthermore, introducing biocompatible functional groups (e.g., carboxyl groups, amino groups, and polyethylene glycol) on the graphene surface can reduce the toxicity of graphene to cells and tissues, thereby improving the biocompatibility of FGFET-based implantable biosensors. Additionally, coating graphene surfaces with biocompatible materials (e.g., proteins, sugars, lipids, or polymers) can further reduce direct contact with biological systems, thereby lowering potential immune responses and toxicity, ultimately enhancing biocompatibility.

In conclusion, challenges still remain in achieving optimal long-term stability and biocompatibility for implantable biosensors, and their performance within the human body can be significantly improved through rational material selection, surface coating, surface functionalization, and structural optimization. This research direction is vital for medical applications and provides valuable insights and guidance for the broader development of biosensors in the future.

## Discussion, Conclusions, and Perspective

### Discussion: Strategies to Prepare the High-Performance FGFET-Based Flexible Biomedical Sensors

The design of flexible biomedical sensors must focus on three primary aspects: flexibility, sensing performance, and adaptability to complex substrates. Flexibility refers to the sensor's ability to maintain stable and reliable sensing performance under deformation, such as bending or stretching. Sensing performance encompasses the sensor's sensitivity, selectivity, and stability when detecting target biomolecules or physiological parameters. Adaptability to complex substrates means the sensor can operate normally and accurately detect target molecules or parameters in the intricate environment of a living organism. To construct high-performance FGFET-based biomedical sensors, it is crucial to enhance their mechanical properties and adaptability to complex substrates while maintaining excellent sensing performance.

Therefore, we first discuss the correlation between mechanical deformation and the sensing response signal of FGFET-based flexible biomedical sensors, with the aim of realizing flexible biomedical sensors with both sensing and flexibility properties. FGFET biosensors designed for physiological signal detection typically measure signals such as body temperature, neural activity, and some stresses through changes in charge amount, charge transport, and Dirac point in graphene, which are directly induced by external physical signals. In addition, in some of the FGFETs that monitor limb movements, they are coupled to nanogenerators, piezoelectric materials, etc., and physiological signals are detected by changes in carriers in graphene induced by the coupling materials during sensing of stress variations. As for the FGFET biosensors for biochemical signal detection, they generally achieve the detection of biochemical signals through the specific binding of probes (antibodies, aptamers, DNA, etc.) and target molecules on the graphene surface. Specifically, when the probe on the graphene surface captures the target molecule, a change in the carrier density inside the graphene is induced by charge transfer or electrostatic induction, which changes the FGFET current and the Dirac point position [160]. In conclusion, most flexible biosensors achieve the detection of physiological and biochemical signals by monitoring the changes of FGFET *I*_*ds*_ and the movement of Dirac points. Therefore, we briefly analyze the effect of deformation on the *I*_*ds*_ and Dirac points of FGFET biomedical sensors.

The Eq. (6) shows the change in the drain-source current of graphene caused by capturing the target molecule at the same drain-source voltage.

6 $$\Delta {I}_{ds}=\mu e\Delta n\frac{\text{W}}{L}{V}_{ds}$$

where *μ*, $\Delta n$, $e$, *W*, *L*, and ${V}_{ds}$ represent the graphene carrier mobility, the variation of graphene carrier density, electricity per unit elementary charge, channel length, channel width, and the source-drain voltage, respectively. In light of the aforementioned Equation, the both $\mu$ and $\Delta n$ parameters had an impact on the $\Delta {I}_{ds}$.

The Eq. (7) shows the change in the Dirac point of graphene caused by the biosignal:

7 $$\Delta {V}_{Dirac}=\frac{e\Delta n}{{C}_{T}}$$

where C_T_ is the total gate capacitance consists of the Helmholtz capacitance and the quantum capacitance of graphene itself in series, and *Δn* is the change in the carrier density of graphene. The variation of the ${V}_{Dirac}$ signal is mainly determined by the charge change of graphene caused by the biosignal and the gate capacitance of the graphene.

For flexible biomedical sensors, which are physiological signals reflected through deformation, they maintain a good correlation with at least one of *μ*, Δ*n*, or the total gate capacitance during deformation, and thus by observing ∆*I*_*ds*_ or Δ*V*_*Dirac*_ and thus the detection of physiological signals. As for the common flexible biosensors for detecting biochemical information, the Δ*n* induced by the biosignals solidly loaded on graphene remains essentially unchanged during deformation. According to the analysis in Sect. 3.3, the change in μ induced by deformation is smaller in FGFETs with fewer defects. In addition, the quantum capacitance of graphene changes when graphene is subjected to strain, however, when graphene is subjected to strain within 10%, the change in its quantum capacitance is so weak that it is negligible. The analysis shows that the effect of strain on the current and the Dirac point of the sensor is very weak during small deformation, and the detected signal mainly depends on the effect of biological signals on the electron transport properties of graphene.

Based on the above analysis, for flexible biomedical sensors detecting biochemical signals similar to biosensors on solid-state substrates, three aspects of stability, sensitivity and specificity should be addressed to obtain excellent sensing performance. Strategies to improve their stability include optimizing graphene mass, exploiting covalent bonding interactions, and stopping graphene degradation induced by aqueous solutions. To improve the sensitivity, overcoming the Debye length limitation, modifying the graphene surface and utilizing the 3D graphene structure are effective methods. To improve specificity, the use of specialized bioprobes and passivation strategies is also beneficial. Specific measures are detailed in "Recent Advances in Graphene Field-effect Transistor Towards Biological Detection" [325], which will not be repeated here. As for flexible biomedical sensors for monitoring physiological signals, improving their sensing performance mainly revolves around increasing the sensitivity to physiological signals, which can be achieved on the one hand by introducing impurity doping and defect engineering, material structure optimization, and composite material design to improve the perception of stress in graphene material itself, and on the other hand, coupling piezoelectric and friction electric effects, designing and fabricating graphene materials with high-voltage coefficients and excellent mechanical properties of materials and coupled with FGFETs. In addition, optimizing the nanostructure design can increase the surface area and stress concentration region, enhance the piezoelectric effect and electronic output signal, and thus improve the sensing performance.

Section 2.2's discussion of the mechanical characteristics of FGFETs shows that the maximum strain an FGFET-based biosensor surface can handle is contingent on the selection of materials and the design of the structure, with primary influences being the thickness and relative Young's modulus of the individual components. If the sensor's components are too thick and the Young's moduli of the working layer and the substrate differ significantly, the strain on the FGFET may exceed its failure threshold, potentially causing the graphene conductive channels to fail and the sensor to malfunction. Additionally, higher relative Young's modulus and thicker components can hinder the seamless bonding of FGFET biosensors to human skin or tissue. Therefore, it is crucial to minimize the sensor's thickness during the design and fabrication process. At the same time, while ensuring that the Young's modulus of the working layer is slightly higher than that of the substrate, the Young's modulus of the individual elements should not be excessively low. These considerations are essential for achieving high flexibility and reliable performance of FGFET biosensors under various mechanical deformations, allowing them to accurately measure fluctuations in physiological parameters and biomarker concentrations while adhering effectively to the skin or tissue. For wearable sensors fitted onto the body, the design must prioritize wear-resistance, biocompatibility, and durability to meet a wide array of user demands. On the flip side, for invasive sensors implanted in the body, the design must mitigate invasiveness and discomfort during use. Therefore, it is crucial to develop new substrate materials featuring biocompatibility or biodegradability. It is especially important to investigate conductive polymers composed of proteins or the extracellular matrix, as these materials can support cell growth without triggering an immune response. These considerations not only reduce environmental impacts significantly, but also ensure safety and comfort over extended implantation periods or long-term adherence to the skin.

For wearable and implantable FGFET biomedical sensors, it is crucial to detect biomarkers in human secretions and blood without preprocessing. However, the presence of various compounds in body fluids poses a challenge, as these compounds can cause non-specific adsorption on the graphene surface, severely interfering with detection signals. To address this issue, several strategies can be employed: i) Enhance specific interactions between the sensor and target molecules by functionalizing the FGFET surface. ii) Apply passivation treatments to eliminate non-specific adsorption signals. iii) Modify operating conditions such as temperature and pH to adapt to complex substrate environments, thereby improving sensor stability and accuracy. iv) Use advanced signal processing methods, such as noise filtering and signal amplification, to reduce interference from complex substrates.

In conclusion, constructing high-performance FGFET biomedical sensors requires a comprehensive approach that considers sensing performance, flexibility, and adaptability to complex substrates. By rationally designing sensor structures, selecting appropriate materials, and integrating biotechnological advancements, it is possible to achieve sensors with high sensitivity, selectivity, and excellent flexibility capable of functioning effectively in diverse and challenging environments.

### Conclusions

This paper reviews FGFETs and their recent advances in flexible biosensing. The focus is on the preparation strategies of FGFETs, including component materials as well as patterning techniques to guide the construction strategies of devices for different applications. The ability of FGFETs to sense strain varies greatly due to differences in graphene and FGFET structures as well as fabrication methods, providing versatility in FGFET applications. They are frequently included into wearable and implantable biomedical sensors that track physiological signals like body temperature, pulse, and nerve activity as well as biochemical ones like proteins, cytokines, glucose, and metal ions. The analysis of the effect of deformation on flexible biosensors shows that rational design of the sensor structure, selection of suitable materials and incorporation of biotechnological advances are key points in the preparation of high-performance flexible biosensors.

#### Perspective

While significant progress to date has greatly propelled FGFETs and their potential applications in the flexible biomedical sensing field, producing flexible biosensors that simultaneously meet requirements for high performance, self-powering, wireless transmission, and multifunctional integration remains a daunting challenge. Achieving widespread application in the biosensing domain necessitates continuously improving the overall quality of FGFETs and integrating them with advanced technologies. The following aspects represent opportunities and challenges in this field that should be carefully considered and addressed.

##### High-Performance FGFETs

Achieving high-performance FGFETs requires precise optimization of their electrical performance. Firstly, improving the synthesis method of graphene is crucial. For instance, optimizing the preparation parameters of CVD graphene can effectively reduce material defects, thereby significantly enhancing charge carrier mobility. Secondly, exploring new interface engineering techniques between graphene transmission channels and electrodes is vital. Utilizing novel metal alloys such as palladium-gold alloys or conductive polymers like polypyrrole can yield better contact interfaces, directly aiding in reducing contact resistance and enhancing device performance. Additionally, developing advanced microfabrication techniques to achieve nanoscale lateral dimension control and precise interlayer structure adjustments is crucial for accurately controlling the microstructure of FGFETs, which is paramount for device performance improvement. Lastly, innovative doping techniques provide an effective means to modulate the charge carrier concentration and types of graphene. For example, nitrogen or boron doping can effectively adjust the conductivity of graphene to meet specific requirements for conductivity in different applications. Through these precise electrical performance optimization methods, we can expect a significant improvement in the performance of FGFETs, enabling their widespread use in various high-end electronic devices.

Improving the flexibility of FGFETs requires efforts in material innovation and structural design. Firstly, developing new composite materials and nanostructures is crucial, as they enable FGFETs to maintain performance under extreme physical conditions such as significant bending, stretching, and compression. For instance, introducing highly ductile polymers as substrate materials can significantly enhance the flexibility of transistors. Secondly, flexibility can be enhanced by optimizing the geometric layout of transistors. For example, achieving curved arrangements of transistor arrays or using flexible connection designs like serpentine or mesh, or designing stress relief structures, can disperse stress when FGFETs undergo physical deformation, avoiding damage caused by localized stress concentration while maintaining electrical performance and enhancing flexibility. Additionally, optimizing the interface contact between graphene and flexible substrates can significantly increase their adhesion, thereby increasing overall durability and flexibility. This process involves careful selection of interface materials and advancement in processing techniques aimed at ensuring the stability and reliability of FGFETs in flexible applications. For example, atomic layer deposition (ALD) technology can form a uniform and tight interface layer between graphene and substrates, significantly enhancing adhesion between them. Through these measures, the flexibility of FGFETs can be effectively enhanced, making their application in wearable and implantable devices and flexible electronic products more widespread and practical.

##### Self-Powered FGFET-Based Biomedical Sensors

Due to their lightweight construction, flexible GFET biomedical sensors have brought revolutionary improvements to wearable technology. However, these sensors rely on external power sources and energy, limiting their application scope and flexibility. Developing self-powered systems is particularly important. By integrating advanced energy harvesting technologies such as triboelectric and thermoelectric technologies, we can not only ensure the persistent operation of FGFET sensors but also expand their applications in remote healthcare and mobile health monitoring. Nonetheless, a major challenge current self-powered sensors face is that the energy conversion efficiency still needs improvement. Capturing energy from the environment and converting it into electrical power may not be efficient enough, causing sensors to not operate stably under all conditions. Additionally, the cost of manufacturing self-powered sensors is high, especially those requiring complex technologies and manufacturing processes to enhance energy conversion efficiency and stability. This may limit their widespread application. Therefore, finding new materials and technologies to improve the performance and applicability of self-powered GFET biomedical sensors becomes crucial, helping to meet the practical needs of flexible GFET wearable biomedical sensors in real-world applications.

##### FGFET-Based Biomedical Sensors Combined with Digitization Techniques

FGFET sensors, based on their high sensitivity and rapid response, can accurately capture various biological signals from neural electrical activity to biochemical reactions. Integrating them with advanced signal collection and wireless transmission systems demonstrates great potential in enhancing the practicality and convenience of medical devices. By incorporating advanced microprocessing technology and intelligent algorithms in FGFET biomedical sensors, they do more than merely capture data; they also perform preliminary signal processing, filtering out useful information and reducing redundant data to further enhance the efficiency and accuracy of signal collection. Additionally, FGFET biomedical sensors are evolving towards low power consumption and high data transmission security in wireless transmission. Using the latest low-power Bluetooth technology, such as BLE 5.0, and Near Field Communication (NFC) technology, these sensors can seamlessly connect to smartphones or medical databases, enabling real-time health monitoring and remote diagnostics. As 5G and even future 6G networks continue to advance, it is expected that FGFET biomedical sensors will improve in high-speed data transmission and low-latency communication, making remote medical services more efficient and reliable. Another noteworthy advancement is that combining self-powered FGFET sensors with these advanced wireless data transmission technologies can achieve seamless data integration and real-time feedback, greatly enhancing the safety, convenience, and intelligence of personal health monitoring and remote medical services, providing users with higher quality medical solutions.

##### FGFET-Based Multifunctional Biomedical Sensors

FGFET multifunctional sensors, capable of simultaneously detecting multiple biological signals such as body temperature, heart rate, blood glucose, and disease biomarkers, will all be integrated into a single flexible device. This multifunctional integration not only helps save device space and lighten the burden on wearers but also provides users with more comprehensive health data, enabling a holistic monitoring of individual health conditions. However, the key to achieving simultaneous detection of multiple parameters in a multifunctional FGFET lies in differentiating between various stimuli. To address this challenge, selective sensing layers can be designed to differentiate between physical and chemical stimuli. Different materials have distinct electrical properties in response to different types of stimuli (e.g., strain, temperature, or chemical changes), which helps isolate and recognize specific signals [264, 326]. Additionally, integrating multimodal sensing functionality into the FGFET allows multiple parameters to be measured simultaneously within a single device, further enhancing the ability to distinguish between different stimuli [327, 328]. This approach ensures that different signals do not interfere with each other, providing users with more accurate and comprehensive health monitoring data. To further improve the detection accuracy of the FGFET, advanced signal processing techniques, such as inverse convolution algorithms, can be applied to the sensor's output signals. These techniques can effectively separate overlapping signals resulting from simultaneous physical and chemical changes, allowing for more accurate identification and analysis of the primary stimulus [329, 330]. Additionally, implementing calibration protocols and compensation algorithms is crucial. These methods not only correct cross-sensitivity between different stimuli but also ensure the purity and accuracy of the desired signal by predicting sensor response models and effectively compensating for the influence of the external environment on the sensing results [331]. While it is essential to differentiate between detection signals and improve accuracy, the portability and comfort of the sensor are equally important. Reducing the size and weight of the sensor is crucial to ensuring that the user is barely aware of its presence during daily wear. Therefore, advancements in miniaturization technologies and system-level integration strategies are necessary to tightly integrate GFETs with other electronic components, creating compact, multifunctional systems. Additionally, to fully utilize the potential of this hardware, intelligent software must be developed to operate the sensors for real-time data collection and analysis. This software would not only handle noise reduction, calibration, and data fusion processing but also transmit the processed data to the device’s display or wirelessly to other devices. As technology continues to advance, multifunctional wearable biomedical sensors are gradually becoming an indispensable part of enhancing people’s quality of life and preventing and treating diseases. With ongoing technological advancements and decreasing costs, it is foreseeable that such products will become more widespread, more convenient, and make a greater contribution to the health of all humanity.
